# Effects of a population-based, person-centred and integrated care service on health, wellbeing and self-management of community-living older adults: A randomised controlled trial on Embrace

**DOI:** 10.1371/journal.pone.0190751

**Published:** 2018-01-19

**Authors:** Sophie L. W. Spoorenberg, Klaske Wynia, Ronald J. Uittenbroek, Hubertus P. H. Kremer, Sijmen A. Reijneveld

**Affiliations:** 1 University of Groningen, University Medical Center Groningen, Department of Health Sciences, Community and Occupational Medicine, Groningen, Groningen, The Netherlands; 2 University of Groningen, University Medical Center Groningen, Department of Neurology, Groningen, Groningen, The Netherlands; King's College London, UNITED KINGDOM

## Abstract

**Objective:**

To evaluate the effects of the population-based, person-centred and integrated care service ‘Embrace’ at twelve months on three domains comprising health, wellbeing and self-management among community-living older people.

**Methods:**

Embrace supports older adults to age in place. A multidisciplinary team provides care and support, with intensity depending on the older adults’ risk profile. A randomised controlled trial was conducted in fifteen general practices in the Netherlands. Older adults (≥75 years) were included and stratified into three risk profiles: Robust, Frail and Complex care needs, and randomised to Embrace or care as usual (CAU). Outcomes were recorded in three domains. The EuroQol-5D-3L and visual analogue scale, INTERMED for the Elderly Self-Assessment, Groningen Frailty Indicator and Katz-15 were used for the domain ‘Health.’ The Groningen Well-being Indicator and two quality of life questions measured ‘Wellbeing.’ The Self-Management Ability Scale and Partners in Health scale for older adults (PIH-OA) were used for ‘Self-management.’ Primary and secondary outcome measurements differed per risk profile. Data were analysed with multilevel mixed-model techniques using intention-to-treat and complete case analyses, for the whole sample and per risk profile.

**Results:**

1456 eligible older adults participated (49%) and were randomized to Embrace (n(T0) = 747, n(T1) = 570, mean age 80.6 years (SD 4.5), 54.2% female) and CAU (n(T0) = 709, n(T1) = 561, mean age 80.8 years (SD 4.7), 55.6% female). Embrace participants showed a greater–but clinically irrelevant–improvement in self-management (PIH-OA Knowledge subscale effect size [ES] = 0.14), and a greater–but clinically relevant–deterioration in health (ADL ES = 0.10; physical ADL ES = 0.13) compared to CAU. No differences in change in wellbeing were observed. This picture was also found in the risk profiles. Complete case analyses showed comparable results.

**Conclusions:**

This study found no clear benefits to receiving person-centred and integrated care for twelve months for the domains of health, wellbeing and self-management in community-living older adults.

## Introduction

Older adults prefer to remain living at home for as long as possible–‘to age in place’–and to participate in society [1–3]. However, this preference is compromised by age-related health problems [4,5], leading to an increasing level of dependency and service-use, a growing sense of loss of control and insecurity, and the threat of ultimate relocation to an institution [6–9]. A major challenge is how to support older people to age in place in the face of increasing decline associated with ageing [6,7]. The current healthcare systems are insufficiently able to address these challenges for many ageing individuals and need to be reorganised in such a way that they promote ageing in place [10].

A model of increasing importance and popularity in healthcare reform is the Chronic Care Model (CCM) [11–13]. The CCM addresses the needs of chronically ill patients by offering comprehensive, person-centred, proactive and preventive care and support. It encourages patients to be informed and activated, meaning that they should formulate personal goals and a plan to improve their health, and should have the motivation, information, skills, and confidence necessary to deal with the consequences of their diseases [14]. Two randomised controlled trials on the CCM targeted older adults, but both have limitations regarding the study populations included as only frail older adults with a limited health status were included and not those who were still healthy [15,16]. In order to provide care and support to the total community-living population of older adults, the CCM can be combined with a Population Health Management (PHM) model. PHM models assess an entire population in a community and not just those in need of urgent care. PHM-based care and support can be targeted to individual needs by classifying population subgroups into risk profiles [17].

Embrace is an integrated care service based on the complete CCM and a PHM model (Kaiser Permanente [KP] Triangle) [13] targeting all community-living older adults [18]. Embrace’s goal is to support older adults to age in place by providing person-centred, integrated, proactive and preventive care and support. Embrace classifies older adults into three risk profiles based on the complexity of their care needs [19] and their level of frailty [20,21]. Care and support are tailored to the risk profile and the needs of the older adults. A qualitative study of Embrace has already shown that older adults felt safe, secure and more in control due to Embrace [6].

In this study, we intend to evaluate the effects of the population-based, person-centred and integrated care service Embrace on patient-reported outcomes at 12 months on three domains comprising health, wellbeing and self-management among community-dwelling older people.

## Methods

### Study design and setting

Between October 2011 and March 2013, we conducted a randomised controlled trial (RCT) with stratification into three risk profiles based on the level of frailty and complexity of care needs and balanced allocation within general practitioner (GP) practices to the intervention (Embrace) or care as usual (CAU) groups. The RCT was performed in three semi-rural municipalities in the province of Groningen (in the northern Netherlands). Participants were followed for twelve months between January 2012 and March 2013. The Medical Ethical Committee of the University Medical Center of Groningen has assessed the study proposal and concluded that approval was not required (Reference METc2011.108). The study was performed in accordance with the tenets of the Declaration of Helsinki [22]. All participants gave informed consent. The study protocol has been published previously [18]. The trial was registered at the Netherlands National Trial Register NTR3039 (http://www.trialregister.nl; see S1 and S2 Files). The CONSORT statement was followed to report the findings and the checklist is available as supporting information (S3 File).

### Study population and procedure

First, we invited all GPs working in the three municipalities to participate in the study. Recruitment stopped after fifteen GPs–proportionally distributed according to the size of the municipalities–agreed to participate as they had enough eligible participants to obtain the sample size needed. Next, all older adults aged 75 and over who were registered with one of the participating GPs and were living at home or in a home for the elderly, were invited to participate. Exclusion criteria at baseline were long-term admission to a nursing home (not just for rehabilitation), receiving an alternative type of integrated care and participating in another research study. Eligible participants received a letter from their GP with general information about Embrace and the study. After having provided informed consent, participants completed self-report questionnaires at baseline (T0: October-December 2011) and twelve months after starting (T1: January-March 2013), with support by a family member, friend or volunteer if needed. We sent reminders to non-respondents, followed by telephone calls to all persistent non-respondents. Respondents who submitted questionnaires with missing values were called by help desk assistants or visited by volunteers to complete the missing items.

### Stratified randomisation and blinding

We first stratified participants into one of three risk profiles, using results of the baseline assessment of complexity of care needs (measured using the INTERMED for the Elderly Self-Assessment [INTERMED-E-SA]) [19] and the level of frailty (measured using the Groningen Frailty Indicator [GFI]) [20]. These risk profiles are ‘Complex care needs’ for participants with complex care needs and at risk for assignment to a hospital or nursing home (INTERMED-E-SA ≥16), ‘Frail’ for participants at risk of complex care needs (INTERMED-E-SA <16 and a GFI ≥5) and ‘Robust’ for participants at risk for the consequences of ageing (INTERMED-E-SA <16 and GFI <5).

After stratification, we performed an anonymised and computerised balanced randomisation process within each GP practice. Therefore, participants were equally distributed within each GP practice to Embrace and CAU, taking into account predetermined patient characteristics deemed capable of affecting intervention outcomes, for example age, gender, number of chronic conditions and living situation [23]. Elderly Care Team members did not know if someone was randomised to CAU or had declined participation, but knew who was randomised to Embrace. Participating older adults were informed in writing whether they were assigned to Embrace or CAU. Data collectors (volunteers available when necessary for helping filling in questionnaires, and help desk assistants) were blinded for randomisation and stratification, as were the data analysts (SS and RU) until the point of data analysis. For practical reasons, the data manager was not blinded.

### Intervention: Embrace

Embrace (in Dutch: *SamenOud* [ageing together]) is a person-centred and integrated care service for community-living older adults. A multidisciplinary Elderly Care Team–consisting of the older adults’ GP, a nursing home physician [24] and two case managers (district nurse and social worker)–provides care and support to older adults. This care is in addition to care as usual. Before starting the intervention, team members followed an intensive training program (three days for the GPs and nursing home physicians, eight days for the case managers) that focused on working according to the Embrace principles and methods. Also, team members were coached during the intervention to support the cultural change in professionals’ deep-rooted working patterns [18].

The intensity, focus, and individual or group approach of the care and support depended on the participant’s risk profile. We invited all participants to follow a self-management support and prevention program focusing on staying healthy and independent for as long as possible. The program included regular Embrace community meetings, in which self-management abilities were encouraged and during which local healthcare and welfare organisations provided information on health maintenance, physical and social activities, and dietary recommendations. In addition, frail people and those with complex care needs received individual support from a case manager. They jointly developed an individual care and support plan targeting all health-related problems, which had to be agreed upon by the Elderly Care Team before implementation. The case managers monitored changes in the medical, psychosocial, or living situation, and navigated the plan’s delivery. The Elderly Care Team discussed and evaluated the participants’ health status and social situation in monthly meetings. If necessary, they took proactive steps in dialogue with participants to prevent deterioration. People with a ‘Robust’ profile were encouraged to contact the team in the event of changes in their health or living situation. Details of the implementation of Embrace have been published in the study protocol [18].

### Care as usual

The control group received care as usual as provided by their GPs and local health and community organisations. Municipalities are in charge of social care, disease prevention and health promotion. Once a health problem is found, patients enter the health care system–in most cases with a visit to their GP. In the Netherlands, GPs are family physicians who usually have a long-term relationship with their patients. They act as gatekeepers for specialised services in the Dutch healthcare system: patients need a referral to enter specialised medical care. The mean number of GP visits increases with age from six visits per year at age 45–64 to fifteen visits per year for people aged 75 years and older [25], and a regular GP visit takes about ten minutes [26].

### Patient-reported primary and secondary outcomes

We used eight different questionnaires to assess patient-reported outcomes in three domains: ‘Health,’ ‘Wellbeing’ and ‘Self-management,’ as these outcomes are important to ageing in place and to participation in society. Primary and secondary patient-reported outcomes differed per risk profile, as we expected problems to vary per profile (see Table 1) [18].

**Table 1:** Primary and secondary measurement instruments per risk profile.

	Complex care needs		Frail		Robust	
	Primary	Secondary	Primary	Secondary	Primary	Secondary
Health						
EQ-5D-3L	X		X		X	
INTERMED-E-SA	X			X		X
GFI	X		X			X
Katz-15		X		X		X
Wellbeing						
GWI		X		X		X
QoL		X		X		X
Self-management						
SMAS-30		X	X		X	
PIH-OA		X	X		X	

EQ-5D-3L = EuroQol-5D-3L including the EuroQol visual analogue scale; GFI: Groningen Frailty Indicator; GWI = Groningen Well-being Indicator; INTERMED-E-SA = INTERMED for the Elderly Self-Assessment; PIH-OA = Partners in Health scale for older adults; QoL = Quality of life; SMAS-30 = Self-Management Ability Scale version 2.

#### Health

The ‘Health’ domain included the outcomes ‘Health status,’ ‘Complexity of care needs,’ ‘Level of frailty’ and ‘Limitations in Activities of Daily Living (ADL).’ We measured *Health status* using the EuroQol-5D three-level version (EQ-5D-3L), which is a short self-report questionnaire measuring health in five dimensions [27,28] in combination with a visual analogue scale (EQ-VAS) [29].

We measured *Complexity of care needs* using the INTERMED-E-SA, which includes twenty questions in the biological, psychological, social and healthcare domains [19].

We measured *Level of frailty* in the physical, social, cognitive and psychological domains with the GFI self-report version (fifteen items) [20].

We measured *Limitations in ADL* using the Katz-15, which measures independence in six physical ADLs (PADL), seven instrumental ADLs (IADL) and two additional ADL items. We calculated ADL performance as the total number of disabilities [30]. Subscale scores were calculated for PADL and IADL.

#### Wellbeing

The ‘Wellbeing’ domain included ‘Wellbeing’ and ‘Quality of Life’ (QoL). *Wellbeing* was measured using the Groningen Well-being Indicator (GWI), covering eight sources of wellbeing in daily experiences: enjoying eating and drinking, sleeping and resting well, having good relationships and contacts, being active, managing oneself, being oneself, feeling healthy in body and mind, and living pleasantly. Participants had to indicate whether each source of wellbeing was important to them and, if so, whether they were satisfied with that source. The Well-being Satisfaction Score is the number of important sources divided by the number of satisfactory sources [unpublished manuscript].

We assessed *QoL* using two items derived from the self-perceived health questions of the RAND-36 [31]. The first item measured self-rated QoL, while the second item compared the current self-rated QoL with QoL a year earlier. Both questions are rated on a 5-point scale ranging from 1 to 5.

#### Self-management

The ‘Self-management’ domain included ‘Self-management ability’ and ‘Self-management knowledge and behaviour’. We assessed *Self-management ability* using the Self-Management Ability Scale (SMAS-30) version 2, which contains thirty items and six subscales. The total SMAS score was calculated as the average of the subscale scores [32,33].

We measured *Self-management knowledge and behaviour* with the culturally adapted and validated version of the Partners in Health scale (PIH) [34]: the PIH scale for older adults (PIH-OA) [35]. The PIH-OA includes three subscales measuring eight items on an 8-point scale. Originally, we defined the PIH as a secondary outcome measurement for quality of care. However, the new, adapted version–PIH-OA–measures self-management and is therefore included in the present study.

#### Adaptations to the trial protocol

When effects are found on an outcome measurement, follow-up analyses will be performed using the subscales of that particular measurement instrument–if applicable.

### Sample size

We used the primary outcome *Health status* (EQ-VAS) to calculate the sample size needed [18]. We considered a change in outcome of six points (SD 14 points) on the EQ-VAS of participants in the smallest sample, i.e. the risk profile ‘Frail,’ clinically relevant. With a power of 80% (α = 0.05, two-sided), a total number of 1062 older adults had to be included in the analysis. Taking into account an estimated non-response rate of 30% and a loss-to-follow-up rate of 30%, 2178 patients had to be invited to participate.

### Statistical analyses

Differences between respondents and non-respondents were tested using Chi-square tests for categorical variables and t-tests for continuous variables. Differences in reasons for dropout in the intervention and control groups were tested using Chi-square tests.

We assessed differences in change between the intervention and control groups using multilevel mixed-effects analyses with regression coefficients (B) with 95% confidence intervals (CI) at α = 0.05 (two-sided), with adjustment for age and sex. Individual measurements (difference score per outcome, calculated as the difference between the T0 score and T1 score) were included as the first level and GP practices as the second level. We estimated the clinical relevance of the effects using Cohen’s effect sizes (ES) for statistically significant differences (p<0.05), with an ES of ≥0.20 reflecting a clinically relevant difference [36,37].

We performed intention-to-treat (ITT) analyses [38] for the whole sample and per profile. Missing data were imputed at item level by multiple imputation techniques, with the fully conditional specification approach–which uses the Bayesian framework [39]. Variables group, risk profile, GP, sex, age, marital status, living situation, educational level, income and receiving help with completing the questionnaire were used as covariates of the missing predictor models, generating twenty imputed data sets. Missing scale scores due to loss to follow-up were imputed using the mean change in deterioration of completed cases, as we assumed that older adults deteriorate over time [40]. This process was performed per risk profile for each scale. ITT outcomes were compared with those of complete case analyses including participants having both T0 and T1 measurements [41].

We performed all analyses using SPSS Statistics version 23.0 and used Mplus version 7.1 to impute the data.

## Results

### Participants

**Fig 1:**
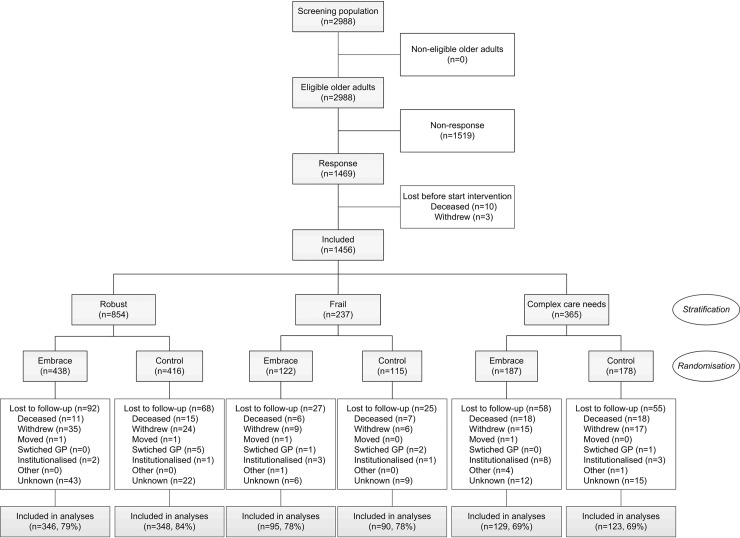
CONSORT flow diagram of the Embrace study.

Fig 1 presents the flow of participants in the study. We included 1456 of the 2988 eligible older adults in the study and analyses (48.7%). The main reasons for non-participation included poor health or having a partner with poor health, good health, questionnaire length and lack of interest. Non-respondents differed from respondents (all p-values <0.01) regarding gender (more women declined to participate), age (oldest older adults consented less often) and degree of urbanisation (more older adults living in rural areas declined to participate) (S1 Table).

Table 2 shows the baseline characteristics of participants. There were no statistically significant differences in the baseline characteristics between Embrace and CAU. After twelve months, 570 (76.3%) Embrace recipients and 561 (79.1%) CAU recipients completed the follow-up questionnaire. Dropouts (Embrace n = 177,23.7%; CAU n = 148, 20.9%) were significantly (all p-values <0.01) older, more frail, with more complex care needs and with poorer health. There were no significant differences in attrition rates between Embrace and CAU for the whole sample and per profile.

**Table 2:** Baseline characteristics of participants (n = 1456). Values are numbers (percentages) unless stated otherwise.

	Whole sample				Complex care needs				Frail				Robust			
	(n = 1456)				(n = 365)				(n = 237)				(n = 854)			
	Embrace		CAU		Embrace		CAU		Embrace		CAU		Embrace		CAU	
	(n = 747)		(n = 709)		(n = 187)		(n = 178)		(n = 122)		(n = 115)		(n = 438)		(n = 416)	
Age in years, mean (SD)	80.6	(4.5)	80.8	(4.7)	81.8	(4.6)	81.5	(4.9)	81.6	(5.1)	82.8	(5.5)	79.9	(4.0)	79.9	(4.1)
Female	405	(54.2)	394	(55.6)	121	(64.7)	115	(64.6)	82	(67.2)	80	(69.6)	202	(46.1)	199	(47.8)
Widowed/divorced/single	320	(42.8)	290	(41.0)	87	(46.5)	79	(44.4)	77	(63.1)	72	(63.2)	156	(35.6)	139	(33.5)
In sheltered accommodation/home for the elderly	93	(12.5)	99	(14.0)	37	(19.9)	40	(22.6)	20	(16.4)	26	(22.8)	36	(8.3)	33	(8.0)
Low educational level^1^	370	(49.9)	374	(53.4)	106	(57.0)	116	(66.3)	66	(54.1)	69	(60.0)	198	(45.7)	189	(46.0)
Low income^2^	261	(44.1)	231	(42.4)	80	(54.1)	77	(54.2)	53	(55.8)	51	(54.8)	128	(36.7)	103	(33.2)
Number of chronic conditions, median (IQR)	2	(1–3)	2	(1–3)	3	(2–5)	3	(2–5)	3	(1–4)	3	(2–4)	1	(1–2)	1	(1–2)
Receiving home care	89	(12.1)	69	(9.8)	47	(26.4)	42	(23.9)	24	(20.0)	14	(12.4)	18	(4.1)	13	(3.2)
Receiving help with filling in the questionnaire	243	(32.8)	245	(35.0)	99	(53.8)	106	(60.2)	48	(39.3)	43	(37.7)	96	(22.1)	96	(23.4)

CAU = Care as usual; IQR = Interquartile range; SD = Standard deviation.

^1^ Low: (Less than) primary school or low vocational training.

^2^ Low: <€1350 per month.

Values are based on complete data. There were no significant differences between CAU and Embrace–neither for the whole sample nor per risk profile. This was tested using independent t-tests for continuous variables, Chi-square tests for categorical variables, and Mann-Whitney U tests for non-normally distributed continuous variables and ordinal variables.

### Differences in effects between Embrace and CAU

#### Whole sample

We found no clear beneficial effects of Embrace in the whole sample as compared to CAU. Regarding the *Health* domain, Embrace participants showed a significantly greater deterioration in ADL (p = 0.047, ES = 0.10) and PADL performance (p = 0.011, ES = 0.13) compared to CAU–although these effect sizes indicated not clinically relevant changes. We found no differences in the changes observed between Embrace and CAU regarding *Wellbeing* outcomes (p>0.05, ES≤0.05. Regarding *Self-management*, Embrace participants showed a significantly greater improvement in the ‘Knowledge domain of self-management knowledge and behaviour’ compared to CAU, but this difference did not reach clinical relevance (p = 0.009, ES = 0.14) Table 3 and S2 Table).

**Table 3:** Patient-reported outcomes at 12-month follow-up in the Embrace study: Overview of the results of the intention-to-treat multilevel analyses for the whole sample and per risk profile.

			Whole sample				Complex care needs				Frail				Robust			
			(n = 1456)				(n = 365)				(n = 237)				(n = 854)			
			Embrace	CAU			Embrace	CAU			Embrace	CAU			Embrace	CAU		
	Scale scores (range)	Higher score*	Mean change	Mean change	p-value†	ES	Mean change	Mean change	p-value†	ES	Mean change	Mean change	p-value†	ES	Mean change	Mean change	p-value†	ES
**Health**	** **	** **																
EQ-5D-3L	-0.33–1.00	+	0.00	0.00	0.670	0.02	-0.02	-0.01	0.521	0.07	-0.02	0.0	0.223	0.16	0.01	0.01	0.630	0.03
EQ-VAS	0–100	+	-0.5	-0.6	0.878	0.01	-0.1	1.6	0.323	0.10	-1.7	-3.0	0.387	0.11	-0.4	-0.9	0.511	0.05
INTERMED-E-SA	0–60	-	-0.1	-0.2	0.597	0.03	-1.9	-2.6	0.149	0.15	1.4	1.3	0.608	0.06	0.3	0.5	0.540	0.04
GFI	0–15	-	0.1	0.1	0.998	0.00	0.1	0.0	0.552	0.06	-0.6	-0.7	0.586	0.07	0.4	0.5	0.411	0.06
Katz-15^1^	0–15	-	0.35	0.19	**0.047**	0.10	0.58	0.33	0.204	0.13	0.28	0.39	0.660	0.06	0.26	0.08	**0.035**	0.14
PADL	0–6	-	0.14	0.06	**0.011**	0.13	0.32	0.14	0.058	**0.20**	0.14	0.10	0.561	0.08	0.07	0.01	0.089	0.12
IADL^2^	0–7	-	0.19	0.13	0.185	0.07	0.27	0.16	0.363	0.10	0.11	0.25	0.355	0.12	0.18	0.08	0.063	0.13
**Wellbeing**	** **	** **																
GWI SF Score^3^	0–1	+	-0.02	-0.02	0.892	0.01	-0.02	-0.03	0.512	0.07	-0.04	0.0	0.478	0.09	-0.02	-0.02	0.900	0.01
QoL general	0–5	-	0.08	0.10	0.636	0.02	0.17	0.14	0.587	0.06	0.12	0.09	0.818	0.03	0.03	0.08	0.289	0.07
QoL vs 1 year ago	0–5	-	0.08	0.04	0.320	0.05	-0.04	0.01	0.471	0.08	0.11	0.17	0.425	0.10	0.13	0.02	**0.018**	0.16
**Self-management**	** **	** **																
SMAS-30	0–100	+	-1.1	-0.8	0.411	0.04	-2.0	0.2	**0.015**	**0.26**	-0.4	-0.7	0.705	0.05	-0.9	-1.2	0.664	0.03
INIT	0–100	+	-2.3	-2.5	0.709	0.02	-2.8	-2.1	0.530	0.07	-1.7	-2.3	0.658	0.06	-2.2	-2.8	0.485	0.05
SE	0–100	+	-0.8	-0.9	0.455	0.04	-2.1	1.7	**0.020**	**0.24**	0.0	-1.3	0.619	0.07	-0.4	-2.0	0.585	0.04
INVEST	0–100	+	-1.1	0.0	0.802	0.01	-1.3	0.8	**0.005**	**0.30**	-0.3	0.5	0.412	0.11	-1.2	-0.4	0.068	0.13
POSITIVE	0–100	+	-0.2	0.2	0.542	0.03	-0.2	1.2	0.217	0.13	-0.3	0.5	0.680	0.05	-0.1	-0.3	0.835	0.01
MULT	0–100	+	-0.8	-0.4	0.124	0.08	-1.9	1.1	0.126	0.16	-1.1	-1.7	0.609	0.07	-0.2	-0.6	0.383	0.06
VAR	0–100	+	-1.3	-0.8	0.461	0.04	-3.2	-1.3	0.177	0.14	1.2	0.3	0.450	0.10	-1.2	-0.8	0.649	0.03
PIH-OA^4^	8–64	+	0.8	0.4	0.285	0.06	1.1	1.1	0.976	0.00	1.7	-0.8	**0.020**	**0.31**	0.4	0.4	0.936	0.01
Knowledge	2–16	+	0.8	0.3	**0.009**	0.14	0.8	0.3	0.113	0.17	1.0	-0.2	**0.015**	**0.32**	0.7	0.4	0.245	0.08
Management	2–16	+	0.1	0.0	0.691	0.02	0.2	0.2	0.969	0.00	0.2	-0.2	0.398	0.11	-0.1	-0.1	0.965	0.00
Coping	4–32	+	0.0	0.1	0.659	0.02	0.1	0.6	0.336	0.10	0.6	-0.4	0.119	**0.21**	-0.2	0.0	0.355	0.06

CAU = Care as usual; EQ-5D-3L = EuroQol-5D-3L; EQ-VAS = EuroQoL-5D visual analogue scale; ES = Effect size *d*, thresholds <0.2 trivial, ≥ 0.2–0.5 small, ≥0.5–0.8 medium, ≥ 0.8 large; GFI = Groningen Frailty Indicator; GWI SF Score = Groningen Well-being Indicator Satisfaction Score; IADL = Instrumental Activities of Daily Living; INIT = Taking initiatives subscale; INTERMED-E-SA = INTERMED for the Elderly Self-Assessment; INVEST = Investment behaviour subscale; MULT = Multi-functionality of resources subscale; PADL = Physical Activities of Daily Living; PIH-OA = Partners in Health scale for older adults; POSITIVE = Positive frame of mind subscale; QoL = Quality of life; SE = Self-efficacy beliefs subscale; SMAS-30 = Self-Management Ability Scale version 2; VAR = Variety in resources subscale.

* + Higher score means improvement;—higher score means deterioration.

† Values are corrected for age and sex; bold values indicate p<0.05.

^1^ Percentage of missing items at baseline before imputation 7.4%

^2^ Percentage of missing items at baseline before imputation 5.4% and 6.1% at follow-up.

^3^ Percentage of missing items at baseline before imputation 12.7%

^4^ Percentage of missing items at baseline before imputation 5.7%

Bold text and orange filling: Significant (p<0.05) or clinically relevant (ES ≥0.20) deterioration

Bold text and green filling: Significant (p<0.05) or clinically relevant (ES ≥0.20) improvement

#### Complex care needs

We found no significant differences in the changes observed in the domains of *Health* and *Wellbeing* after twelve months between Embrace and CAU. However, there was a significant and clinically relevant difference in change in the *Self-management* outcomes ‘Self-management abilities,’ ‘Self-efficacy beliefs’ and ‘Investment behaviour’, as Embrace participants performed worse after twelve months, whereas those in CAU showed a small improvement (Table 3 and S3 Table).

#### Frail

We found no significant differences in the change observed between Embrace and CAU regarding *Health* and *Wellbeing*, but Embrace participants did show a significantly greater improvement in the ‘Self-management knowledge and behaviour’ *Self-management* outcome, as well as in its ‘Knowledge’ domain, compared to a deterioration for those in CAU (Table 3 and S4 Table).

#### Robust

We found no significant differences in the *Health* domain, except for significantly worse ADL performance compared to CAU–although this difference was not clinically relevant. Furthermore, Embrace participants showed a significantly larger deterioration in the *Wellbeing* outcome ‘QoL comparison item’ compared to CAU, but this difference was not clinically relevant either. We found no differences in the changes observed between groups regarding *Self-management* (Table 3 and S5 Table).

### Missing data and sensitivity analyses

Missing scale scores ranged from 0.0% to 12.7%, with 37 of the 42 scales and subscales having less than 5.0% missing values (Table 3). Sensitivity analyses with complete cases showed the same pattern of results, except for 1) a significant deterioration in PADL performance of the complex Embrace participants, and 2) a no longer significant–but still clinically relevant–improvement on the total PIH-OA score for the frail Embrace participants (S6–S10 Tables).

## Discussion

This RCT examined the effects of ‘Embrace,’ a person-centred and integrated care service for older adults, for the total sample and by respective risk profiles. We found no clear clinically relevant changes after receiving twelve months of care and support by Embrace on health, wellbeing and self-management in the total sample of community-living older adults and neither in the risk profiles. Overall, Embrace participants showed a greater–but clinically irrelevant–improvement in self-management knowledge and a greater–but clinically irrelevant–deterioration in ADL compared to CAU. This heterogeneous picture was also found in the risk profiles.

### Interpretation of findings

The care and support offered by Embrace had fewer beneficial effects–and sometimes even unbeneficial effects–on the domains of *Health*, *Wellbeing* and *Self-management* than we anticipated, which confirms the heterogeneous outcomes previously reported in RCTs on integrated care programs for community-living older adults [15, 16, 42–56].

Our finding of no clear benefits for Embrace on the outcomes measured could be due to the duration of the intervention, the nature of the intervention, the selection of outcomes or methodological limitations.

Firstly, the intervention may not have worked or may not yet have worked. We may have been dealing with an investment effect [57], as this multifaceted and complex intervention requires a cultural change in professionals’ deep-rooted working patterns, which could take more time than only twelve months despite an intensive training and coaching program before and during the intervention. Assessment among participating professionals of whether the care and support provided was in accordance with the Chronic Care Model underlined this assumption. We found a clinically relevant increase in the perceived level of implementation of integrated care from a ‘basic level’ at the start to a ‘reasonably good level’ after twelve months–indicating clinically relevant improvements with room for further improvement [58]. Evaluation of effects in the longer term is therefore needed, as well as follow-up coaching for further support of the cultural change in professionals’ working behaviour and evaluation of protocol adherence.

Secondly, the contrast between our intervention and CAU may have been too small to detect differences over the first twelve-month period. The Dutch healthcare system is already of a quite high standard, as all inhabitants have health insurance and healthcare is easily accessible, leaving little room for improvement [59]. This was confirmed by our finding that only the frail Embrace participants showed a significant increase in self-management knowledge and behaviour. These participants had received little or no care before the start of the intervention, in contrast with the complex participants, the majority of whom already received home care.

Thirdly, we had to deal with the heterogeneity and instability of the older population, which increased measurement error and thus reduced the likelihood of observing effects [60].

Fourthly, the measurement instruments for health and wellbeing may not have been specific enough for this type of intervention and may not have been sensitive enough to detect changes in clinical practice [61]. This could explain why we did find effects on two specifically developed measurement instruments: the PIH-OA, which is a version of the PIH for the evaluation of self-management knowledge and behaviour in older adults [35], and the PAIEC [62], which is used in another Embrace study for evaluation of perceived quality of integrated care and support [58].

### Strengths and limitations

The strengths of this study are its design–a RCT targeting all community-living older adults–and its stratification of participants into risk profiles, thereby enabling professionals to provide person-centred care and support. Moreover, we were able to perform predefined subgroup analyses to examine the effect of integrated care in subgroups at a higher risk of deterioration [63].

We must also acknowledge some potential limitations. We randomised within GP practices, which increased the risk of contamination. Although we instructed GPs to provide care as usual to patients who were not assigned to the intervention, we may have underestimated the effect on CAU participants. However, regular GP visits are brief and only take about ten minutes [26], with little time to discuss the topic of concern–let alone other health-related topics [64]. Moreover, CAU participants did not receive any additional support that was part of the intervention. Furthermore, a potential limitation is the non-response rate at baseline of about 50%. The differences between respondents and non-respondents concerning gender, age and degree of urbanisation may limit the generalisability of our findings to some extent.

### Implications for practice, policy and research

The present study showed that receiving twelve months of integrated care has no clear beneficial effect on patient-reported outcomes. Based on these results, the implementation of integrated care services for older adults cannot be recommended. However, a parallel study on Embrace showed that perceived quality of care improved [58]. Moreover, in a qualitative study of Embrace, older adults indicated that they felt safe and secure due to Embrace care and support [6]. These results could contribute to decision-making and show the need for mixed method evaluations [65]. Mixed method evaluation could also offer an explanation for the absence of clear effects in the present study [65]. Furthermore, future research should focus on the long-term effects of Embrace and should use outcomes–for example on dependency, age-related fears and coping–and specifically developed measurement instruments appropriate for this older population and type of intervention. A future cost-effectiveness study could help policy makers and professionals decide whether to implement Embrace. The effects of Embrace should also be evaluated in other geographical areas and in other cultures with different healthcare systems. Finally, stratification into risk profiles was the starting point for delivering care and support at a suitable care intensity level. Future studies could also target different risk profiles.

### Conclusion

The present study showed that receiving twelve months of person-centred and integrated care and support from Embrace has no clear beneficial effect on patient-reported health status and neither on wellbeing and self-management outcomes. Future research should provide insight into the long-term effects of Embrace. Moreover, specifically developed measurement instruments suitable for the target population and intervention should be used in future studies. As this is the first CCM-based RCT to include a population-based sample of community-living older adults, it contributes to the design of future research on population-based integrated care.

## Supporting information

S1 File Original Research Protocol_English.pdf. (PDF)

S2 File Original Research Protocol_Dutch.pdf. (PDF)

S3 File CONSORT Checklist.pdf. (PDF)

S1 Table Characteristics of participants and non-participants. Values are numbers (percentages) unless stated otherwise.
(DOCX)

S2 Table Patient-reported outcomes at 12-month follow-up in the Embrace study: Detailed results of the intention-to-treat multilevel analyses using data from the whole sample (n = 1456). (DOCX)

S3 Table Patient-reported outcomes at 12-month follow-up in the Embrace study: Detailed results of the intention-to-treat multilevel analyses using data from participants with the risk profile complex care needs (n = 365). (DOCX)

S4 Table Patient-reported outcomes at 12-month follow-up in the Embrace study: Detailed results of the intention-to-treat multilevel analyses using data from participants with the risk profile frail (n = 237). (DOCX)

S5 Table Patient-reported outcomes at 12-month follow-up in the Embrace study: Detailed results of the intention-to-treat multilevel analyses using data from participants with the risk profile Robust (n = 854). (DOCX)

S6 Table Patient-reported outcomes at 12-month follow-up in the Embrace study: Overview of the results of the complete case multilevel analyses for the whole sample and per risk profile. (DOCX)

S7 Table Patient-reported outcomes at 12-month follow-up in the Embrace study: Detailed results of the complete case multilevel analyses using data from the whole sample (n = 1456). (DOCX)

S8 Table Patient-reported outcomes at 12-month follow-up in the Embrace study: Detailed results of the complete case multilevel analyses using data from participants with the risk profile complex care needs (n = 365). (DOCX)

S9 Table Patient-reported outcomes at 12-month follow-up in the Embrace study: Detailed results of the complete case multilevel analyses using data from participants with the risk profile frail (n = 237). (DOCX)

S10 Table Patient-reported outcomes at 12-month follow-up in the Embrace study: Detailed results of the complete case multilevel analyses using data from participants with the risk profile Robust (n = 854). (DOCX)
