# Supplementary material for: Effects of a population-based, person-centred and integrated care service on health, wellbeing and self-management of community-living older adults: A randomised controlled trial on Embrace
Source: PLoS One. 2018 Jan 19;13(1):e0190751. doi: 10.1371/journal.pone.0190751 (PMC5774687; doi:10.1371/journal.pone.0190751)
Supplement: S1 File — (PDF) [file pone.0190751.s001.pdf]

Note to the editor and reviewers:

At this moment, we do not have a complete English version of the original study protocol and analysis plan as submitted to the Ethics committee (2011). Therefore, we provide relevant translations of the original Dutch protocol sections. We refer for each translated section to the page number of the original Dutch version, which we have included as an appendix as well.

Concept

RESEARCH PROTOCOL

Embrace

Redesign of care delivery: towards person-centered, coherent, proactive, and preventive care and support for older adults

April 2011

Protocol title: Embrace

Redesign of care: towards person-centered, coherent,
proactive, and preventive care and support for older adults.

Short title	*Embrace*
Version	*1*
Date	*April 2011*
Coordinating researchers / project leader	*Prof. Dr. S.A. Reijneveld*<br>*Prof. Dr. H.P.H. Kremer*
Lead researcher / executor	*Dr. K. Wynia*<br>*Researcher Integrated Care*<br>*Health Sciences UMCG*<br>*k.wynia@med.umcg.nl*<br>*050-3632005*
Commissioning party	*ZonMW*<br>*PO Box 93245*<br>*2509 AE The Hague*
Independent physician	*Prof. Dr. O.R. Brouwer*<br>*Department of Neurology, University Medical Center Groningen*

PROTOCOL SIGNATURES

Name	Signature	Date
Head of department<br>*Prof. Dr. S.A. Reijneveld*<br>*Head of Department of Health Sciences*		20-04-11
Lead researcher<br>*Dr. K. Wynia*<br>*Researcher Integrated Care*		19-04-11

CONTENT

LIST OF ABBREVIATIONS 6
LIST OF CONCEPTS 6
SUMMARY 10
1. INTRODUCTION AND RELEVANCE 12
1.1 Relevance 15
1.1.1 Case management project 15
1.1.2 Relevance for stakeholders 16
2. AIMS 18
3. RESEARCH DESIGN 20
3.1 Research design 20
3.2 Length of research 20
3.3 Setting 20
4. STUDY POPULATION 21
4.1 Population 21
4.2 Inclusion criteria 21
4.3 Exclusion criteria 21
4.4 Sample size 22
5. TREATMENT OF PARTICIPANTS 24
5.1 Theoretical background 24
5.1.1 The Chronic Care Model (CCM) 24
5.1.2 The Kaiser Permanente (KP) Triangle 24
5.2 Intervention 24
5.2.1 Part 1: Organisatie of care – Elderly Care Team (ECT) 26
5.2.2 Part 2: Organisation of care – Care- en case management 26
5.2.3 Part 3: Self-management support and prevention 29
5.3 Ususal care 32
6. METHODS 33
6.1 Outcome measures and measurement instruments 33
6.1.1 Older adult – Primary outcome 33
6.1.2 Older adult – Secundary outcomes 33
6.1.3 Caregiver – Primary outcome 35
6.1.4 Caregiver – Secundary outcomes 35
6.1.5 Quality of care – Primary outcome 35
6.1.6 Quality of care – Secundary outcomes 35
6.1.7 Health care use and costs – Primary outcome 36
6.2 Randomisation, blinding, and treatment allocation 40
6.3 Study procedures 42
6.4.1 Data collection T0 42
6.4.2 Data collection T1 44
6.4.3 Data collection $T_{variable}$ 46
6.4.4 Intervention 46

6.5 Withdrawal from the study ... 46
6.5.1 Specific criteria for withdrawal from the study ... 47
6.6 Replacement of participants after withdrawal from the study ... 47
6.7 Follow-up of participants who withdrew from the study ... 47
6.8 Premature ending of the study ... 47
7 SAFETY ... 48
7.1 Section 10 WMO ... 48
7.2 Serious incidents ... 48
7.3 Follow-up of serious incidents ... 48
8 STATISTICAL ANALYSES ... 49
8.1 Descriptive statistics ... 49
8.2 Univariate analyses ... 49
8.3 Multivariate analyses ... 49
9 ETHICAL ASPECTS ... 50
9.1 Codes ... 50
9.2 Recruitment and informed consent ... 50
9.2.1 Older adults ... 50
9.2.2 Carers ... 51
9.3 Objection by those who are unable to give informed consent ... 51
9.4 Advantages and disadvantages for respondents (older adults and carers) ... 51
10 ADMINISTRATIVE ASPECTS AND PUBLICATION ... 53
10.1 Processing and storage of data and documents ... 53
10.2 Amendements ... 53
10.3 Yearly progress report ... 53
10.4 Final study report ... 53
11 REFERENCES ... 55

2. AIMS *(page 14)*

Main objective of the new care model *Embrace* is to achieve demand-oriented, integrated, proactive, and preventive care by which older persons' complexity of care needs will reduce, wellbeing will increase and (overall) service use and health care costs will decrease or at least remain equal.

The major aims of *Embrace* are:

1) Fifteen well-trained and properly functioning Elderly Care Teams (ECTs), located in fifteen general practitioner practices in three municipalities in the province of Groningen [the Netherlands].
2) The ECTs have a strong network, including local authorities, care providers (primary and secondary) and health insurance companies.
3) The ECTs have provided integrated, proactive, and preventive care and support to participating older adults (75 years and older).
4) Regarding older adults with highly complex care needs, the complexity of care needs will reduce or a stay in a hospital or nursing home will be prevented or delayed.
5) Regarding older adults without complex care needs Embrace will prevent – as far as possible – the development of complex care needs.
6) For (some) older adults, self-management abilities will be increased.

Central research question in this transitional experiment is: *What are the effects of the new care model Embrace for older adults aged 75 years and older on:*

1) Complexity of care needs, frailty, and wellbeing of the older adults.
2) Self-management ability and the self-reliance of the older adults.
3) Caregivers' strain.
4) Quality of care.
5) Health care utilization and health care costs.

3. RESEARCH DESIGN *(page 15)*

3.1 Research design

The central research question will be answered using a pre-stratified Randomized Controlled Trial with balanced allocation.

3.2 Duration of the research

The intervention period of the study on the effects of the new care model *Embrace* will a have duration of 12 months, starting December 1st 2011.

3.3 Setting

The intervention will take place in Stadskanaal, Veendam, and Pekela [municipalities] in the province of Groningen [the Netherlands]. The general practitioner practices in these municipalities are the foundation of the Elderly Care Teams (ECTs). These ECTs work in close relationship with the community organizations and primary and secondary health care organizations within these municipalities.

4. STUDY POPULATION *(page 16)*

4.1 Population

The study focuses primarily on people aged 75 years and older who are registered with one of the fifteen participating general practitioner (GP) practices. If present, caregivers of older adults are also eligible for participation.

- *Older adults:* all people aged 75 years and older from a GP practice. Older adults in this age group are known to have a high risk of being or becoming frail, and their complexity of care needs can increase suddenly and drastically.
- *Caregivers:* the main caregiver of the older adult. Usually this is the partner, but it can also be a neighbor. A large and important part of care for older adults is provided by caregivers. It is known that with an increase in burden of care, the risk of caregiver straining increases as well. [10-11] This also increases the risk of institutionalization of the older adult. Support for informal caregivers, which is part of the intervention, could help delaying or preventing unwanted and (relatively expensive) long-term admissions.

4.2 Inclusion criteria

- *Older adults:* 75 years and older, living at home or living in a home for the elderly, and registered with one of the 15 participating GP practices.
- *Caregivers:* The older adult (care recipient) participates in the intervention group in the study. NB. The main caregiver is the one who has the largest share in caring for the participating older adult. This is usually the partner, sometimes a close relative. Inclusion of caregivers in the control group has been considered. However, it was decided not to include them in this study. This was due to the complicated procedure of identification of these potential caregivers via the older adults with complex care needs (and through additional questions in the questionnaire) (see also p. 30 Caregivers). Caregivers of older adults with complex care needs or with increased frailty in the intervention group will be asked to participate by the case managers during history taking.

4.3 Exclusion criteria

- *Older adults:* Long-term stay in a nursing home, receiving a (different) type of integrated care or participating in another study.
- *Caregivers:* Participation in another study. When the main caregiver is the partner of the participant and he/she also has complex care needs, this caregiver can be included as ’older adult’.

4.4 Sample size

The power of this study was calculated to detect differences between the intervention and control group in the mean scores on the Visual Analogue Scale (VAS) of the EQ-5D [12] with a power of at least 80% (beta of 20%). Assuming an Intraclass Correlation Coefficient of 0.10, a minimum of 108 older adults per group is needed. Assuming that 20% of all older adults aged 75 years and older have increased case complexity, taking into account a non-response rate of 30%, and loss-to-follow-up (death or admission to a nursing home) of 30%, a total of 2202 older adults need to be approached to include those 108 older adults:

- Assuming two groups (intervention and control group) = 1101 older adults need to be approached per group
- Response rate of 70%: inclusion of 771 older adults per group
- Loss-to-follow-up of 30%: 540 older adults who can be included in the analyses, per group
- 20% in care- and case management groups = 108 older adults
- 80% in the self-management support and prevention group = 432 older adults

With an expected inclusion of 771 older adults in the study and randomized to both groups, the expected distribution of older adults at the start of the interventions (loss-to-follow-up excluded) will be as follows:

- 154 older adults for care and case management = 39 older adults case management and 115 older adults care management
- 617 older adults for self-management support = 123 older adults individual counseling and 493 older adults group interventions

5. TREATMENT OF PARTICIPANTS *(page 18-23)*

This chapter first outlines the theoretical embedding of the new care model Embrace (§ 5.1). Next, the intervention for the older adults is described (Section 5.2), and two components of the Chronic Care Model (CCM) are specified (§5.2.1 - §5.2.3). Finally, care as usual as received by the older people in the control group is described (§5.3).

…

6. METHOD *(page 24)*

6.1 Outcome measurements and measurement instruments

The questionnaires are shown in Table 1, p. 28

6.1.1 Older adults – primary outcome

The primary outcome for the older adults is complexity of care needs (INTERMED-score, as measured by the triage instrument [1]).

- INTERMED: This questionnaire measures the complexity of the (health) care needs in four areas: biological, psychological, social needs, and the care system, from different time perspectives: past, present, and future. The INTERMED was designed for use in both inpatient and outpatient settings. The original instrument is filled in by caregivers and has been used and validated in many countries. [15-18] The psychometric properties of the instrument are supported by the results of various studies. [17, 19-22] The instrument was adapted to a self-report version for older adults.

6.1.2 Older adults – Secondary outcomes

Secondary outcomes are the level of frailty (Groninger Frailty Indicator [GFI]) and well-being (Well-being List) (both part of the triage-instrument), well-being as measured with the Minimal Data Set (MDS) (RAND 36-item Health Survey (RAND-36) and Cantrill's Ladder), self-management abilities (Self-Management Ability Scale [SMAS-30]), self-management knowledge (Partners In Health Scale [PIH scale]) and quality of life (EQ-5D in the MDS).

- GFI: This questionnaire measures frailty and consists of fifteen items, is easy to fill out by older adults, and has good predictive validity. The instrument is being used in a wide variety of elderly care settings, both nationally and internationally. [13-14]
- Well-being List: This list determines the things that matter to a lot of people in life. This new instrument is a generic tool that can measure the well-being of older adults in different situations. It consists of eight items in terms of everyday experiences (i.e. enjoying food and drinks; sleeping and resting, enjoyable relationships and contacts). The older adults are asked whether they find an item important or not, and, if that is the case, whether they are satisfied in that area of their daily lives.
- MDS Care recipient – combination of questionnaires: The intention is to be able to compare the results of all projects that are part of the National Care for the Elderly Program (NPO). For that purpose, a research tool was developed by the NPO and made mandatory for all participating projects: the Minimal Data Set (MDS). The MDS contains several validated research questionnaires for the care recipient and

questionnaires for the caregiver. For each target population two versions exist, one for baseline (baseline measurement), the second for follow-up measurement. The questionnaires are structured as follows:

- General questions: age, gender, socioeconomic status (= zip code), ethnicity, education level, marital status, living situation.
- Perceived health status (EQ-5D), utility (EQ-5D), multimorbidity (GGD monitor) and functioning (Katz-15).
- Well-being: psychological, social, quality of life (RAND-36 questions and Cantrill's Ladder).
- Service use: hospitalization (duration), unplanned GP care, home care, admission to a nursing home or home for the elderly, day care, and day treatment.

- SMAS-30: For evaluation of the effect of self-management support, the SMAS-30 is used. The SMAS-30 is based on the concept of self-management in relation to well-being. The questionnaire consists of 30 questions with five or six possible answer categories (Likert scale). Scores are summed and transformed to an overall scale score (range 0-100). A higher score indicates a higher self-management ability. The internal consistency is good (Cronbach's Alpha = .92).

  NB. For practical reasons, the SMAS-30 will be handed over to the older adults in the intervention group during the first visit by the case manager or social worker. The reason for this is practical in nature: the questionnaire is so extensive, that it might constitute an excessive burden for the older adults if it was included in the baseline questionnaire or history taking.
- PIH scale: the Partners In Health scale (PIH) measures the self-management knowledge and behavior of the older adults in relation to aging and the chronic conditions that he or she suffers from. This questionnaire, which consists of 12 items, was validated in 2010 [29]. This questionnaire is related to the elements of the Chronic Care Model, but in contrast to the PACIC it does not relate to the perceived quality of care as provided by the professionals, but by the older adults. [30]

6.1.3 Caregivers – primary outcome

Primary outcome is caregiver burden, measured with the Caregiver Strain Index (CSI).

- CSI: The Caregiver Strain Index (CSI) measures the burden experienced by the caregiver. The CSI is a widely used instrument to measure caregiver burden [25] and has good reliability and construct validity. [26] It consists of thirteen propositions that are answered with "yes" or "no." If seven or more items are answered with “yes,” the caregiver might be strained. The questionnaire covers five areas: employment, financial, physical, social, and leisure activities. [26]

6.1.4 Caregivers – Secondary outcomes

Secondary outcomes are the caregivers' load measured with the Self-rated Burden Visual Analogue Scale (Self-rated Burden VAS) and the Caregivers Quality of Life (Caregiver QOL), perceived health (RAND-36), and perceived quality of life (RAND-36 and Cantrill's Ladder). These secondary outcomes are all part of the MDS caregiver version.

- <u>MDS caregiver – combination of questionnaires</u>:
  - General questions: age, sex, socioeconomic status (= zip code), age caretaker, caretaker gender, socioeconomic status caretaker, to care recipient relationship, living together with caretaker himself.
  - Perceived health (RAND-36 questions)
  - Caregiver burden: objective (iBMG instrument) and subjective (self-rated VAS and Caregiver Burden QOL).
  - Perceived quality of life (RAND-36 and Cantrill's Ladder).

6.1.5 Quality of care – primary outcome

Primary outcome of quality of care is the degree of complexity of care needs of the older adults (INTERMED score measured by the triage instrument) (see §6.1.1).

6.1.6 Quality of care – Secondary outcomes

Secondary outcome is the extent to which goals in the care plan have been reached (Goal Attainment) and older adults' experiences and satisfaction with the received care (Patient Assessment of Chronic Illness Care [PACIC]).

- <u>Goal Attainment:</u> The method Goal Attainment (Goal Attainment Scaling [23]) proved to be an effective method for measuring change, especially in complex health situations. [24] Goals are formulated in the individual care plans and checked at the end of the intervention period to what extent these goals have been met. The procedure is as follows: after the case manager or social worker, together with the older adult formulated a health problem, they formulate a goal, as concrete and feasible from the perspective of the older adults as possible. Subsequently, actions aimed at achieving this goal are taken. This is described in the care plan. During the evaluation of the care plan, the older adult and case manager register to what extent goals are achieved. Answer categories for each objective are: 0 = not achieved; 1 = partially achieved; 2 = largely achieved; 3 = achieved. When at the end, the health problem was worse than in the beginning, negative scores are used: -1 = not achieved and somewhat worsened; -2 = worsened; -3 = deteriorated. For each care plan, scores are summed into a total score and converted:
  - Highest achievable scores = number of targets x maximum possible score
  - Achieved score = number of goals achieved x total score
  - Percentage goals achieved = (achieved score / maximum possible score) x 100%.

The higher the percentage of goals achieved, the better the quality of care.

- PACIC: The PACIC evaluates specific actions or elements of quality of care, which are in line with (parts of) the Chronic Care Model, and which the older adults experienced during the intervention period. The questionnaire includes 20 items and was validated in 2005. [27] In a recent study, Vrijhoef et al. stated that the PACIC was the best tool for measuring client experiences with integrated care. [28]

6.1.7 Service use and cost

Part of the assessment of the impact of the new model of care *Embrace* is the evaluation of costs and benefits. This study will be performed by a health economist at the Department of Health Technology Assessment (HTA) of the UMCG, using state of the art methods and resources.
The overall difference in costs (incremental costs) between the intervention and care as usual, will be estimated (including 95% Confidence interval) from a societal perspective. Using the primary outcome measures (complex care needs) the ratio between costs and benefits will be presented. Besides the calculation of the incremental cost, the EQ - 5D (from the MDS) will be used to estimate the effect of in terms of cost per Quality Adjusted Life Year (QALY).

In this experiment, we have planned to perform short-term analysis with a time horizon of one year. Should this prove to be insufficient to make an informed decision regarding implementation, additional analysis with extrapolation of the data can be performed. Bootstrapping will be used for the evaluation of the uncertainty regarding the point estimates of the Incremental Cost-Effectiveness Ratios (ICER), based on the observed data. Using a cost effectiveness acceptability curve the degree of decision uncertainty will be presented, taking into account a growing willingness to pay for care for the elderly. Because of the short time horizon, discounting will not be applied.

- Care plans: To evaluate the care intensity of the older adults receiving care- and case management, the case managers will register the time they invested during the intervention period. For the older adults in the self-management group, the social worker will register the following: time invested on individual level, participation of an older adult in group activities focused on self-management support and prevention, consultation with the case manager (frequency and incidental or structural consultation), and referrals to i.e. occupational therapy, municipalities (domestic help and aids).
- QALY: The Quality Adjusted Life Year is determined by combining data on health care utilization and costs with qualitative data, in this case the perceived health status (EQ-5D) from the MDS. The QALY is based on the number of (additional) life years as a result of the intervention, the related service use and costs, corrected by the perceived health status (index score). Depending on the extent and severity of the limitation (s), this index score is between 0.0 and 1.0.

6.2 Randomization, blinding and treatment allocation *(page 29)*

After inclusion (per general practitioner practice), the older adults will be stratified into two strata of complexity of care needs, based on the data measured with the triage-instrument (complexity of care needs) (Figure 3). Older adults with an INTERMED score of 20 or higher (complex care needs) are eligible for care- /case management. Older adults with an INTERMED score below 20 (no complex care needs) are eligible for self-management support and prevention. Within these strata, older adults will be allocated to the control group (receiving usual care) or the intervention group (receiving care and support according to the new model), using balanced randomization techniques.

The software used for this random assignment to care as usual or the intervention groups also results in an equal distribution of prognostic and confounding characteristics. [31-33] The balancing variables are, among others based on the findings of the pilot Case management for older adults with complex care needs: receiving home help and/or home care. Added variables are: age, gender, education, housing situation, partner, caregiver, children, assistance in completing the questionnaires, and chronic conditions.

NB. Randomization at the level of GP practices was considered. However, as this is a transitional experiment, we have chosen to randomize within GP practices to be able to include a larger number of practices. The risk of treatment overflow was estimated to be low: older adults in the pilot project reported that they visited their general practitioner not often, and if they did, the average consultation time was short (6-10 minutes) and only aimed at resolving the actual problem.

8 STATISTICAL ANALYSIS *(page 35)*

8.1 Descriptive statistics

The characteristics of the older adults and caregivers at different time points will be described using descriptive statistics.

8.2 Univariate analysis

The relation between discrete variables will be tested with the Chi-square test (or Fisher exact, if applicable). Continuous variables will be evaluated, depending on the normality of their distributions, using parametric tests, such as the T-test and ANOVA, or using non-parametric test, such as the Mann-Whitney U test. Ordinal variables will be evaluated using the Wilcoxon rank-sum test. Differences will be assessed between groups and within groups.

8.3 Multivariate analysis

The effects of the intervention will be assessed using logistic (ordinal variables) and hierarchical (continuous variables) regression analysis. The clinical relevance of differences between intervention and control groups will be estimated by Cohen's effect sizes.
