# Supplementary material for: Effects of a population-based, person-centred and integrated care service on health, wellbeing and self-management of community-living older adults: A randomised controlled trial on Embrace
Source: PLoS One. 2018 Jan 19;13(1):e0190751. doi: 10.1371/journal.pone.0190751 (PMC5774687; doi:10.1371/journal.pone.0190751)
Supplement: S2 File — (PDF) [file pone.0190751.s002.pdf]

Concept

ONDERZOEKSPROTOCOL

SamenOud

Herontwerp van het zorgaanbod: naar vraaggerichte, samenhangende, proactieve en preventieve zorg en begeleiding voor ouderen.

April 2011

Protocoltitel: SamenOud

Herontwerp van het zorgaanbod: naar vraaggerichte, samenhangende, proactieve en preventieve zorg en begeleiding voor ouderen.

Verkorte titel	*SamenOud*
Versie	*1*
Datum	*April 2011*
Coördinerend onderzoeker / projectleider	*Prof. Dr. S.A. Reijneveld*<br>*Prof. Dr. H.P.H. Kremer*
Hoofdonderzoeker/uitvoerder	*Dr. K. Wynia*<br>*Onderzoeker Integrated Care*<br>*Gezondheidswetenschappen UMCG*<br>*k.wynia@med.umcg.nl*<br>*050-3632005*
Verrichter/opdrachtgever	*ZonMW*<br>*Postbus 93245*<br>*2509 AE Den Haag*
Onafhankelijk arts	*Prof. Dr. O.F. Brouwer*<br>*Afdeling Neurologie, UMCG*

PROTOCOL HANDTEKENINGEN

Naam	Handtekening	Datum
Afdelingshoofd:<br>*Prof. Dr. S.A. Reijneveld*<br>*Hoofd afdeling Gezondheidswetenschappen*		20-04-11
Coördinerend onderzoeker / projectleider:<br>*Dr. K. Wynia*<br>*Onderzoeker Integrated Care*		19-04-11

INHOUD

LIJST MET AFKORTINGEN ... 6
BEGRIPPENLIJST ... 6
SAMENVATTING ... 10
1. INTRODUCTIE EN RELEVANTIE ... 12
1.1 Relevantie ... 15
1.1.1 Casemanagement project ... 15
1.1.2 Relevantie voor betrokken partijen ... 16
2. DOELSTELLINGEN ... 18
3. ONDERZOEKSDESIGN ... 20
3.1 Onderzoeksdesign ... 20
3.2 Duur onderzoek ... 20
3.3 Setting ... 20
4. STUDIEPOPULATIE ... 21
4.1 Populatie ... 21
4.2 Inclusiecriteria ... 21
4.3 Exclusiecriteria ... 21
4.4 Steekproefomvang ... 22
5. BEHANDELING VAN PROEFPERSONEN ... 24
5.1 Theoretische inbedding ... 24
5.1.1 Het Chronic Care Model (CCM) ... 24
5.1.2 De Kaiser Permanente (KP) Triangle ... 24
5.2 Interventie ... 24
5.2.1 Onderdeel 1: Organisatie van zorg – Ouderenzorg Team (OT) ... 26
5.2.2 Onderdeel 2: Organisatie van zorg – Care- en casemanagement ... 26
5.2.3 Onderdeel 3: Zelfmanagementsupport en preventie ... 29
5.3 Gebruikelijke zorg ... 32
6. METHODE ... 33
6.1 Uitkomstmaten en meetinstrumenten ... 33
6.1.1 Oudere – Primaire uitkomstmaat ... 33
6.1.2 Oudere – Secundaire uitkomstmaten ... 33
6.1.3 Mantelzorger – Primaire uitkomstmaat ... 35
6.1.4 Mantelzorger – Secundaire uitkomstmaten ... 35
6.1.5 Kwaliteit van zorg – Primaire uitkomstmaat ... 35
6.1.6 Kwaliteit van zorg – Secundaire uitkomstmaten ... 35
6.1.7 Zorggebruik en kosten – Primaire uitkomstmaat ... 36
6.2 Randomisatie, blinding and treatment allocation ... 40
6.3 Studieprocedures ... 42
6.4.1 Dataverzameling T0 ... 42
6.4.2 Dataverzameling T1 ... 44
6.4.3 Dataverzameling $T_{variabel}$ ... 46
6.4.4 Interventie ... 46

6.5 Terugtrekken uit de studie 46
6.5.1 Specifieke criteria voor terugtrekken uit de studie 47
6.6 Vervanging van proefpersonen na terugtrekken uit studie 47
6.7 Follow-up van proefpersonen die uit de studie zijn teruggetrokken 47
6.8 Voortijdige beëindiging van de studie 47
7 VEILIGHEID 48
7.1 Sectie 10 WMO 48
7.2 Ernstige voorvallen 48
7.3 Follow-up van ernstige voorvallen 48
8 STATISTISCHE ANALYSE 49
8.1 Beschrijvende statistiek 49
8.2 Univariate analyses 49
8.3 Multivariate analyses 49
9 ETHISCHE ASPECTEN 50
9.1 Reglementen 50
9.2 Werving en toestemming 50
9.2.1 Ouderen 50
9.2.2 Mantelzorgers 51
9.3 Bezwaar door wilsonbekwame personen 51
9.4 Voordelen en nadelen voor de respondenten (ouderen en mantelzorgers) 51
10 ADMINISTRATIEVE ASPECTEN EN PUBLICATIE 53
10.1 Verwerking en opslag van data en documenten 53
10.2 Amendementen 53
10.3 Jaarlijks voortgangsverslag 53
10.4 Eindverslag van de studie 53
11 REFERENTIES 55

LIJST MET AFKORTINGEN

CCM	*Chronic Care Model*
CSI	*Caregiver Strain Index*
EOD	*Elektronisch Ouderen Dossier*
GFI	*Groninger Frailty Indicator*
KP Triangle	*Kaiser Permanente Triangle*
MDS	*Minimale Data Set*
NFU	*Nederlandse Federatie van Universitaire Medische Centra*
NPO	*Nationaal Programma Ouderenzorg*
OT	*Ouderenzorg Team*
PGO	*Programma Geïntegreerde Ouderenzorg*
QOL	*Quality of Life (kwaliteit van leven)*
RCT	*Randomised Controlled Trial*
VAS	*Visueel Analoge Schaal*
WMO	*Wet Maatschappelijke Ondersteuning of: Wet Medisch-wetenschappelijk Onderzoek met mensen, afhankelijk van de context.*

BEGRIPPENLIJST

Caremanagement	*Kortdurende intensieve zorg en begeleiding door de casemanager.*
Casemanagement	*Langdurige intensieve zorg en begeleiding door de casemanager.*
Casemanager	*Een gespecialiseerd verpleegkundige of wijkverpleegkundige die care- en/of casemanagement verleent aan ouderen met complexe zorgbehoeften.*
Chronic Care Model (CCM)	*Internationaal kwaliteitsmodel dat werkt met interventies op zes componenten die bijdragen aan betere zorg voor chronisch zieken, zoals interventies op het gebied van de organisatie van de zorg, zelfmanagement, informatiesystemen en evidence-based werken; uitgangspunt is een productieve interactie tussen geïnformeerde, actief participerende patiënten en een voorbereid en proactief werkend team van beroepskrachten.*
Complexiteit van zorgbehoeften	*Biopsychosociale complexiteit van (zorg)behoeften, te meten in vier domeinen: biologische, psychologische en sociale behoeften en het zorgsysteem, in het tijdsperspectief van verleden, heden en toekomst. Wordt gemeten met de INTERMED.*
Controlegroep	*Deelnemende ouderen die de gebruikelijke zorg ontvangen.*
Elektronisch Ouderen Dossier (EOD)	*Een elektronisch dossier met daarin gegevens verzameld met het triage-instrument, gegevens uit de anamnese, het zorgleefplan en een voortgangsrapportage van ouderen. Het EOD is in te zien door de leden van het OT en de onderzoekers.*
EQ-5D	*Een gestandaardiseerd self-report meetinstrument om gezondheidsuitkomsten te meten.*
Gebruikelijke zorg	*Zorg gebaseerd op het huidige zorgmodel: zorg en begeleiding van de huisarts en andere hulpverleners en hulpverlenende instanties.*

Goal Attainment Scaling	*Methode om doelgerichte activiteiten op kwantitatieve wijze te evalueren.*
GRIP- en GLANS-cursus	*Cursussen die zich richten op het versterken van de eigen regie en het eigen welbevinden van (kwetsbare) oudere mensen.*
Informed consent	*Vrijwillig verkregen toestemming van een persoon of een verantwoordelijke voor deelname aan het onderzoek op grond van informatie.*
Interventie	*Het nieuwe zorgmodel SamenOud dat gebaseerd is op het Chronic Care Model en de Kaiser Permanente Triangle.*
Interventiegroep	*Deelnemende ouderen die de interventie ontvangen.*
Interventiestudie	*Experimenteel onderzoek waarbij het effect van een experimentele interventie wordt onderzocht, in dit geval het nieuwe zorgmodel SamenOud.*
Kaiser Permanente (KP) Triangle	*Een proactieve benadering om de zorg voor chronisch zieken te organiseren. De KP Triangle onderscheidt drie groepen chronische zieken of ouderen, gebaseerd op de complexiteit van de zorgbehoeften. De intensiteit en duur van de begeleiding door een team wordt afgestemd op de mate van complexiteit van de zorgbehoeften van de patiënt.*
Kwetsbaarheid	*Een wankel evenwicht dat gemakkelijk verstoord wordt door lichamelijke, psychische of sociale oorzaken. Wordt gemeten met de Groninger Frailty Indicator (GFI).*
Loss-to-follow-up	*Uitval van deelnemers gedurende het onderzoek.*
Minimale Data Set	*Een vragenlijst die door het Nationaal Programma Ouderenzorg (NPO) is opgesteld om te kunnen vaststellen of de zorg voor ouderen verbeterd is, en om de uitkomsten van de uitkomsten van de verschillende regio's onderling te vergelijken. NPO-projecten zijn verplicht deze vragenlijsten te gebruiken. Er zijn twee versies (basismeting en vervolgmeting) voor ouderen en twee versies voor de mantelzorger.*
Nationaal Programma Ouderenzorg (NPO)	*Een programma van ZonMw, in samenwerking met de Nederlandse Federatie van Universitair medische centra (NFU) en in opdracht van het ministerie van Volksgezondheid, Welzijn en Sport. Doel van het programma is om een samenhangend zorgaanbod te creëren dat beter is afgestemd op de behoeften van ouderen.*
Onderzoek	*In dit protocol: het testen van de effecten van het nieuwe zorgmodel in een interventiestudie door middel van een Randomised Controlled Trial.*
Ouderenadviseur	*Een HBO-opgeleide medewerker van een deelnemende welzijnsorganisatie, die zelfmanagementsupport en preventie verleent aan kwetsbare ouderen en ouderen adviseert en begeleidt m.b.t. regelingen, voorzieningen en mogelijkheden op het gebied van wonen, zorg, welzijn en financiën.*
Ouderenzorg Team (OT)	*Een team dat, onder leiding van de huisarts en verder bestaande uit een specialist ouderengeneeskunde, casemanager en ouderenadviseur, zorg verleent volgens het nieuwe zorgmodel SamenOud.*
Preventie	*Alle activiteiten gericht op het voorkomen of het beperken van negatieve gevolgen of gebeurtenissen als gevolg van het ouder worden, zoals beperking in zelfredzaamheid, mobiliteit buitenshuis, afname sociale participatie, tekort aan voeding en vocht, veiligheid in huis (valincidenten), medische overbehandeling, etc.*

Programma Geïntegreerde Ouderenzorg (PGO)	*Het transitie-experiment dat het nieuwe zorgmodel SamenOud (de interventie) ontwikkelt, realiseert en onderzoekt.*
SamenOud	*Het nieuwe zorgmodel dat gebaseerd is op alle onderdelen van het CCM en de KP Triangle en door het PGO ontwikkeld, gerealiseerd en onderzocht wordt in drie gemeenten in Oost-Groningen.*
Transitie-experiment	*Volgens de omschrijving van het NPO: een project waarbij een regionaal netwerk een experiment bedenkt dat de samenhang, kwaliteit en betaalbaarheid van de zorg moet verbeteren.*<br>*Het transitie-experiment PGO ontwikkelt, realiseert en onderzoekt de effecten van het nieuwe zorgmodel SamenOud in drie gemeenten in Oost-Groningen.*
Triage-instrument	*Meetinstrument dat de complexiteit van zorgbehoeften (INTERMED), kwetsbaarheid (GFI) en welbevinden (Welbevindenlijst) meet.*
Welbevinden	*Gemeten met de Welbevindenlijst. Deze lijst is onderdeel van het triage-instrument en stelt de zaken vast die mensen belangrijk vinden in het dagelijks leven en de mate waarin men tevreden zijn op die gebieden.*
Zelfmanagement	*Het zodanig omgaan met de gevolgen van het ouder worden (symptomen, behandeling, lichamelijke en sociale consequenties en bijbehorende aanpassingen in leefstijl) dat de gevolgen van de aandoening (of het ouder worden) optimaal worden ingepast in het leven (gebaseerd op de definitie van NPCF-CBO).*
Zelfmanagementsupport	*Ondersteuning van het zelfmanagementvermogen van ouderen, verleend door het OT, om adequaat te kunnen reageren op de gevolgen van ziekte en beperkingen als gevolg van het ouder worden.*
Zorgleefplan	*Een plan dat is opgesteld door de oudere en het OT, met (potentiële) gezondheidsproblemen, wenselijke doelsituatie en afspraken over zorg en ondersteuning. Het zorgleefplan heeft betrekking op de vier domeinen van kwaliteit van leven: mentaal welbevinden, lichamelijk welbevinden en gezondheid, participatie en woon- en leefomstandigheden.*
Zorgmodel	*De wijze waarop de zorg georganiseerd is.*

SAMENVATTING

Achtergrond:
Het aantal 65-plussers in Nederland stijgt. Dit wordt o.a. veroorzaakt door de stijging van de gemiddelde levensverwachting. Terwijl de levensverwachting stijgt, neemt het gemiddeld aantal jaren in goede gezondheid en zonder lichamelijke beperkingen, nagenoeg niet toe. Bijna een kwart van de 75-plussers heeft vier of meer chronische aandoeningen. Vooral na het 75^{e} levensjaar stijgt het aantal mensen met complexe zorgbehoeften dat ondersteuning nodig heeft op meerdere gebieden, en neemt ook het beroep op professionele ondersteuning toe. Ons huidige zorgaanbod is vooral reactief ingericht voor het oplossen van acute en kortdurende gezondheidsproblemen, en is (nog) niet in staat een goed antwoord te geven op langdurige complexe zorgbehoeften van ouderen. Een mogelijke oplossing biedt het Chronic Care Model (CCM). Het CCM heeft tot doel samenhangende, proactieve en preventieve zorg te ontwikkelen voor chronisch zieken en ouderen. Het is een multidimensionaal model dat leidt tot een herontwerp van het zorgaanbod gericht op mensen met langdurige aandoeningen en beperkingen. In dit transitie-experiment worden de elementen van het CCM toegepast in combinatie met de zgn. Kaiser Permanente (KP) Triangle, die uitgaat van drie te onderscheiden groepen chronische zieken of ouderen, gebaseerd op de complexiteit van de zorgbehoeften.
Belangrijkste doelstelling van dit transitie-experiment is het realiseren van een nieuw zorgmodel, *SamenOud* genoemd. *SamenOud* realiseert vraaggerichte, samenhangende, proactieve en preventieve zorg en begeleiding voor thuiswonende ouderen van 75 jaar en ouder, waardoor de complexiteit van de zorgbehoeften van deze ouderen afneemt, het welbevinden toeneemt en de (overall) zorgkosten afnemen of tenminste gelijk blijven.
Methode/design:
In dit transitie-experiment ontvangen alle 75-plussers van vijftien huisartsenpraktijken in Stadskanaal, Veendam en Pekela zorg en begeleiding van een Ouderenzorg Team. Dit team, onder leiding van een huisarts, bestaat uit een specialist ouderengeneeskunde, een casemanager (gespecialiseerd verpleegkundige of wijkverpleegkundige) en een ouderenadviseur van een welzijnsorganisatie.
De intensiteit en duur van de begeleiding van de oudere is afhankelijk van de complexiteit van de zorgbehoeften en de kwetsbaarheid. Ouderen met complexe zorgbehoeften ontvangen intensieve zorg en begeleiding van de casemanager. De groep zonder complexe zorgbehoeften ontvangt zelfmanagement support, verleend door de ouderenadviseur, waarbij ouderen die verhoogd kwetsbaar zijn individuele begeleiding ontvangen.
De complexiteit van de zorgbehoeften en mate van kwetsbaarheid van de ouderen wordt vastgesteld met een jaarlijkse screening. Bij ouderen met complexe zorgbehoeften of risico daarop (verhoogd kwetsbaar) wordt een anamnese afgenomen met aandacht voor wonen, welzijn en zorg. In overleg met de ouderen en het Ouderenzorg Team of de ouderenadviseur wordt een individueel zorgleefplan opgesteld. Dit zorgleefplan wordt in samenwerking met een uitgebreid netwerk van hulpverleners en organisaties gerealiseerd.
De vraagstelling van het onderzoek in het transitie-experiment is: Wat zijn de effecten van het nieuwe zorgmodel *SamenOud* op de complexiteit van de zorgbehoeften, de kwetsbaarheid en het welbevinden van ouderen, het zelfmanagementvermogen en de zelfredzaamheid van de ouderen, de belasting van de mantelzorgers, de kwaliteit van zorg, het zorggebruik en de kosten? Een Randomised Controlled Trial zal inzicht geven in de antwoorden op deze vragen.

1. INTRODUCTIE EN RELEVANTIE

Het aantal 65-plussers in Nederland is de afgelopen decennia gestegen van nog geen 800.000 in 1950 tot bijna 2,4 miljoen in 2007 (www.cbs.nl). Dit aantal zal blijven stijgen en wordt onder andere veroorzaakt door de stijging van de gemiddelde levensverwachting. In 2003 was de levensverwachting bij geboorte voor mannen 76,3 jaar en voor vrouwen 80,9 jaar. Hoewel de levensverwachting stijgt, neemt het gemiddeld aantal jaren dat mannen en vrouwen kunnen verwachten in goede gezondheid te blijven nagenoeg gelijk. De levensverwachting zonder lichamelijke beperkingen is nu 69,9 jaar voor mannen en 69,8 jaar voor vrouwen (www.rivm.nl). Bijna een kwart van de 75-plussers heeft vier of meer chronische aandoeningen (www.cbs.nl). Ongeveer negen op de tien 75-plussers is alleenstaand. Het is dan ook niet verwonderlijk dat vooral na het 75e levensjaar het aantal mensen met complexe zorgbehoeften dat ondersteuning nodig heeft op meerdere gebieden stijgt en ook het beroep op professionele ondersteuning toeneemt. [1]

Het huidige zorgaanbod in Nederland is vooral reactief ingericht voor het oplossen van acute en kortdurende gezondheidsproblemen en is (nog) niet in staat een goed antwoord te geven op de complexe zorgbehoeften van onze kwetsbare ouderen. Ter illustratie: een recent verschenen rapport van het NIVEL rapporteert: "*Van de mensen boven de 75 jaar moet 66% naar meerdere loketten voor ondersteuning*". [1] Voor de oudere neemt – naast het organiseren van de ondersteuning bij de toenemende beperkingen – ook het aantal te volgen behandelingen toe als gevolg van de multimorbiditeit. Ouderen moeten dit vaak zelf weten te organiseren en op elkaar af te stemmen.

Een mogelijke oplossing voor het anders organiseren van het zorgaanbod voor ouderen met complexe zorgbehoeften biedt het Chronic Care Model (CCM) (www.improvingchroniccare.org). Het CCM (Figuur 1) heeft tot doel samenhangende, proactieve en preventieve zorg te ontwikkelen voor chronisch zieken en ouderen. Het CCM is een multidimensionaal model dat leidt tot een herontwerp van het zorgaanbod, gericht op mensen met langdurige aandoeningen en beperkingen. [2-4] Het ultieme doel van het CCM is een goed geïnformeerde en geactiveerde patiënt die in interactie is met een voorbereid en proactief team, waarbij het proces van anamnese, zelfmanagement support, optimaliseren van zorg en behandeling en follow-up resulteert in hoogwaardige en bevredigende resultaten.

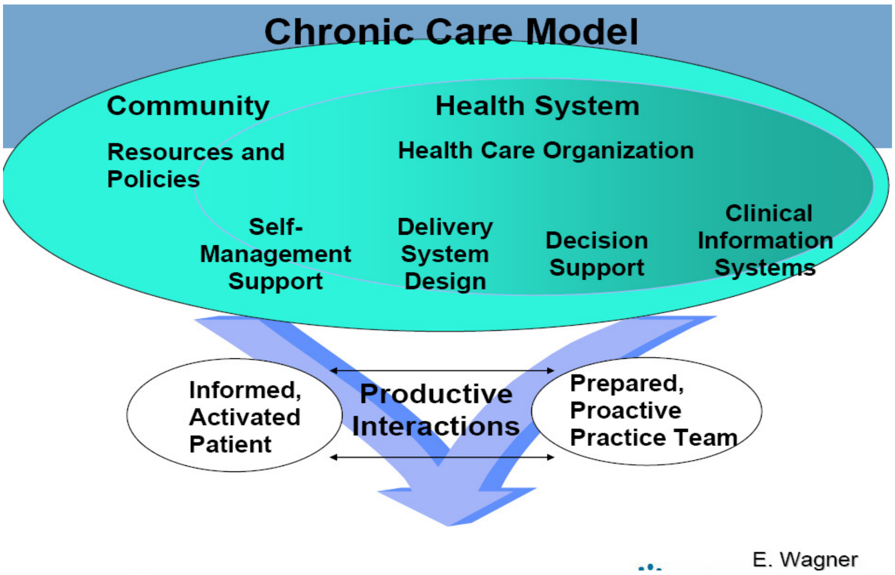

Figuur 1 *Het Chronic Care Model*

Het CCM omvat vier, met elkaar samenhangende en inter-acterende, basisonderdelen:

1) *Zelfmanagementsupport*: Samen met de oudere en hun naasten worden vaardigheden en zelfvertrouwen bevorderd om adequaat te kunnen reageren op de gevolgen van ziekte en beperkingen.
2) *Delivery system design*: Het ontwerp van het zorgverleningsysteem door het creëren van teams van medische en niet-medische teamleden. Het team wordt aangestuurd door een medicus, verleent acute zorg en coördinatie van geplande chronische zorg, en ondersteunt het zelfmanagementvermogen van de patiënt. Monitoring en geplande contacten met de patiënt zijn belangrijke voorwaarden.
3) *Ondersteuning in besluitvorming*: Richtlijnen en zorgpaden bieden standaarden voor optimale zorg en moeten een plaats krijgen in de dagelijkse werkwijze.
4) *Klinische informatiesystemen*: Elektronische informatie biedt belangrijke ondersteuning bij het werken met standaarden en richtlijnen, bij rapportage binnen het team, en bij registratie van de planning van de individuele patiëntenzorg.

Deze vier basisonderdelen zijn geplaatst in de context van de maatschappelijke omgeving, de zorgaanbieders in de eerste- en tweedelijn, het gezondheidszorgsysteem en het verzekeringsstelsel. De huisarts heeft een centrale positie in het CCM, omdat langdurige zorgverlening vooral in de eerstelijn plaatsvindt en de huisarts veelal het centrale aanspreekpunt is.

Het CCM model wordt ook toegepast in combinatie met de zgn. Kaiser Permanente (KP) Triangle (Figuur 2). De KP Triangle gaat uit van drie te onderscheiden groepen chronische zieken of ouderen, gebaseerd op de complexiteit van de zorgbehoeften. [4, 5] De intensiteit en duur van de begeleiding door een team wordt afgestemd op de mate van complexiteit van de zorgbehoeften van de chronische zieke.

In het algemeen heeft de grootste groep chronisch zieken (±80%) geen complexe zorgbehoeften en ontvangt vooral *zelfmanagementsupport*. De tussengroep (±15%) heeft complexe zorgbehoeften die op te lossen zijn door kortdurende intensieve begeleiding, ook wel *caremanagement* genoemd. De kleinste groep chronisch zieken (±5%) heeft complexe zorgproblemen die langdurige monitoring en begeleiding noodzakelijk maken o.a. om opnames te voorkomen. Dit wordt *casemanagement* genoemd (https://www.kaiserpermanente.org/).

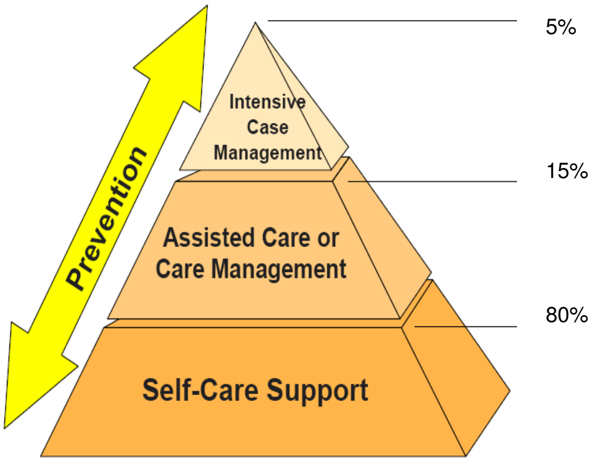

Figuur 2 *Kaiser Permanente Triangle*

Het beoogde resultaat van dit transitie-experiment is een nieuw zorgmodel, *SamenOud* genoemd, dat alle onderdelen van het CCM in onderlinge samenhang en in combinatie met de KP Triangle realiseert voor ouderen van 75 jaar en ouder in vijftien huisartspraktijken in Stadskanaal, Veendam en Pekela in Oost-Groningen.

1.1 Relevantie

1.1.1 Casemanagement project

Het nieuw zorgmodel *SamenOud* is relevant voor ouderen omdat het een vraaggerichte, samenhangende, proactieve en preventieve oplossing biedt, die tegemoet komt aan (het voorkomen van) complexe zorgbehoeften. De resultaten van de pilot '*Casemanagement voor ouderen met complexe zorgbehoeften*' zijn een duidelijke indicatie voor de potentiële effecten van *SamenOud*. [6] De pilot Casemanagement vond in 2009 plaats in twee huisartspraktijken in Stadskanaal. Twintig ouderen (75 jaar en ouder) met complexe zorgbehoeften ontvingen zes maanden intensieve begeleiding van een casemanager (ervaren wijkverpleegkundige), huisarts en specialist ouderengeneeskunde (het kernteam). Het team werkte nauw samen met o.a. thuiszorg, welzijnsorganisatie, gemeente en paramedische diensten om te komen tot een integratie van het zorgaanbod op de terreinen wonen, welzijn en zorg. Doelstellingen waren het bevorderen van het welbevinden van ouderen en het voorkomen van opnames. Resultaten lieten zien dat na zes maanden de complexe zorgbehoeften bij dertien ouderen waren opgelost en bij zeven ouderen gelijk bleven. Bij zestien ouderen (80%) werd de ergotherapeut ingeschakeld en werden, in samenwerking met de gemeente, aanpassingen in huis en de aanschaf van hulpmiddelen gerealiseerd. Hierdoor nam de zelfredzaamheid, de veiligheid in huis (valpreventie) en mobiliteit buitenshuis toe. Regelmatig werd een welzijnsorganisatie ingeschakeld voor ondersteuning van de mantelzorg, hulp bij het invullen van formulieren, etc. Uit interviews bleek dat een aantal ouderen actiever in contacten naar anderen werd en ook beter de zorgbehoeften kon formuleren. [6]

1.1.2 Relevantie voor betrokken partijen

Het nieuwe zorgmodel *SamenOud* is relevant voor ouderen omdat het doel van dit zorgmodel is dat zij beter passende zorg en begeleiding ontvangen waardoor, uiteindelijk, het welbevinden kan toenemen.
Het nieuwe zorgmodel *SamenOud* is relevant voor huisartsen, specialisten ouderengeneeskunde, wijkverpleegkundigen en ouderenadviseurs omdat ze op gestructureerde wijze begeleid worden door de projectleiders van het transitie-experiment in het vorm geven van een multidisciplinair team (Ouderenzorg Team (OT)) en het samenwerken met maatschappelijke organisaties en zorgaanbieders in eerste- en tweedelijn.
SamenOud is relevant voor:

1) De huisarts omdat hij/zij leiding gaat geven aan een multidisciplinair team en meer zicht krijgt op en invloed heeft op de kwetsbaarheid en complexe zorgbehoeften van ouderen in de eigen huisartspraktijk.
2) De specialist ouderengeneeskunde omdat hij/zij nu ook buiten de verpleeghuizen specialistische ouderenzorg kan toepassen.
3) De wijkverpleegkundige omdat de rol van care- en casemanager recht doet aan het generalistische karakter van het beroep en een aantrekkelijk carrièreperspectief biedt.
4) De ouderenadviseur, de welzijnsorganisatie en gemeenten omdat *SamenOud* de mogelijkheid biedt samen met het OT haar activiteiten en mogelijkheden, effectiever en doelgerichter in te zetten.

De keuze voor een ingewikkelde en ingrijpende operatie als het invoeren van een nieuw zorgmodel als *SamenOud* is relevant en noodzakelijk: het huidige reactieve en op behandeling gerichte zorgmodel heeft beperkingen en biedt te weinig ruimte om door middel van kleine aanpassingen te kunnen voldoen aan de eisen van langdurige, vraaggerichte, samenhangende, proactieve en preventieve zorg.
De keuze voor het CCM is relevant vanwege de gebleken populariteit en effectiviteit van het model. [5, 7-9] In de Verenigde Staten is het CCM in een groot aantal settings geïmplementeerd of heeft het geleid tot een herontwerp van het zorgaanbod. Buiten de Verenigde Staten is het CCM in een groot aantal landen (o.a. Australië, Canada, Engeland) van invloed geweest op het beleid voor chronisch zieken. Ook in Nederland wint het CCM aan

populariteit. De World Health Organization (WHO) heeft het CCM geadopteerd als basis voor wereldwijde toepassing. [5] De keuze voor het CCM in combinatie met de KP Triangle is relevant uit het oogpunt van kwaliteit van zorg en kosteneffectiviteit [8].

De pilot 'Casemanagement voor ouderen met complexe zorgbehoeften' liet zien dat samenhangende, proactieve en preventieve zorg ervoor kon zorgen dat de complexiteit van de zorgbehoeften significant afnam of voorkwam dat de complexiteit toenam. Het is daarom aannemelijk dat – op relatief eenvoudige wijze – spoedopnames en opnames voor langdurig verblijf voorkomen of uitgesteld kunnen worden. Dit zal uiteindelijk kosten besparen. Door aanpassingen in huis en het gebruik van hulpmiddelen zal de veiligheid in huis toenemen en zullen valincidenten afnemen. Ook dit zal een besparing in de kosten opleveren. In elk geval zal het onderzoek naar de effecten van *SamenOud* precieze informatie kunnen verschaffen over de samenhang tussen zorgbehoeften, prestatie-indicatoren en kosten.

Gedegen onderzoek (Randomised Controlled Trial) naar de effecten van het nieuwe zorgmodel *SamenOud* waarin alle onderdelen van het CCM gelijktijdig worden gerealiseerd is relevant. De resultaten van het onderzoek naar de effecten van het model op de complexiteit van zorgbehoeften, kwetsbaarheid, het welbevinden, de kwaliteit van zorg, het zorggebruik en de kosten kunnen een waardevolle bijdrage leveren aan verdere implementatie van *SamenOud*. Ook voor andere doelgroepen zoals chronisch zieken, jongeren, of mensen met overgewicht kunnen vergelijkbare programma's in de toekomst geïmplementeerd worden.

De keuze voor Oost-Groningen is relevant omdat hier de bevolking relatief meer vergrijst dan landelijk gezien (www.horizonline.nl). In 2010 was in de gemeente Stadskanaal 9,1% van de inwoners 75 jaar of ouder. Landelijk ligt dit percentage op 6,9%. De verwachting is dat in 2030 in Stadskanaal 13,1% van de inwoners 75 jaar of ouder is (vs. 11,5% landelijk) en zal stijgen met 42% van 3.051 in 2010 tot 4.321 in 2030. Omringende gemeentes laten hetzelfde beeld zien.

2. DOELSTELLINGEN

Belangrijkste doelstelling van het nieuwe zorgmodel *SamenOud* is het realiseren van vraaggerichte, samenhangende, proactieve en preventieve zorg waardoor de complexiteit van de zorgbehoeften van ouderen afneemt, het welbevinden toeneemt en de (overall) zorgkosten afnemen of minimaal gelijk blijven.
Hiervoor wordt een herontwerp van het zorgaanbod gerealiseerd dichtbij de oudere thuis: in de eigen huisartspraktijk. Alle basisonderdelen van het Chronic Care Model (CCM) worden in onderlinge samenhang gerealiseerd. Een Ouderenzorg Team (OT) – onder leiding van de huisarts – is de basis van waaruit zorg en begeleiding aan alle 75-plussers in de huisartspraktijk verleend en gecoördineerd wordt. Uitgangspunt is dat de oudere de vraag bepaalt en kiest uit het aanbod. De intensiteit en duur van de begeleiding door het OT wordt afgestemd op de complexiteit van de zorgbehoeften van de oudere. Uitgaande van de drie complexiteitsgroepen in de KP Triangle, ontvangen ouderen met complexe zorgbehoeften intensieve zorg en begeleiding van het OT (inclusief zelfmanagementsupport en preventie). Ouderen zonder complexe zorgbehoeften ontvangen zelfmanagementsupport en preventie van ouderenadviseurs die deel uitmaken van het OT, zodat een toename van de complexiteit van de zorgbehoeften tijdig gesignaleerd kan worden.

De belangrijkste beoogde resultaten van *SamenOud* zijn:

1) Vijftien goed opgeleide en goed werkende Ouderenzorg Teams (OTs), die gevestigd zijn in evenzoveel huisartspraktijken in drie gemeenten in Oost-Groningen.
2) De OTs beschikken over een sterk netwerk van gemeenten, zorgaanbieders (eerste- en tweedelijn) en zorgverzekeraars waarmee intensieve contacten bestaan.
3) De OTs hebben aan ouderen (75 jaar en ouder) in deze praktijken samenhangende, proactieve en preventieve zorg verleend.
4) Bij ouderen met hoog complexe zorgbehoeften is sprake van afname van complexiteit van de zorgbehoeften of wordt een opname in het ziekenhuis of verpleeghuis voorkomen of uitgesteld.
5) Bij ouderen zonder complexe zorgbehoeften wordt, voor zover mogelijk, voorkomen dat deze ontstaan.
6) Bij (een deel van) de ouderen is het zelfmanagementvermogen toegenomen.

Centrale vraagstelling van het onderzoek in het transitie-experiment is: *Wat zijn de effecten van het nieuwe zorgmodel SamenOud voor 75-plussers op:*

1) De complexiteit van de zorgbehoeften, de kwetsbaarheid en het welbevinden van de ouderen.
2) Het zelfmanagementvermogen en de zelfredzaamheid van de ouderen.
3) De belasting van hun mantelzorgers.
4) De kwaliteit van zorg.
5) Het zorggebruik en de kosten van de gezondheidszorg.

3. ONDERZOEKSDESIGN

3.1 Onderzoeksdesign

De centrale vraagstelling in het onderzoek wordt beantwoord met een pre-stratified Randomised Controlled Trial met balanced allocation.

3.2 Duur onderzoek

De interventieperiode in het onderzoek naar de effecten van het nieuwe zorgmodel *SamenOud* heeft een beoogde looptijd van 12 maanden en start 1 december 2011.

3.3 Setting

De interventie vindt plaats in Stadskanaal, Veendam en Pekela in Oost-Groningen. De huisartsenpraktijken in deze gemeenten zijn de basis van de Ouderenzorg Teams (OTs). De OTs werken nauw samen met de maatschappelijke en eerste- en tweedelijns gezondheidszorgorganisaties in en om deze gemeenten.

4. STUDIEPOPULATIE

4.1 Populatie

De studie richt zich primair op ouderen van 75 jaar en ouder die ingeschreven staan bij de vijftien deelnemende huisartspraktijken. Ook de eventuele mantelzorgers van de ouderen kunnen in aanmerking komen voor deelname.

- *Ouderen:* Alle ouderen van 75 jaar en ouder van een huisartsenpraktijk. Van ouderen in deze leeftijdscategorie is bekend dat vanaf deze leeftijd (het risico op) kwetsbaarheid en complexiteit van de zorgbehoeften sterk toenemen.
- *Mantelzorgers:* De belangrijkste mantelzorger van de oudere. Meestal is dit de partner van de oudere, maar het kan ook een andere naaste zijn. Een belangrijk deel van de zorg voor de oudere wordt verleend door de mantelzorger. Bekend is dat naarmate de zorglast toeneemt, de kans op overbelasting groter wordt. [10-11] Hiermee neemt ook de kans op opname in een instelling voor langdurig verblijf toe. Ondersteuning van de mantelzorg, dat een onderdeel is van de interventie, kan bijdragen aan uitstel of het voorkomen van een ongewenste en (relatief dure) langdurige opname.

4.2 Inclusiecriteria

- *Ouderen:* 75 jaar of ouder, thuiswonend of wonend in een verzorgingshuis en als patiënt ingeschreven bij één van de 15 deelnemende huisartspraktijken.
- *Mantelzorgers:* De oudere (zorgvrager) participeert in de interventiegroep in het onderzoek. NB. De belangrijkste mantelzorger is diegene die het grootste aandeel in de zorg heeft voor de oudere. Meestal is dit de partner, soms een naast familielid. Inclusie van de mantelzorgers in de controlegroep is overwogen. Besloten is deze doelgroep in de controlegroep niet te benaderen voor deelname aan het onderzoek. Dit vanwege de ingewikkelde identificatieprocedure van deze potentiële respondenten, die via de oudere met complexe zorgbehoeften (en via extra vragen in de vragenlijst) moet verlopen (zie ook blz. 30 Mantelzorgers).

  Mantelzorgers van ouderen met complexe zorgbehoeften of verhoogde kwetsbaarheid in de interventiegroep worden benaderd via de casemanager of de ouderenadviseur tijdens de anamnesegesprekken.

4.3 Exclusiecriteria

- *Ouderen:* Langdurig verblijf in een verpleeghuis, het ontvangen van een (andere) vorm van geïntegreerde zorg of het deelnemen aan een andere studie.
- *Mantelzorgers:* Het deelnemen aan een andere studie. Wanneer de belangrijkste mantelzorger de partner van de oudere is en hij/zij zelf ook complexe zorgbehoeften heeft, wordt deze mantelzorger geïncludeerd als ‘oudere’.

4.4 Steekproefomvang

De power voor deze studie is berekend voor het aantonen van verschillen tussen de interventie- en controlegroep in de gemiddelde score op de Visueel Analoge Schaal (VAS) van de EQ-5D [12] met een power van minstens 80% (beta van 20%). Uitgaande van een Intraclass Correlatie Coëfficiënt van 0.10 is een minimum aantal van 108 ouderen per groep nodig. Uitgaande van een aandeel van deze groep (die care- en casemanagement nodig heeft) van 20% van alle 75-plussers, een non-respons van 30% en loss-to-follow-up (overlijden of opname in verpleeghuis) van 30% moeten voor de inclusie van deze 108 ouderen in totaal 2202 ouderen benaderd worden:

- Uitgaande van twee groepen (interventie- en controlegroep) = 1101 ouderen benaderen per groep
- Respons van 70%: inclusie 771 ouderen per groep
- Loss-to-follow-up van 30%: 540 ouderen die in de analyse betrokken kunnen worden, per groep
- 20% in care- en casemanagement groep = 108 ouderen
- 80% in zelfmanagementsupport en preventie groep = 432 ouderen

Bij inclusie van 771 ouderen in het onderzoek en randomisatie naar de beide onderzoeksgroepen is de verwachtte verdeling van ouderen bij aanvang van de interventies (loss-to-follow-up buiten beschouwing gelaten) als volgt:

- 154 ouderen voor care- en casemanagement = 39 ouderen casemanagement en 115 ouderen caremanagement
- 617 ouderen voor zelfmanagementsupport = 123 ouderen individuele begeleiding en 493 ouderen groepsbegeleiding

5. BEHANDELING VAN PROEFPERSONEN

In dit hoofdstuk wordt eerst de theoretische inbedding van het nieuwe zorgmodel *SamenOud* geschetst (§5.1). Vervolgens wordt beschreven hoe de interventie eruit ziet voor de deelnemende ouderen (§5.2), en worden twee onderdelen van het Chronic Care Model (CCM) verder uitgewerkt (§5.2.1 – §5.2.3). Ten slotte wordt beschreven wat de gebruikelijke zorg inhoudt die de ouderen in de controlegroep ontvangen (§5.3).

5.1 Theoretische inbedding

5.1.1 Het Chronic Care Model (CCM)

Het nieuwe zorgmodel *SamenOud* is o.a. gebaseerd op het CCM voor het vormgeven van geïntegreerde zorg. [7, 9] (www.improvingchroniccare.org, zie ook Figuur 1, blz. 10). In het nieuwe zorgmodel worden de basisonderdelen van het CCM in onderlinge samenhang (verder) ontwikkeld en toegepast.

5.1.2 De Kaiser Permanente (KP) Triangle

De KP Triangle (Figuur 2, blz. 11) gaat uit van drie groepen chronische zieken of ouderen, gebaseerd op de complexiteit van de zorgbehoeften. [4,5] De intensiteit en duur van de begeleiding door het Ouderenzorg Team (OT) worden afgestemd op de mate van complexiteit van de zorgbehoeften van de oudere. Op basis van onderzoek [5, 8] en ervaringen uit de pilot ‘Casemanagement voor ouderen met complexe zorgbehoeften’ is de groep ouderen op basis van de complexiteit van de zorgbehoeften globaal in te delen in drie groepen, die elk een bepaalde mate van intensiteit in en duur van begeleiding nodig hebben (zie ook hoofdstuk 1, blz. 11):

I. De grootste groep ouderen (±80%) *zonder complexiteit* maar met – gezien de leeftijd – een verhoogd risico op toenemende afhankelijkheid en complexiteit van zorgbehoeften. Deze groep komt vooral in aanmerking voor zelfmanagementsupport en preventie.
II. Een middelgrote groep ouderen (±5%) met *kortdurend complexe* zorgbehoeften en verhoogd risico op een toename van complexiteit van zorgbehoeften. Deze groep komt in aanmerking voor caremanagement.
III. De kleinste groep ouderen (±5%) met *langdurig complexe* zorgbehoeften en dreigende opname in ziekenhuis of instelling voor langdurig verblijf. Deze groep komt in aanmerking voor casemanagement.

5.2 Interventie

De interventie die onderzocht wordt is het nieuwe zorgmodel *SamenOud*. Dit model omvat alle basisonderdelen van het CCM. De uitwerking per onderdeel van het CCM is als volgt:

- *‘Organisatie van zorg’* worden de onderdelen ‘Ouderenzorg Teams’ (zie ook §5.2.1), ‘caremanagement’ en ‘casemanagement’ (zie ook §5.2.2) ontwikkeld en in praktijk gebracht.
- *‘Zelfmanagementsupport en preventie’* wordt een programma samengesteld dat toegepast wordt door de ouderenadviseurs en leden van het OT. Het doel van het onderdeel zelfmanagementsupport is de zelfredzaamheid en het zelfmanagementvermogen van de oudere en mantelzorger te handhaven of te bevorderen (zie ook §5.2.3).
- *‘Klinische informatiesystemen’* wordt een Elektronisch Ouderen Dossier (EOD) (verder) ontwikkeld dat ten dienste zal staan van de zorg en het management van de zorg door de Ouderenzorg Teams. De casemanagers en ouderenadviseurs zullen tijdens de huisbezoeken gebruik maken van een tablet PC (iPad).

- *'Beslissingsondersteuning'* wordt gebruik gemaakt van de ouderenprofielen op basis van complexiteit van de zorgbehoeften en mate van kwetsbaarheid. Binnen de groep ouderen wordt onderscheid gemaakt tussen ouderen met complexe zorgbehoeften (INTERMED score ≥20) en ouderen zonder complexe zorgbehoeften (INTERMED score <20). Voor ouderen met complexe zorgbehoeften is de interventie vooral gericht op care- en casemanagement. Voor ouderen zonder complexe zorgbehoeften is vooral gericht op zelfmanagementsupport en preventie.

Voorafgaand aan de interventie:

- Worden de leden van het OT getraind op onderdelen van het zorgaanbod die nieuw zijn ten opzichte van de huidige werkwijze. Het gaat vooral om de onderdelen gerelateerd aan het CCM, zoals proactief en preventief denken en handelen, verlenen van zelfmanagement support, werken met een zorgleefplan en het EOD en de iPad, etc.
- Maken de maatschappelijke en gezondheidszorgorganisaties, met ondersteuning van de projectleiders, afspraken over het effectief en efficiënt organiseren van de zorg rondom de OTs.

Het onderdeel *'Organisatie van zorg',* (Ouderenzorg Team, caremanagement en casemanagement) en het onderdeel *'Zelfmanagementsupport en preventie'* worden in onderstaande subparagrafen uitgebreider beschreven.

5.2.1 Onderdeel 1: Organisatie van zorg – Ouderenzorg Team (OT)

Een OT bestaat minimaal uit de huisarts van de oudere, een specialist ouderengeneeskunde, een gespecialiseerd verpleegkundige of wijkverpleegkundige (verder casemanager genoemd) en een ouderenadviseur.
Een OT onderhoudt structureel en incidenteel intensieve contacten met de lokale maatschappelijke- (welzijn en gemeente) en gezondheidszorgorganisaties en bevindt zich dichtbij de ouderen in de huisartsenpraktijk.
Een OT neemt zelf initiatieven tot reguliere contacten (minimaal jaarlijks) met de oudere en mantelzorger. Met het triage-instrument worden gegevens verzameld over de complexiteit van de zorgbehoeften, de kwetsbaarheid en het welzijn van alle 75-plussers in de huisartspraktijk. Met deze gegevens wordt duidelijk: 1) welke ouderen complexe zorgbehoeften hebben en care- of casemanagement nodig hebben, en 2) welke ouderen gebaat kunnen zijn bij het zelfmanagementsupport en preventieprogramma.

5.2.2 Onderdeel 2: Organisatie van zorg – Care- en casemanagement

De ouderen met complexe zorgbehoeften worden thuis bezocht door de casemanager die op systematische wijze gegevens verzamelt over de domeinen wonen, welzijn en zorg. Vast onderdeel van de gegevensverzameling is 'veiligheid en preventie' en het 'zelfmanagementvermogen' van de oudere. Afgestemd op de wensen en mogelijkheden van de oudere wordt door het OT – in overleg met de oudere zelf en de eventuele mantelzorger(s) – een zorgleefplan opgesteld. Nadat het zorgleefplan met de oudere is vastgesteld, wordt het plan gerealiseerd onder verantwoordelijkheid van het OT en in nauwe samenwerking met betrokken organisaties en hulpverleners. Care- en casemanagement wordt door eenzelfde functionaris (de wijkverpleegkundige van het OT) uitgevoerd, onder leiding van de huisarts en de specialist ouderengeneeskunde. Het is vooral de casemanager die in contact staat met de oudere en eventuele mantelzorger(s). Indien nodig of indien gewenst brengt de huisarts of de specialist ouderengeneeskunde een huisbezoek bij de oudere. De duur van de begeleidingsperiode, m.a.w. de behoefte aan care- of casemanagement, kan van tevoren door het OT worden ingeschat, maar kan pas achteraf

vastgesteld worden. Kortdurende intensieve begeleiding door het OT (caremanagement) is mogelijk bij eenvoudig op te lossen problemen of in acute situaties. De stappen die genomen worden bij ouderen met complexe zorgbehoeften zijn:

STAP 1: Anamnese tijdens huisbezoek bij oudere.

- De casemanager neemt een uitgebreide anamnese af met aandacht voor wonen, welzijn en zorg en specifieke aandacht voor preventie en het zelfmanagementvermogen van de oudere. Met deze uitgebreide anamnese, die in totaal 1,5 tot 2 uur duurt, zijn positieve ervaringen opgedaan in de pilot Casemanagement.
- De casemanager inventariseert behoeften en voorkeuren van de oudere (en diens eventuele mantelzorger) in relatie tot geconstateerde zorgbehoeften. Deze worden opgenomen in het concept zorgleefplan.
- Wanneer de oudere een mantelzorger heeft, wordt in overleg met de oudere en de mantelzorger gegevensverzameling over de mantelzorgbelasting overwogen (na informed consent van de mantelzorger). Aansluitend worden behoeften en mogelijke oplossingen geïnventariseerd. Deze worden opgenomen in het concept zorgleefplan.

STAP 2: Bespreken van het concept zorgleefplan door het OT.

- Het concept zorgleefplan wordt tijdens structureel OT overleg besproken en aangevuld.
- Alle leden van het OT bestuderen de anamnese en het medisch dossier van de oudere.
- De casemanager brengt de behoeften en voorkeuren van de oudere in.
- Alle leden doen voorstellen voor het concept-zorgleefplan, inclusief adviezen voor preventie en zelfmanagement.
- De voorlopige begeleidingsperiode wordt vastgesteld: drie tot zes maanden (caremanagement) of continu (casemanagement).

STAP 3: Vaststellen van het zorgleefplan tijdens huisbezoek.

- De casemanager bespreekt het concept-zorgleefplan en de begeleidingsperiode met de oudere en stelt dit – na eventuele aanpassingen – vast.

STAP 4: In werking stellen van het zorgleefplan.

- Uitvoeren van de beschreven acties in het zorgleefplan door leden van het OT, oudere en/of mantelzorger en het inschakelen van relevante disciplines en/of organisaties door het OT.
- Coördineren en afstemmen van de behandelingen door de medici van het OT indien er sprake is van medische behandeling door meerdere medisch specialisten. Waar nodig zoekt het OT contact met de specialist(en).
- De casemanager coördineert en bewaakt de niet-medische aspecten van het zorgleefplan en onderhoudt de contacten met welzijn, de gemeente (WMO), paramedici, etc.

STAP 5: Monitoring (evalueren voortgang) en navigatie (aansturen) door OT.

- Start na het vaststellen van het zorgleefplan en eindigt op de gestelde evaluatiedatum.
- De casemanager overlegt (minimaal tweewekelijks) met de oudere en/of mantelzorger over de realisatie van de activiteiten zoals beschreven in het zorgleefplan en rapporteert dit in het zorgleefplan. Afhankelijk van de situatie vindt dit overleg plaats tijdens een huisbezoek of door middel van telefonisch contact.
- Het OT overlegt (minimaal vierwekelijks) over de voortgang in de realisatie van de activiteiten zoals beschreven in het zorgleefplan en rapporteert in het zorgleefplan.
- Afstemmen en aansturen van activiteiten van hulpverleners en organisaties om de voortgang in het realiseren van het zorgleefplan te bevorderen.

- Aanvullen van het zorgleefplan met informatie over de voortgang van de activiteiten zoals beschreven in het zorgleefplan en/of aanvullen met gegevens van nieuw ontstane problematiek en daaruit voortkomende activiteiten.

STAP 6: Evaluatie (casemanager).

- Gericht op het afronden van het zorgleefplan en de intensieve begeleidingsperiode van de oudere en, indien relevant, diens mantelzorger.
- Omvat zowel een proces- (hoe is het gegaan) als productevaluatie (realiseren van de doelen).
- Vindt plaats tijdens een huisbezoek aan het einde van de vastgestelde begeleidingsperiode (caremanagement) of aan het einde van de interventieperiode (casemanagement).
- Afhankelijk van de uitkomsten: continueren begeleidingsperiode (care-/casemanagement) of ‘overdracht’ naar de ouderenadviseur (zelfmanagementsupport en preventie).

Voorbeeld 1: Caremanagement

Dhr. A. moet voor een operatie aan zijn prostaat naar het ziekenhuis. Voor zijn welbevinden is het beter dat dhr. A. zo kort mogelijk in het ziekenhuis verblijft. Het OT regelt – door inzet van de lokale thuiszorgorganisaties en verpleeg- en verzorgingshuizen – dat de opname in het ziekenhuis zo kort mogelijk duurt, en begeleidt de hele procedure totdat dhr. A. voldoende hersteld is.

Voorbeeld 2: *Caremanagement*

Dhr. J. heeft diabetes, is sinds een jaar weduwnaar, verwaarloost zichzelf in toenemende mate en weigert alle hulp. Na een intensieve periode van gesprekken met dhr. J., zijn kinderen en diverse organisaties, verhuist dhr. J. naar ieders tevredenheid naar een verzorgingshuis.

Voorbeeld 3: Casemanagement

Dhr. E., bekend met ernstig hartfalen, is in toenemende mate beperkt in energie en activiteiten. Zijn echtgenote is in toenemende mate vergeetachtig en ook fysiek steeds meer beperkt. Samen met het echtpaar en de kinderen organiseert het OT o.a. dagopvang en huishoudelijke ondersteuning. De kinderen komen regelmatig op bezoek en ook een ouderenadviseur wordt ingeschakeld. Dagelijks komt er nu iemand bij het echtpaar over de vloer waardoor een stabiele, zij het wankele, situatie wordt bereikt. In overleg met het OT wordt bij dhr. E. de bloeddrukverlagende medicatie verminderd, waardoor het risico op vallen afneemt, en de cholesterolverlagende medicatie gestopt. De casemanager monitort de gezondheid (o.a. de bloeddruk van dhr. E.) en de zorgbehoeften van het echtpaar tijdens regelmatige bezoekjes, en is bereikbaar voor alle betrokkenen. Zo wordt een opname – voor kortdurend of langdurig verblijf – voorlopig uitgesteld.

5.2.3 Onderdeel 3: Zelfmanagementsupport en preventie

Dit onderdeel richt zich vooral op ouderen zonder complexe zorgbehoeften maar met een toenemende kwetsbaarheid. Het programma heeft tot doel het zelfmanagement vermogen en de zelfredzaamheid van de oudere te ondersteunen of te bevorderen. Doel is dat de rol en invloed van de oudere in het proces van ouder worden wordt versterkt en de periode van zelfredzaamheid en autonomie zo lang als mogelijk gehandhaafd blijft. De leden van het OT, maar vooral de ouderenadviseurs, kunnen in overleg met de oudere gerichte ondersteuning bieden op individueel- of groepsniveau aan de oudere (o.a. GRIP- en GLANS-cursussen). Onderwerpen gericht op preventie van de meest voorkomende en risicovolle gezondheidsproblemen bij ouderen zijn: eenzaamheid en sociale participatie, voeding, bewegen, veiligheid (vallen) in en om huis, etc.

Uit recente, nog niet gepubliceerde, gegevens verkregen met het nieuwe triage-instrument blijkt dat een klein deel van de ouderen (±20%) die volgens de INTERMED score geen complexe zorgbehoeften heeft, verhoogd kwetsbaar is (GFI ≥4) en een verhoogd risico op complexe zorgbehoeften heeft. Deze ouderen krijgen individuele begeleiding (A) door de ouderenadviseur geboden. Het doel van individuele begeleiding is het voorkomen van of tijdige signalering van toename van de complexiteit. De overige ouderen (±80%) in deze groep zijn minder kwetsbaar (GFI ≤3) en hebben minder risico op complexe zorgbehoeften. Deze ouderen krijgen groepsactiviteiten (B) aangeboden met als doel hen voor te bereiden op een afname van zelfredzaamheid.

A. Individuele begeleiding

De individuele begeleiding van de ouderen door de ouderenadviseur vindt volgens hetzelfde stramien plaats als bij de ouderen die case- of caremanagement krijgen (zie §5.2.2).

STAP 1: Anamnese tijdens huisbezoek bij oudere.

- De ouderenadviseur neemt een uitgebreide anamnese af met aandacht voor wonen, welzijn en zorg en specifieke aandacht voor preventie en het zelfmanagementvermogen van de oudere. Deze uitgebreide anamnese, duurt in totaal 1,5 tot 2 uur duurt.
- De ouderenadviseur inventariseert behoeften en voorkeuren van de oudere (en diens eventuele mantelzorger) in relatie tot geconstateerde kwetsbaarheid.
- Wanneer de oudere een mantelzorger heeft, wordt in overleg met de oudere en de mantelzorger (na informed consent) gegevensverzameling over de mantelzorgbelasting overwogen. Aansluitend worden behoeften en mogelijke oplossingen geïnventariseerd.

STAP 2: Opstellen concept zorgleefplan.

- Het concept zorgleefplan wordt, samen met de oudere (en eventueel de mantelzorger), door de ouderenadviseur opgesteld.

STAP 3: Bespreken van het concept zorgleefplan:

- Het concept zorgleefplan wordt tijdens structureel overleg tussen ouderenadviseur en casemanager besproken en aangevuld.
- De ouderenadviseur brengt de behoeften en voorkeuren van de oudere in.
- De casemanager doet voorstellen voor het zorgleefplan, inclusief adviezen voor preventie en zelfmanagement.

STAP 4: Vaststellen van het zorgleefplan tijdens huisbezoek.

- De ouderenadviseur bespreekt het concept zorgleefplan met de oudere en stelt dit – na eventuele aanpassingen – vast. Indien relevant wordt het zorgleefplan ook met de mantelzorger besproken.

STAP 5: In werking stellen van zorgleefplan.

- Uitvoeren van de beschreven acties in het zorgleefplan door de ouderenadviseur, oudere en/of mantelzorger(s) en het inschakelen van relevante disciplines en/of organisaties, zoals welzijn.
- De ouderenadviseur coördineert en bewaakt de niet-medische aspecten van het zorgleefplan en onderhoudt de contacten met welzijn, de gemeente (WMO), paramedici, etc.

STAP 6: Monitoring (evalueren voortgang) en navigatie (aansturen).

- Start na het vaststellen van het zorgleefplan en eindigt op de gestelde datum.
- De ouderenadviseur overlegt periodiek (afhankelijk van de doelen) met de oudere en/of mantelzorger over de realisatie van de activiteiten zoals beschreven in het zorgleefplan en rapporteert dit in het zorgleefplan. Afhankelijk van de situatie wordt dit tijdens huisbezoek of door middel van telefonisch contact gedaan.

- Structureel overleg tussen ouderenadviseur en casemanager (iedere maand): de ouderenadviseur informeert de casemanager over actuele situaties en ervaren problemen, waarbij de casemanager de ouderenadviseur ondersteunt.
- Incidenteel en individueel: informeren en/of doorverwijzen van oudere naar het OT bij toename van de complexiteit van de zorgbehoeften.
- De ouderenadviseur stemt de activiteiten van hulpverleners en organisaties af om de voortgang in het realiseren van het zorgleefplan te bevorderen.
- Aanvullen van het zorgleefplan met informatie over de voortgang van de activiteiten zoals beschreven in het zorgleefplan en/of aanvullen met gegevens van nieuw ontstane problematiek en daaruit voortkomende activiteiten.

STAP 7: Evaluatie.

- Gericht op het afronden van het zorgleefplan en de intensieve begeleidingsperiode van de oudere en, indien relevant, diens mantelzorger.
- Omvat zowel een proces- (hoe is het gegaan) als productevaluatie (realiseren van de doelen).
- Vindt plaats tijdens een huisbezoek aan het einde van de interventieperiode.
- Afhankelijk van de uitkomsten: continueren begeleidingsperiode of ‘overdracht’ naar de casemanager (caremanagement).

B. <u>Groepsinterventies</u>

Ouderen ontvangen van de ouderenadviseur:

- Informatie en een uitnodiging voor groepsinterventies gericht op het verhogen van het zelfmanagementvermogen (o.a. GLANS-cursussen).
- De mogelijkheid van inventarisatie, advisering en ondersteuning bij het realiseren van preventieve maatregelen tegen potentiële risico’s van het ouder worden. Voorbeelden van onderwerpen zijn voeding, mobiliteit, sociale contacten en valpreventie. Bij de invulling van deze groepsinterventies worden leden van het OT, de plaatselijke welzijnsorganisatie en gemeente (WMO) betrokken.

Voorbeeld 4: Zelfmanagementsupport en preventie

Mevr. K. woont alleen, wordt steeds slechter ter been en heeft steeds meer moeite met het doen van boodschappen. Hierdoor dreigt het risico op vallen en wellicht ondervoeding en eenzaamheid. De ouderenadviseur regelt met mevr. K. dat iemand samen met haar boodschappen gaat doen, en ook zal zij kunnen deelnemen aan een beweeggroep voor ouderen in de buurt. Verder wordt de ergotherapeut ingezet voor loopondersteuning en screening van de woning waarna mevr. K. een rollator krijgt en aanpassingen in huis worden gedaan (beugels, douchestoel etc.) om het valrisico te beperken. Ook wordt, in overleg met het OT, het slaapmiddel – dat mevr. K. al vele jaren gebruikt – met succes afgebouwd. Mevr. K. voelt zich nu fitter en alerter. Ze denkt zelfs na over het deelnemen aan de GLANS-cursus.

5.3 Gebruikelijke zorg

Ouderen en hun mantelzorgers die niet geïncludeerd worden in de interventiegroep ontvangen de gebruikelijke zorg en begeleiding van de huisarts en van andere hulpverleners en hulpverlenende organisaties. Dit houdt in dat de oudere zich meldt bij de huisarts als zich een medisch probleem voordoet, eventueel medisch specialisten bezoekt volgens herhalingsconsulten, ondersteuning vraagt via de diverse loketten, etc.

6. METHODE

6.1 Uitkomstmaten en meetinstrumenten

De af te nemen vragenlijsten per meetmoment zijn weergegeven in Tabel 1, blz. 28

6.1.1 Oudere – Primaire uitkomstmaat

Primaire uitkomstmaat is de complexiteit van de zorgbehoeften (INTERMED-score gemeten met het triage-instrument[1]).

- o INTERMED lijst: Deze lijst meet de complexiteit van de (zorg)behoeften in vier domeinen: biologische, psychologische en sociale behoeften en het zorgsysteem, in verschillende tijdsperspectieven: verleden, heden en toekomst. De INTERMED lijst is ontworpen voor gebruik in zowel intra- als extramurale settings. Het oorspronkelijke instrument wordt ingevuld door zorgverleners en is gebruikt en gevalideerd in veel landen. [15-18] De klinimetrische kwaliteiten van het instrument worden door de resultaten van verschillende studies onderschreven. [17, 19-22] Het instrument is aangepast naar een versie die door de oudere zelf in te vullen is.

6.1.2 Oudere – Secundaire uitkomstmaten

Secundaire uitkomstmaten zijn de mate kwetsbaarheid (Groninger Frailty Indicator (GFI)) en het welbevinden (Welbevindenlijst) (beiden onderdeel van het triage-instrument), het welbevinden gemeten met de Minimale Data Set (MDS) (RAND 36-item Health Survey (RAND-36) en Cantrill's Ladder), het zelfmanagementvermogen (Self-Management Abilty Scale (SMAS-30)), zelfmanagementkennis (Partners In Health Scale (PIH scale)) en kwaliteit van leven (EQ-5D in de MDS).

- o GFI: Deze vragenlijst meet kwetsbaarheid en bestaat uit vijftien items, is eenvoudig in te vullen door ouderen en heeft een goede predictieve validiteit. Het instrument wordt in vele verschillende ouderenzorg settings gebruikt, zowel nationaal als internationaal. [13-14]
- o Welbevindenlijst: Deze lijst stelt zaken vast die veel mensen belangrijk vinden in het dagelijkse leven. Dit nieuwe instrument is een generiek instrument dat het welbevinden van ouderen kan meten in verschillende situaties. Het instrument bestaat uit acht items op het gebied van dagelijkse ervaringen (bijv. genieten van eten en drinken; lekker slapen en rusten; plezierige relaties en contacten). De oudere wordt gevraagd of ze de ervaring belangrijk vinden en, wanneer dat het geval is, of ze op dat gebied tevreden zijn in hun dagelijkse leven.
- o MDS Zorgvrager – Samengestelde lijst: Het is de bedoeling dat de uitkomsten van de verschillende regio's die meedoen aan het Nationaal Programma Ouderenzorg (NPO) onderling vergelijkbaar zijn. Hiervoor is door het NPO een onderzoeksinstrument ontwikkeld en verplicht gesteld: de Minimale Data Set (MDS). De MDS is een document waarin gevalideerde onderzoeksvragen staan. Er zijn vragenlijsten voor de zorgvrager en vragenlijsten voor de mantelzorger. Per doelgroep zijn er twee versies, één versie voor de beginmeting (basismeting), de tweede versie voor de vervolgmeting. De vragenlijsten zijn als volgt opgebouwd:

[1] NB. De cliëntversie van het triage-instrument wordt gevalideerd in het NPO Regio Noord onderzoek 'Het Triage-instrument: (predictieve) validiteit van de Groninger Frailty Indicator, de INTERMED cliënt versie en de Welbevinden lijst'.

- Algemene vragen: leeftijd, geslacht, sociaal economische status (= postcode), etniciteit, opleidingsniveau, burgerlijke staat, woonsituatie.
- Ervaren gezondheid (EQ-5D), utiliteit zorgvrager (EQ-5D), multimorbiditeit (GGD-monitor), functioneren (Katz-15).
- Welbevinden: psychisch, sociaal, kwaliteit van leven (RAND-36 vragen en Cantrill's Ladder).
- Zorggebruik: ziekenhuisopname, ongeplande huisartszorg, thuiszorg, opname verzorgingshuis, dagopvang, dagbehandeling.

o SMAS-30: Voor het evalueren van het effect van het zelfmanagementsupport wordt de SMAS-30 toegepast. De SMAS-30 is gebaseerd op het concept zelfmanagement in relatie tot welbevinden. De vragenlijst bestaat uit 30 vragen met vijf of zes antwoordcategorieën (Likert schaal). Scores worden gesommeerd en getransformeerd naar een overall schaal score (range 0-100). Een hogere score duidt op een hoger zelfmanagement vermogen. De interne consistentie is goed (Cronbach's Alpha = .92).
NB. De SMAS-30 wordt tijdens het eerste huisbezoek door de casemanager of ouderenadviseur uitgereikt aan de ouderen in de interventiegroep. De reden hiervoor is praktisch van aard: de vragenlijst is dusdanig lang dat het een te grote belasting zou vormen voor de ouderen om deze aan het eerste meetmoment T0 of aan de anamnese zelf toe te voegen.

o PIH scale: De Partners In Health Scale (PIH) meet de zelfmanagementkennis en gedrag van de oudere in relatie tot het ouder worden en de chronische aandoeningen die hij of zij heeft. In 2010 is deze vragenlijst, die bestaat uit 12 items, gevalideerd [29]. Deze vragenlijst richt zich op onderdelen van het Chronic Care Model en in tegenstelling tot de PACIC niet op de professionals, maar juist op de oudere. [30]

6.1.3 Mantelzorger – Primaire uitkomstmaat

Primaire uitkomstmaat is de mantelzorgbelasting, gemeten met de Caregiver Strain Index (CSI).

o CSI: De Caregiver Strain Index (CSI) meet de ervaren zorglast van de mantelzorger. De CSI is een veelgebruikt meetinstrument om de belasting van mantelzorgers te meten [25] en heeft een goede betrouwbaarheid en construct validiteit. [26] De lijst bestaat uit dertien stellingen, die met 'ja' of 'nee' beantwoord worden. Indien zeven keer of vaker een stelling met 'ja' wordt beantwoordt, is er sprake van overbelasting van de mantelzorger. De stellingen beslaan vijf domeinen, te weten werk, financieel, fysiek, sociaal en tijdsbesteding. [26]

6.1.4 Mantelzorger – Secundaire uitkomstmaten

Secundaire uitkomstmaten zijn de mantelzorgbelasting gemeten met de Self-rated Burden Visual Analogue Scale (Self-rated Burden VAS) en de Carer Quality of Life (Carer QOL), de ervaren gezondheid (RAND-36) en de ervaren kwaliteit van leven (RAND-36 en Cantrill's Ladder). Deze secundaire uitkomsten zijn allemaal deel van de MDS Mantelzorger versie.

o MDS Mantelzorger – Samengestelde lijst:
  - Algemene vragen: leeftijd, geslacht, sociaal economische status (= postcode), leeftijd zorgvrager, geslacht zorgvrager, sociaal economische status zorgvrager, relatie tot zorgvrager, samenwonend met zorgvrager zelf.
  - Ervaren gezondheid (RAND-36 vragen)
  - Belasting mantelzorg: objectief (iBMG meetinstrument) en subjectief (Self-rated Burden VAS en Carer QOL).
  - Ervaren kwaliteit van leven (RAND-36 en Cantrill's Ladder).

6.1.5 Kwaliteit van zorg – Primaire uitkomstmaat

Primaire uitkomstmaat van kwaliteit van zorg is de mate van complexiteit van de zorgbehoeften van de oudere (INTERMED-score gemeten met het triage-instrument) (zie §6.1.1).

6.1.6 Kwaliteit van zorg – Secundaire uitkomstmaten

Secundaire uitkomstmaat is de mate waarin doelen van het zorgleefplan gerealiseerd zijn (Goal Attainment) en de ervaringen en tevredenheid van ouderen over de geleverde zorg (Patient Assessment of Chronic Illness Care (PACIC)).

- Goal Attainment: De methode Behaalde doelen (Goal Attainment Scaling [23]) blijkt vooral in complexe gezondheidssituaties een effectieve methode voor het meten van verandering. [24] In de zorgleefplannen worden doelen geformuleerd en aan het einde van de begeleidingsperiode wordt nagegaan in welke mate deze doelen behaald zijn. De werkwijze is als volgt: nadat de casemanager of ouderenadviseur en de oudere een gezondheidsprobleem geformuleerd hebben, wordt in overleg een zo concreet en haalbaar mogelijk doel geformuleerd vanuit het perspectief van de oudere. Vervolgens wordt een actieplan opgesteld dat gericht is op het behalen van de doelstelling. Dit wordt in het zorgleefplan beschreven. Tijdens de evaluatie van het zorgleefplan wordt nagegaan in hoeverre de doelen in het zorgleefplan, naar het oordeel van de oudere en de casemanager, behaald zijn. Antwoordcategorieën per doelstelling zijn: 0 = niet behaald; 1 = gedeeltelijk behaald; 2 = grotendeels behaald; 3 = behaald. Wanneer de situatie verslechterd is tijdens de interventie: -1 = niet behaald en enigszins verslechterd; -2 = verslechterd; -3 = ernstig verslechterd.
  Per zorgleefplan worden de scores gesommeerd in een totaalscore en omgerekend naar een percentage:
  - Hoogst haalbare score = aantal doelen x maximaal haalbare score
  - Behaalde score = aantal doelen x behaalde totale score
  - Percentage behaalde doelen = (behaalde score / hoogst haalbare score) x 100%

  Hoe hoger het percentage behaalde doelen, des te beter is de kwaliteit van zorg.
- PACIC: De PACIC meet specifieke acties of elementen van kwaliteit van zorg, die in lijn zijn met (onderdelen van) het Chronic Care Model en die de oudere heeft ervaren gedurende de interventieperiode. De vragenlijst omvat 20 items en is in 2005 gevalideerd. [27] Een recente studie van Vrijhoef e.a. heeft uitgewezen dat de PACIC op dit moment het beste instrument is om de cliëntervaringen betreffende integrale zorg te meten. [28]

6.1.7 Zorggebruik en kosten

Onderdeel van de beoordeling van het effect van het nieuwe zorgmodel *SamenOud* is de evaluatie van de balans tussen kosten en baten van het nieuwe zorgmodel ten opzichte van de gebruikelijke zorg. Deze studie wordt uitgevoerd door een gezondheidseconoom van de afdeling Health Technology Assessment (HTA) bureau van het UMCG volgens de daarvoor geldende methoden en middelen.
Het overall verschil in kosten (incrementele kosten) tussen de zorg volgens het nieuwe zorgmodel ten opzichte van de gebruikelijke zorg, zal worden geschat (inclusief 95% CI) vanuit een maatschappelijk perspectief. Met gebruikmaking van de primaire uitkomstmaten van het nieuwe zorgmodel (complexiteit van zorgbehoeften) wordt de verhouding tussen kosten en baten gepresenteerd.
Naast de berekening van de incrementele kosten wordt met de EQ-5D (uit de MDS) ook het effect van het nieuwe zorgmodel ten opzichte van de gebruikelijke zorg in termen van kosten per gewonnen Quality Adjusted Life Year (QALY) geschat.

In dit transitie-experiment is een korte termijn analyse met een tijdshorizon van één jaar gepland. Mocht dit onvoldoende blijken om een weloverwogen besluit tot brede implementatie te nemen, dan kan een verdere analyse met extrapolatie van de gegevens worden uitgevoerd. Bootstrapping zal worden toegepast voor de beoordeling van de onzekerheid over de puntschatting van de Incremental Cost-Effectiveness Ratios (ICER), gebaseerd op de waargenomen gegevens. Vervolgens zal in een kosteneffectiviteit–acceptatie curve de mate van 'besluitonzekerheid' worden weergegeven, rekening houdend met een toenemende maatschappelijke bereidheid om voor het nieuwe zorgaanbod te betalen.
Discontering zal met het oog op de korte tijdshorizon niet worden toegepast.

- <u>Zorgleefplannen:</u> Om de zorgintensiteit van de ouderen in de caremanagement- en casemanagementgroep te kunnen meten wordt per oudere de duur van de begeleidingsperiode en de tijdsinvestering tijdens de begeleidingsperiode door de casemanager geregistreerd. Van de ouderen in de zelfmanagementgroep wordt per oudere het volgende geregistreerd door de ouderenadviseur: tijdsinvestering op individueel niveau, deelname aan groepsactiviteiten gericht op zelfmanagementsupport en preventie, overlegmomenten met de casemanager (frequentie en incidenteel of structureel overlegmoment) en doorverwijzingen naar ergotherapie, WMO (huishoudelijke hulp of hulpmiddelen).
- <u>QALY:</u> De Qaly wordt bepaald door gegevens van zorggebruik en kosten te combineren met kwalitatieve gegevens, in dit geval de ervaren gezondheid (EQ-5D) uit de MDS. De QALY wordt gebaseerd op het aantal (extra) levensjaren ten gevolge van de interventie en de daarmee gemaakte kosten, en een correctiefactor voor de kwaliteit van leven. Afhankelijk van de mate en ernst van de beperking(en) ligt deze correctiefactor tussen 0.0 en 1.0.

Tabel 1 *Af te nemen vragenlijsten per meetmoment*

Deelnemer	Uitkomstmaat	Vragenlijst	T0		Anamnese		$T_{variabel}$		T1	
			Interventie-groep	Controle-groep	Interventie-groep	Controle-groep	Interventie-groep	Controle-groep	Interventie-groep	Controle-groep
Oudere	Primair	INTERMED	√	√			√		√	√
	Secundair	GFI	√	√					√	√
		Welbevindenlijst	√	√					√	√
		MDS zorgvrager basismeting	√	√						
		MDS zorgvrager vervolgmeting							√	√
		SMAS-30			√				√	
		PIH scale	√	√					√	√
Mantelzorger	Primair	CSI	√						√	
	Secundair	MDS mantelzorger basismeting	√							
		MDS mantelzorger vervolgmeting							√	
Kwaliteit van zorg	Primair	INTERMED	√	√			√		√	√
	Secundair	Goal Attainment			√ (continu)					
		PACIC							√	√
Zorggebruik en kosten		QALY	√	√					√	√
		Zorgleefplan			√ (continu)					

6.2 Randomisatie, blinding and treatment allocation

Na inclusie worden (per huisartspraktijk) de ouderen ingedeeld in twee strata van complexiteit van zorgbehoeften, op basis van gegevens verkregen met het triage-instrument (complexiteit van zorgbehoeften) (Figuur 3). Ouderen met een INTERMED score van 20 of hoger (complexe zorgbehoeften) komen in aanmerking voor care- /casemanagement. Ouderen met een INTERMED score lager dan 20 (geen complexe zorgbehoeften) komen in aanmerking voor zelfmanagementsupport en preventie. Binnen de strata worden de respondenten met de toewijzingstechniek van balanceren gerandomiseerd naar de controlegroep (die gebruikelijke zorg ontvangt) of de interventiegroep (die zorg volgens het nieuwe zorgmodel ontvangt).

De software die gebruikt wordt voor random toewijzing aan gebruikelijke zorg of aan de interventie resulteert tevens in een gelijke verdeling van prognostische en confounding kenmerken in beide groepen. [31-33] De balanceringsvariabelen zijn, onder andere gebaseerd op de bevindingen in de pilot Casemanagement voor ouderen met complexe zorgbehoeften: wel/niet ontvangen van huishoudelijke hulp en/of thuiszorg. Daaraan toegevoegd zijn de variabelen: leeftijd, geslacht, opleidingsniveau, woonsituatie, wel/geen partner, wel/geen mantelzorger, wel/geen kinderen, wel/geen hulp bij het invullen van de vragenlijst en wel/geen chronische ziekte.

NB. Randomisatie op het niveau van de huisartspraktijken is overwogen. Gekozen is voor het voordeel van implementatie van het nieuwe zorgmodel in een groter aantal huisartspraktijken. Het risico op overloop van het behandeleffect wordt geschat als gering: ouderen in de pilot Casemanagement meldden zich zelden bij de huisarts, en bovendien is de gemiddelde consulttijd kort (6-10 min.) en gericht op het oplossen van de concrete vraag.

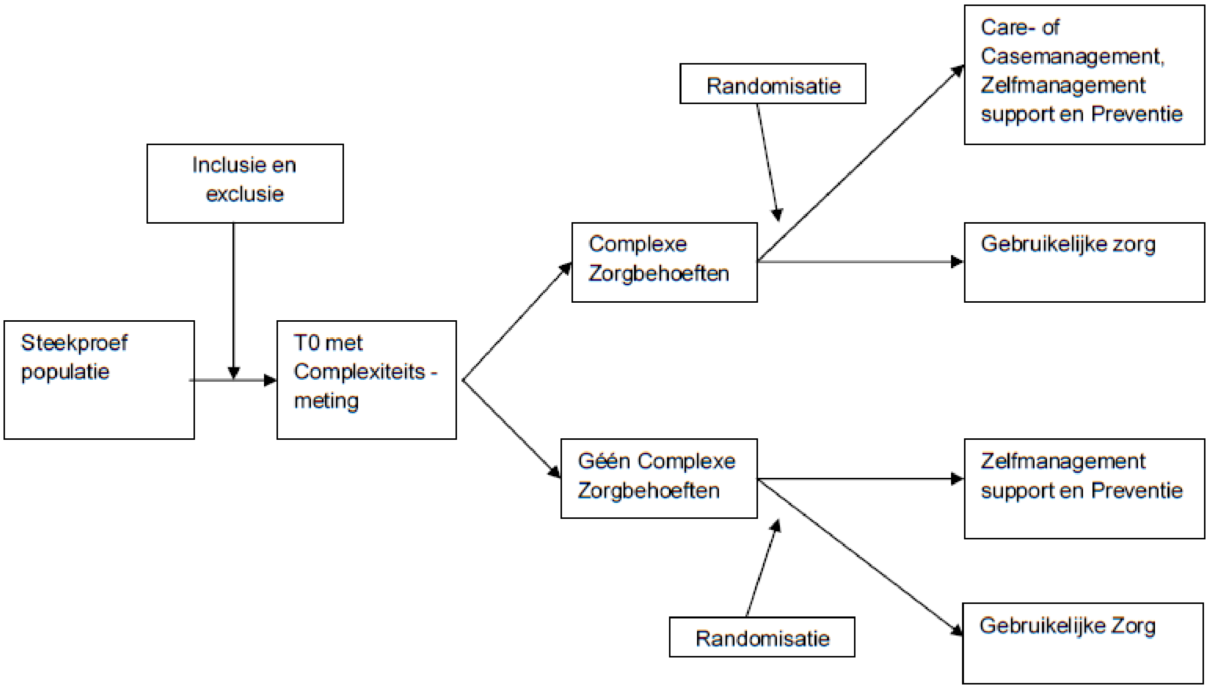

Figuur 3 *Randomisatieprocedure met balancering per huisartsenpraktijk*

6.3 Studieprocedures

6.4.1 Dataverzameling T0

OUDEREN

De procedure van dataverzameling bij de ouderen wordt in stappen uitgevoerd.

STAP 1: Selectie ouderen

De huisartsen selecteren ouderen van 75 jaar en ouder en die voldoen aan de inclusie- en exclusiecriteria van het onderzoek.

STAP 2: Informatiebrief voor ouderen

Ouderen worden namens de huisarts via de post benaderd met het verzoek om deelname aan het onderzoek te overwegen. Het pakket bevat een begeleidende brief namens de huisarts, een informatiefolder, een informed consent formulier en de vragenlijsten.

Voor vragen kunnen ouderen terecht bij de telefonische helpdesk van het onderzoek. Medewerkers van welzijn en vrijwilligers worden ingezet om eventuele assistentie bij het invullen van de vragenlijsten en het informed consent formulier te geven (d.m.v. huisbezoek).

STAP 3: Herinnerings-/bedankbrief

Twee weken na de verzending van de eerste brief ontvangen respondenten en non-respondenten een herinnerings-/bedankbrief. Doel van deze brief is om non-respondenten aan te sporen de vragenlijst alsnog in te vullen. Respondenten die de vragenlijst en het informed consent al ondertekend hebben teruggestuurd, worden in deze brief bedankt voor deelname.

STAP 4: Herinneringsbrief voor non-respondenten

Vijf weken na de verzending van de eerste brief worden non-respondenten nogmaals benaderd met een herinneringsbrief. Verwachtte respons na herinneringen is 70%. Deze relatief hoge responsverwachting lijkt reëel: diverse onderzoeken meldden een respons onder ouderen van meer dan 80%. Het feit dat de brief van de eigen huisarts komt werkt over het algemeen ook responsverhogend.

STAP 5: Verwerking ingevulde vragenlijsten

De geretourneerde vragenlijsten worden ingevoerd en verwerkt en de complexiteit van de zorgbehoeften wordt vastgesteld. Deze gegevens worden pas na randomisatie doorgeven aan OT, en dan alleen van de ouderen in de interventiegroep**.**

STAP 6: Randomisatie

Geïncludeerde ouderen worden gestratificeerd naar de 'complexiteitgroepen' en gerandomiseerd naar de interventie- of controlegroep.

MANTELZORGERS

Vooral om praktische redenen (identificatie van mantelzorgers) worden alleen mantelzorgers van ouderen met complexe zorgbehoeften en verhoogde kwetsbaarheid in de interventiegroep benaderd voor deelname aan het onderzoek (zie ook blz. 16 Inclusie- en exclusiecriteria).

De procedure van dataverzameling bij de mantelzorgers wordt als volgt uitgevoerd:

STAP 1: Toestemming oudere

Uit het bezoek door de casemanager of ouderenadviseur aan de oudere wordt duidelijk of de oudere een mantelzorger heeft of niet. Is dat het geval, dan vraagt de casemanager of ouderenadviseur toestemming aan de oudere om diens mantelzorger te benaderen voor deelname aan het onderzoek.

STAP 2: Informatiebrief aan mantelzorgers

Na toestemming van de oudere wordt de geïdentificeerde mantelzorger namens de huisarts via de post benaderd (op het postadres van de oudere) met de vraag of hij/zij wil deelnemen aan het onderzoek. Het pakket voor de mantelzorger bevat een begeleidende brief namens de huisarts, een informatiefolder, informed consent formulier en een vragenlijst. Voor vragen kunnen mantelzorgers terecht bij de telefonische helpdesk van het onderzoek.

STAP 3: Herinnerings-/bedankbrief

Twee weken na de verzending van de eerste brief ontvangen respondenten en non-respondenten een herinnerings-/bedankbrief. Het doel van deze brief is om non-respondenten aan te sporen alsnog de vragenlijst in te vullen en deel te nemen aan het onderzoek. Respondenten die de vragenlijst en het informed consent ondertekend hebben teruggestuurd worden in deze brief bedankt voor deelname.

STAP 4: Herinneringsbrief voor non-respondenten

Vijf weken na de verzending van de eerste brief worden non-respondenten nogmaals benaderd d.m.v. een herinneringsbrief.

STAP 5: Verwerking ingevulde vragenlijsten

De geretourneerde vragenlijsten worden ingevoerd en verwerkt.

6.4.2 Dataverzameling T1

OUDEREN

De procedure van dataverzameling bij de ouderen wordt als volgt uitgevoerd:

STAP 1: Informatiebrief naar ouderen

Ouderen worden namens de huisarts via de post benaderd met het pakket voor de eindmeting. Dit pakket bevat een begeleidende brief namens de huisarts en de vragenlijsten. Voor vragen kunnen ouderen terecht bij de telefonische helpdesk van het onderzoek. Medewerkers van welzijn en vrijwilligers worden ingezet om eventuele assistentie bij het invullen van de vragenlijsten te bieden (d.m.v. huisbezoek).

STAP 3: Herinnerings-/bedankbrief

Twee weken na de verzending van de eerste brief ontvangen respondenten en non-respondenten een herinnerings-/bedankbrief. Doel van deze brief is om non-respondenten aan te sporen de vragenlijst alsnog in te vullen. Respondenten die de vragenlijst al hebben teruggestuurd, worden in deze brief bedankt voor deelname.

STAP 4: Herinneringsbrief voor non-respondenten

Vijf weken na de verzending van de eerste brief worden non-respondenten nogmaals benaderd d.m.v. een herinneringsbrief.

STAP 5: Verwerking ingevulde vragenlijsten

De geretourneerde vragenlijsten worden ingevoerd en verwerkt.

MANTELZORGERS

De procedure van dataverzameling bij de mantelzorgers wordt als volgt uitgevoerd:

STAP 1: Informatiebrief naar mantelzorgers

Mantelzorgers worden namens de huisarts via de post benaderd met de vraag of ze nogmaals de vragenlijsten willen invullen voor het onderzoek. Het pakket bevat een begeleidende brief namens de huisarts en de vragenlijst. Voor vragen kunnen mantelzorgers terecht bij de telefonische helpdesk van het onderzoek.

STAP 3: Herinnerings-/bedankbrief

Twee weken na de eerste verzending ontvangen respondenten en non-respondenten een herinnerings-/bedankbrief. Het doel van deze brief is om non-respondenten aan te sporen alsnog de vragenlijst in te vullen en deel te nemen aan het onderzoek. Respondenten die de vragenlijst en het informed consent ondertekend hebben teruggestuurd worden in deze brief bedankt voor deelname.

STAP 4: Herinneringsbrief voor non-respondenten

Vijf weken na de eerste verzending worden non-respondenten nogmaals benaderd d.m.v. een herinneringsbrief.

STAP 5: Verwerking ingevulde vragenlijsten

De geretourneerde vragenlijsten worden ingevoerd en verwerkt.

6.4.3 Dataverzameling $T_{variabel}$

Indien een oudere door de ouderenadviseur wordt overgedragen naar de casemanager (of omgekeerd) wordt opnieuw de self-report INTERMED (complexiteit van zorgbehoeften) afgenomen. Deze transitie vindt plaats als er een verandering in de complexiteitsscore (INTERMED score) van de oudere is van <20 (niet complex) naar ≥20 (complex) (of omgekeerd). Een score van 20 op de INTERMED geeft de grens aan tussen de groepen case-/caremanagement en zelfmanagementsupport. De ouderenadviseur bepaalt wanneer een oudere overgeplaatst wordt naar de casemanager (of omgekeerd).

6.4.4 Interventie

De te nemen stappen tijdens de interventieperiode worden beschreven in *§5.2 Interventie*.

6.5 Terugtrekken uit de studie

Deelnemers (ouderen en mantelzorgers) aan het onderzoek, zowel in de interventie- als in de controlegroep, kunnen zich te allen tijde en zonder opgaaf van redenen terugtrekken uit de studie. Hieraan zijn geen consequenties verbonden.

6.5.1 Specifieke criteria voor terugtrekken uit de studie

De exclusiecriteria zoals eerder beschreven zijn van toepassing (§4.3): als een deelnemer tijdens de studie aan één of meerdere van de exclusiecriteria voldoet, wordt hij/zij uitgesloten van verdere deelname aan het onderzoek. Indien de deelnemer zich in de interventiegroep bevindt, zal de interventie indien gewenst niet worden afgerond. De gegevens van de deelnemer zullen niet meegenomen worden bij de analyse van de resultaten.

6.6 Vervanging van proefpersonen na terugtrekken uit studie

Als voor aanvang van het interventiejaar of tijdens het interventiejaar ouderen uitvallen of zich terugtrekken, worden er geen nieuwe deelnemers geïncludeerd. Bij de berekening van het aantal te includeren ouderen hebben we rekening gehouden met loss-to-follow-up. Hierdoor kan worden aangenomen dat er voldoende ouderen overblijven om statistische berekeningen te kunnen uitvoeren.

6.7 Follow-up van proefpersonen die uit de studie zijn teruggetrokken

Proefpersonen die zich uit de studie hebben teruggetrokken zullen niet in de follow-up meting (na 12 maanden) van het onderzoek betrokken worden.

6.8 Voortijdige beëindiging van de studie

Het onderzoek zal alleen in hoogst uitzonderlijke gevallen voortijdig beëindigd worden. Het Dagelijks Bestuur van het transitie-experiment zal dan naar een passende oplossing zoeken.

7 VEILIGHEID

7.1 Sectie 10 WMO

De onderzoeker zal de proefpersonen en de METc informeren, volgens sectie 10, subsectie 1 van de WMO, indien het wetenschappelijk onderzoek een verloop neemt dat in noemenswaardige mate voor de proefpersoon ongunstiger is dan in het onderzoeksprotocol is voorzien. Tot het tijdstip waarop een nader positief oordeel wordt gegeven, wordt de uitvoering van het onderzoek opgeschort, tenzij de gezondheid van de proefpersoon opschorting of beëindiging niet toelaat. De onderzoeker zal er zorg voor dragen dat alle proefpersonen geïnformeerd worden.

7.2 Ernstige voorvallen

Ernstige voorvallen worden gedefinieerd als een ongewenste ervaring die zich tijdens de uitvoering van het onderzoek voordoet, al dan niet gerelateerd aan de interventie. Alle ernstige voorvallen die plotseling worden waargenomen door de deelnemer of door de onderzoeker worden gerapporteerd aan de METc.
Aangezien er geen geneesmiddelen getoetst worden in deze studie, worden er geen ernstige bijwerkingen verwacht. Ernstige ongewenste voorvallen kunnen plaatsvinden ten gevolge van de interventie, bijv. als gevolg van het niet (op tijd) doorverwijzen naar het ziekenhuis of het niet (op tijd) consulteren van een arts of specialist ouderengeneeskunde.

7.3 Follow-up van ernstige voorvallen

Alle schadelijke voorvallen worden in de gaten gehouden totdat het voorval teniet gedaan is, of totdat een stabiele situatie is bereikt. Afhankelijk van het voorval, zullen bij de follow-up extra testen of medische procedures uitgevoerd worden, en/of verwezen worden naar een huisarts of medisch specialist.

8 STATISTISCHE ANALYSE

8.1 Beschrijvende statistiek

Voor het weergeven van de kenmerken van de ouderen en mantelzorgers op de verschillende meetmomenten worden methoden voor beschrijvende statistiek gebruikt.

8.2 Univariate analyses

De samenhang tussen discrete variabelen wordt getoetst met de Chi-kwadraat toets (of Fisher exact indien van toepassing). Continue variabelen worden, afhankelijk van de normale verdeling van de variabelen, parametrisch getoetst met T-toetsen of ANOVA, of non-parametrisch met de Mann-Whitney U test. Ordinale variabelen worden getoetst met de Wilcoxon Rank-sum toets. Zowel verschillen tussen groepen als binnen groepen worden berekend.

8.3 Multivariate analyses

Effecten van de interventie worden berekend met logistische (ordinale variabelen) en hiërarchische (continue variabelen) regressieanalyses. De klinische relevantie van verschillen tussen experimentele en controlegroep wordt geschat met Cohen’s effect maten.

9 ETHISCHE ASPECTEN

9.1 Reglementen

De studie zal worden uitgevoerd volgens de Wet Medisch-wetenschappelijk Onderzoek met mensen (WMO).

9.2 Werving en toestemming

De procedure voor selectie, werving en inclusie is beschreven in Hoofdstuk 6.

9.2.1 Ouderen

De toestemmingsprocedure van de ouderen vindt als volgt plaats:
Nadat een oudere geselecteerd is uit het huisartsbestand (op basis van de inclusie- en exclusiecriteria) wordt hij/zij benaderd door de huisarts via een brief met het verzoek tot deelname aan de studie. De informatie in de brief is gericht op het doel, de inhoud, de duur en de voor- en nadelen van het onderzoek voor de deelnemers. Bij de informatie ontvangt men ook een informed consent formulier. De oudere wordt verzocht schriftelijk toestemming te verlenen aan het onderzoek door het formulier in te vullen en te ondertekenen. Onderdeel van de instemming betreft inzage in het medisch dossier van de huisarts, inzage in gegevens over zorggebruik en kosten bij de gemeente, bij de zorgverzekeraar en bij het zorgkantoor, toestemming dat informatie over gezondheid en zorgleefplan opgenomen worden in het medisch huisartsdossier van de oudere en (eventueel) toestemming voor benadering van een eventuele mantelzorger (alleen interventiegroep). De oudere heeft twee weken bedenktijd om te besluiten om wel of niet deel te nemen aan het onderzoek. Indien de oudere mee wil werken, dient hij/zij het informed consent formulier en de bijgevoegde vragenlijsten in te vullen en terug te sturen. Na deze twee weken ontvangen alle ouderen een herinnerings-/bedankbrief. Vijf weken na de eerste verzending ontvangen de non-respondenten nogmaals het hele informatiepakket met het informed consent formulier en de vragenlijsten. Ze hebben dan opnieuw twee weken de tijd om te reageren.
De verleende toestemming kan op ieder moment tijdens het onderzoek weer ingetrokken worden. Wanneer geen toestemming verleend wordt, of wanneer men zich terugtrekt, heeft dit geen gevolgen voor de verdere behandeling door de huisarts. Voor vragen over de studie, of voor hulp bij het invullen van de vragenlijst of het informed consent formulier kunnen de deelnemers bellen met de helpdesk of kunnen ze terecht bij een onafhankelijke arts.
Na randomisatie van de deelnemers over de interventie- en controlegroep volgen de thuisgesprekken door de casemanager en de ouderenadviseur (alleen interventiegroep). Tijdens deze eerste gesprekken wordt een anamnese afgenomen.

9.2.2 Mantelzorgers

De toestemmingsprocedure van de mantelzorgers vindt als volgt plaats:
Als tijdens de anamnese van de oudere in de interventiegroep blijkt dat de oudere zorg ontvangt van een mantelzorger, wordt aan de oudere gevraagd of hij/zij er mee akkoord gaat dat de mantelzorger wordt benaderd voor participatie in het onderzoek. Na toestemming van de oudere, wordt diens mantelzorger namens de huisarts benaderd voor deelname aan het onderzoek. Dit gebeurt op eenzelfde wijze als bij de ouderen. De mantelzorger ontvangt een brief namens de huisarts waarin gevraagd wordt of hij/zij wil deelnemen aan het onderzoek. Bijgevoegd zit een informatiebrief, een informed consent formulier en de vragenlijsten. Als de mantelzorger wil deelnemen, wordt hij/zij verzocht het informed consent formulier te ondertekenen en de bijgevoegde vragenlijsten

in te vullen en op te sturen. De mantelzorger heeft twee weken bedenktijd om te besluiten om wel of niet deel te nemen aan het onderzoek. Indien de mantelzorger mee wil werken, dient hij/zij het informed consent formulier en de bijgevoegde vragenlijsten in te vullen en terug te sturen. Na deze twee weken ontvangen alle mantelzorgers een herinnerings-/bedankbrief. Vijf weken na de eerste verzending ontvangen de non-respondenten nogmaals het hele informatiepakket met het informed consent formulier en de vragenlijsten. Ze hebben dan opnieuw twee weken de tijd om te reageren.
De verleende toestemming kan op ieder moment tijdens het onderzoek weer ingetrokken worden. Wanneer geen toestemming verleend wordt, heeft dit geen gevolgen voor de oudere. Voor vragen over de studie, of voor hulp bij het invullen van de vragenlijsten of het informed consent formulier kunnen de mantelzorgers bellen met de helpdesk of kunnen ze terecht bij een onafhankelijke arts.

9.3 Bezwaar door wilsonbekwame personen

Het zal naar verwachting niet (vaak) voorkomen dat een oudere wilsonbekwaam is, aangezien de ziekte(s) in de meeste gevallen dan dusdanig ernstig zijn dat een persoon niet meer thuis of in een verzorgingshuis kan wonen. Indien er toch een wilsonbekwaam persoon geïncludeerd kan worden, wordt aan de wettelijk vertegenwoordiger gevraagd of de oudere wel of niet wil deelnemen aan het onderzoek. De wettelijk vertegenwoordiger geeft vervolgens al dan niet toestemming voor deelname. Indien de wilsonbekwame oudere zelf aangeeft niet mee te willen doen aan het onderzoek (zich verzet), wordt het onderzoek bij deze persoon per direct stop gezet.

9.4 Voordelen en nadelen voor de respondenten (ouderen en mantelzorgers)

De voordelen voor de respondenten in de interventiegroep kunnen zijn:

1) De ouderen ontvangen proactieve en preventieve samenhangende zorg en begeleiding en hebben daarvoor een vast aanspreekpunt (het Ouderenzorg Team).
2) Naast de medische aspecten wordt ook expliciet aandacht besteed aan de psychosociale gevolgen van de gezondheidsproblematiek.
3) De ouderen ontvangen zorg op maat.
4) Er wordt aandacht besteed aan de zorglast en draagkracht van de mantelzorger.
5) De oudere en mantelzorger worden thuis bezocht.
6) De realisatie van het zorgleefplan wordt begeleid en geëvalueerd.

Een nadeel voor de oudere zou kunnen zijn dat de intensiteit van de interventie een te grote belasting vormt.
Een voordeel voor ouderen in de controlegroep kan zijn dat ze bijdragen aan onderzoek naar de verbetering van zorg voor ouderen. Een nadeel voor ouderen in de controlegroep kan zijn dat ze niet de keuze hebben om zorg volgens het nieuwe zorgmodel in het interventiejaar te ontvangen.

10 ADMINISTRATIEVE ASPECTEN EN PUBLICATIE

10.1 Verwerking en opslag van data en documenten

Ten behoeve van het onderzoek wordt per oudere en per mantelzorger een onderzoeksdossier aangelegd. Elke huisartspraktijk krijgt een eigen nummer, van 01 tot en met 15. Na de selectie wordt aan alle potentiële deelnemers een nummer toegekend, oplopend van 001 tot 3000 (of hoger). De combinatie van het huisartsnummer en het deelnemersnummer vormt een unieke code. Dit nummer wordt vermeld op het informed consent formulier. Hieraan wordt voor de oudere de letter O (Oudere) toegevoegd en voor de mantelzorger de letters MZ (mantelzorger). Het vooraf toegekende nummer blijft tijdens het hele onderzoek als respondentnummer gelden. Alle ingevulde anamneseformulieren, zorgplannen en overige formulieren met respondentgegevens worden aan het onderzoeksdossier van de oudere toegevoegd.

10.2 Amendementen

Amendementen zijn veranderingen die aangebracht zijn in het onderzoek naar aanleiding van een mening van de METc die de voorkeur heeft. Alle amendementen zullen aan de METc worden doorgegeven.
Ook alle wezenlijke amendementen zullen aan de METc doorgegeven worden. Niet-wezenlijke amendementen, zoals wijzigingen in contactgegevens van proefpersonen, typefouten en administratieve veranderingen, zullen niet aan de METc worden doorgegeven, maar zullen worden genoteerd en opgeslagen.

10.3 Jaarlijks voortgangsverslag

Het onderzoeksteam zal eenmalig, een jaar na de startdatum (begin 2013), een voortgangsverslag van het onderzoek aan de METc ter beschikking stellen. Hierin zal informatie worden gegeven over de datum van inclusie van de eerste proefpersoon, het aantal geïncludeerde proefpersonen, het aantal proefpersonen dat de interventie heeft afgemaakt, ernstige voorvallen, andere problemen en amendementen.

10.4 Eindverslag van de studie

De onderzoeker zal de METc inlichten aan het eind van de studie als de laatste proefpersoon de T1 meting gehad heeft. Deze laatste meting zal waarschijnlijk in februari 2013 afgerond worden. Begin april 2013 zal de onderzoeker de METc inlichten.
Indien de studie voortijdig beëindigd wordt, zal de onderzoeker de METc daarvan op de hoogte stellen evenals van de reden van beëindiging.
Binnen één jaar na het beëindigen van de studie zal de onderzoeker een onderzoeksverslag met de resultaten van de studie en eventuele publicaties en samenvattingen van de studie aan de METc overhandigen.

11 REFERENTIES

1. Jansen D, Cardol M: Mensen met een chronische ziekte of beperking hebben voor hun ondersteuning bijna altijd te maken met meerdere wettelijke regelingen. Utrecht; 2010.

2. Wagner EH, Austin BT, Von Korff M. Organizing care for patients with chronic illness. Milbank Q 1996; 74(4):511-544.

3. Wagner EH, Austin BT, Von Korff M. Improving outcomes in chronic illness. Manag Care Q 1996; 4(2):12-25.

4. Bodenheimer T, Wagner EH, Grumbach K. Improving primary care for patients with chronic illness. Jama-Journal of the American Medical Association 2002; 288(14):1775-1779.

5. Nolte E, McKee M. Integration and chronic care: a review. In: Nolte E, McKee M, editors. Caring for people with chronic conditions. A health system perspective. First ed. Maidenhead, Berkshire, England: 2008. 64-91.

6. Uittenbroek R, Vos M, Wynia K. Casemanagement voor ouderen met complexe zorgbehoeften. Eindrapport, 2009.

7. Wagner EH, Austin BT, Davis C, Hindmarsh M, Schaefer J, Bonomi A. Improving chronic illness care: translating evidence into action. Health Aff (Millwood) 2001; 20(6):64-78.

8. Bodenheimer T, Wagner EH, Grumbach K. Improving primary care for patients with chronic illness - The chronic care model, part 2. Jama-Journal of the American Medical Association 2002; 288(15):1909-1914.

9. Lemieux-Charles L, McGuire WL. What do we know about health care team effectiveness? A review of the literature. Med Care Res Rev 2006; 63(3):263-300.

10. Annerstedt L, Elmstahl S, Ingvad B, Samuelsson SM. Family caregiving in dementia - An analysis of the caregiver's burden and the "breaking-point" when home care becomes inadequate. Scandinavian Journal of Public Health 2000; 28(1):23-31.

11. Bedard M, Pedlar D, Martin NJ, Malott O, Stones MJ. Burden in caregivers of cognitively impaired older adults living in the community: Methodological issues and determinants. International Psychogeriatrics 2000; 12(3):307-332.

12. EuroQol Group. EuroQol - a new facility for the measurement of health related quality of life. Health Policy 1990; 16:199-208.

13. Schuurmans H, Steverink N, Lindenberg S, Frieswijk N, Slaets JPJ. Old or frail: What tells us more? Journals of Gerontology Series A-Biological Sciences and Medical Sciences 2004; 59(9):962-965.

14. Slaets JPJ. Vulnerability in the elderly: Frailty. Medical Clinics of North America 2006; 90(4):593-+.

15. Huyse FJ, Lyons JS, Stiefel F, Slaets J, De Jonge P, Latour C. Operationalizing the biopsychosocial model: the intermed. Psychosomatics 2001; 42(1):5-13.

16. De Jonge P, Huyse FJ, Slaets JP, Herzog T, Lobo A, Lyons JS et al. Care complexity in the general hospital: results from a European study. Psychosomatics 2001; 42(3):204-212.

17. De Jonge P, Huyse FJ, Stiefel FC, Slaets JP, Gans RO. INTERMED - a clinical instrument for biopsychosocial assessment. Psychosomatics 2001; 42(2):106-109.

18. Stiefel FC, Huyse FJ, Sollner W, Slaets JP, Lyons JS, Latour CH et al. Operationalizing integrated care on a clinical level: the INTERMED project. Med Clin North Am 2006; 90(4):713-758.

19. Huyse FJ, Lyons JS, Stiefel FC, Slaets JPJ, De Jonge P, Fink P et al. "INTERMED": A method to assess health service needs - I. Development and reliability. General Hospital Psychiatry 1999; 21(1):39-48.

20. Stiefel FC, De Jonge P, Huyse FJ, Guex P, Slaets JP, Lyons JS et al. "INTERMED": a method to assess health service needs. II. Results on its validity and clinical use. Gen Hosp Psychiatry 1999; 21(1):49-56.

21. Stiefel FC, De Jonge P, Huyse FJ, Slaets JP, Guex P, Lyons JS et al. INTERMED - an assessment and classification system for case complexity. Results in patients with low back pain. Spine (Phila Pa 1976) 1999; 24(4):378-384.

22. Gangi Herms AM, Pinggera GM, De Jonge P, Strasser H, Sollner W. Assessing health care needs and clinical outcome with urological case complexity: a study using INTERMED. Psychosomatics 2003; 44(3):196-203.

23. Rockwood K, Stolee P, Fox RA. Use of Goal Attainment Scaling in Measuring Clinically Important Change in the Frail Elderly. Journal of Clinical Epidemiology 1993; 46(10):1113-1118.

24. Rockwood K, Howlett S, Stadnyk K, Carver D, Powell C, Stolee P. Responsiveness of goal attainment scaling in a randomized controlled trial of comprehensive geriatric assessment. Journal of Clinical Epidemiology 2003; 56(8):736-743.

25. Van Exel NJA, Scholte op Reimer WJMS, Brouwer WBF, Van den Berg B, Koopmanschap MA, Van den Bos GAM. Instruments for assessing the burden of informal caregiving for stroke patients in clinical practice: a comparison of CSI, CRA, SCQ and self-rated burden. Clinical Rehabilitation 2004; 18:203-14.

26. Sullivan, MT. Caregiver Strain Index. Home Healthcare Nurse 2003; 21(3):197-198.

27. Glasgow RE, Wagner EH, Schaefer J, Mahoney LD, Reid RJ, Greene SM. Development and Validation of the Patient Assessment of Chronic Illness Care (PACIC). Med Care 2005; 43(5):436-44.

28. Vrijhoef HJ, Berbee R, Wagner EH, Steuten L. Quality of integrated chronic care measured by patient survey: identification, selection and application of most appropriate instruments. Health Expect. 2009;12(4):417-29.

29. Petkov J, Harvey P, Battersby M. The internal consistency and construct validity of the partners in health scale: validation of a patient rated chronic condition self-management measure. Qual Life Res. 2010 Sep;19(7):1079-85.

30. Battersby M, Harvey P, Mills PD, Kalucy E, Pols RG, Frith PA, McDonald P, Esterman A, Tsourtos G, Donato R, Pearce R, McGowan C. SA HealthPlus: a controlled trial of a statewide application of a generic model of chronic illness care. Milbank Q. 2007;85(1):37-67.

31. Borm GF, Hoogendoorn EH, Den HM, Zielhuis GA. Sequential balancing: a simple method for treatment allocation in clinical trials. Contemp Clin Trials 2005; 26(6):637-645.

32. Rovers MM, Straatman H, Zielhuis GA. Comparison of balanced and random allocation in clinical trials: a simulation study. Eur J Epidemiol 2000; 16(12):1123-1129.

33. Zielhuis GA, Straatman H, Van 't Hof-Grootenboer AE, Van Lier HJ, Rach GH, Van den BP. The choice of a balanced allocation method for a clinical trial in otitis media with effusion. Stat Med 1990; 9(3):237-246.

.
