# Supplementary material for: Effects of a population-based, person-centred and integrated care service on health, wellbeing and self-management of community-living older adults: A randomised controlled trial on Embrace
Source: PLoS One. 2018 Jan 19;13(1):e0190751. doi: 10.1371/journal.pone.0190751 (PMC5774687; doi:10.1371/journal.pone.0190751)
Supplement: S1 Table — Values are numbers (percentages) unless stated otherwise. (DOCX) [file pone.0190751.s004.docx]

**S1 Table. Characteristics of participants and non-participants. Values are numbers (percentages) unless stated otherwise.**

|  | **Participants**  **n=1456** | **Non-participants**  **n=1532** | **p** |
| --- | --- | --- | --- |
| Age in years, mean (SD) | 80.7 (4.6) | 82.5 (5.3) | <0.001 |
| Female | 799 (54.9) | 1012 (66.1) | <0.001 |
| Living in rural area | 259 (17.8) | 407 (26.6) | <0.001 |
