# Supplementary material for: Effects of a population-based, person-centred and integrated care service on health, wellbeing and self-management of community-living older adults: A randomised controlled trial on Embrace
Source: PLoS One. 2018 Jan 19;13(1):e0190751. doi: 10.1371/journal.pone.0190751 (PMC5774687; doi:10.1371/journal.pone.0190751)
Supplement: S2 Table — (DOCX) [file pone.0190751.s005.docx]

**S2 Table. Patient-reported outcomes at 12-month follow-up in the Embrace study: detailed results of the intention-to-treat multilevel analyses using data from the whole sample (n=1456).**

|  |  |  | **Embrace** | | | | **CAU** | | | | **Difference in change between Embrace and CAU** | | | | | |
| --- | --- | --- | --- | --- | --- | --- | --- | --- | --- | --- | --- | --- | --- | --- | --- | --- |
|  |  |  | (n=747) | | | | (n=709) | | | | (n=1456) | | | | | |
|  |  |  | T0 | | Change | | T0 | | Change | |  |  |  |  |  |  |
|  | Scale scores (range) | Higher score* | Mean | (SD) | Mean | (SD) | Mean | (SD) | Mean | (SD) | t | B | 95% CI | | p-value† | ES |
| **Health** |  |  |  |  |  |  |  |  |  |  |  |  |  |  |  |  |
| EQ-5D-3L | -0.33-1.00 | + | 0.79 | (0.15) | 0.00 | (0.11) | 0.78 | (0.16) | 0.00 | (0.11) | -0.43 | 0.00 | -0.01 to | 0.01 | 0.670 | 0.02 |
| EQ-VAS | 0-100 | + | 70.7 | (17.6) | -0.5 | (13.2) | 69.7 | (18.4) | -0.6 | (12.4) | 0.15 | 0.10 | -1.21 to | 1.42 | 0.878 | 0.01 |
| INTERMED-E-SA | 0-60 | - | 11.2 | (6.4) | -0.1 | (4.3) | 11.4 | (6.9) | -0.2 | (4.1) | 0.53 | 0.12 | -0.31 to | 0.54 | 0.597 | 0.03 |
| GFI | 0-15 | - | 3.9 | (2.8) | 0.1 | (1.7) | 4.0 | (2.9) | 0.1 | (1.7) | 0.00 | 0.00 | -0.18 to | 0.18 | 0.998 | 0.00 |
| Katz-15 | 0-15 | - | 1.77 | (2.40) | 0.35 | (1.51) | 1.94 | (2.66) | 0.19 | (1.47) | 1.98 | 0.15 | 0.00 to | 0.31 | **0.047** | 0.10 |
| PADL | 0-6 | - | 0.42 | (0.82) | 0.14 | (0.68) | 0.49 | (1.00) | 0.06 | (0.65) | 2.53 | 0.09 | 0.02 to | 0.16 | **0.011** | 0.13 |
| IADL | 0-7 | - | 1.16 | (1.57) | 0.19 | (0.95) | 1.24 | (1.62) | 0.13 | (0.94) | 1.33 | 0.07 | -0.03 to | 0.16 | 0.185 | 0.07 |
| **Wellbeing** |  |  |  |  |  |  |  |  |  |  |  |  |  |  |  |  |
| GWI SF Score | 0-1 | + | 0.86 | (0.19) | -0.02 | (0.16) | 0.86 | (0.18) | -0.02 | (0.16) | 0.14 | 0.00 | -0.02 to | 0.02 | 0.892 | 0.01 |
| QoL general | 0-5 | - | 2.77 | (0.92) | 0.08 | (0.74) | 2.78 | (0.93) | 0.10 | (0.73) | -0.47 | -0.02 | -0.09 to | 0.06 | 0.636 | 0.02 |
| QoL vs 1 year ago | 0-5 | - | 3.08 | (0.67) | 0.08 | (0.75) | 3.13 | (0.61) | 0.04 | (0.68) | 0.99 | 0.04 | -0.04 to | 0.11 | 0.320 | 0.05 |
| **Self-management** |  |  |  |  |  |  |  |  |  |  |  |  |  |  |  |  |
| SMAS-30 | 0-100 | + | 56.7 | (13.3) | -1.1 | (7.7) | 56.5 | (13.6) | -0.8 | (8.2) | -0.82 | -0.34 | -1.16 to | 0.48 | 0.411 | 0.04 |
| INIT | 0-100 | + | 55.2 | (17.3) | -2.3 | (12.1) | 55.2 | (17.1) | -2.5 | (11.8) | 0.37 | 0.23 | -0.99 to | 1.46 | 0.709 | 0.02 |
| SE | 0-100 | + | 60.1 | (17.3) | -0.8 | (12.1) | 60.0 | (18.0) | -0.9 | (12.4) | -0.75 | -0.43 | -1.55 to | 0.69 | 0.455 | 0.04 |
| INVEST | 0-100 | + | 39.7 | (20.1) | -1.1 | (13.6) | 38.7 | (19.6) | 0.0 | (13.7) | 0.25 | 0.16 | -1.10 to | 1.42 | 0.802 | 0.01 |
| POSITIV | 0-100 | + | 61.5 | (15.9) | -0.2 | (11.7) | 61.6 | (16.3) | 0.2 | (12.4) | -0.61 | -0.38 | -1.62 to | 0.85 | 0.542 | 0.03 |
| MULT | 0-100 | + | 74.2 | (13.2) | -0.8 | (10.9) | 74.5 | (14.4) | -0.4 | (11.0) | -1.54 | -1.10 | -2.50 to | 0.30 | 0.124 | 0.08 |
| VAR | 0-100 | + | 49.5 | (17.0) | -1.3 | (13.5) | 49.0 | (17.2) | -0.8 | (14.4) | -0.74 | -0.54 | -1.98 to | 0.90 | 0.461 | 0.04 |
| PIH-OA | 8-64 | + | 47.1 | (9.4) | 0.8 | (8.2) | 47.1 | (9.6) | 0.4 | (7.9) | 1.07 | 0.45 | -0.37 to | 1.28 | 0.285 | 0.06 |
| Knowledge | 2-16 | + | 10.1 | (3.8) | 0.8 | (3.4) | 10.2 | (3.7) | 0.3 | (3.6) | 2.61 | 0.48 | 0.12 to | 0.85 | **0.009** | 0.14 |
| Management | 2-16 | + | 12.5 | (3.4) | 0.1 | (3.6) | 12.4 | (3.5) | 0.0 | (3.4) | 0.40 | 0.07 | -0.28 to | 0.43 | 0.691 | 0.02 |
| Coping | 4-32 | + | 24.5 | (5.5) | 0.0 | (4.4) | 24.4 | (5.5) | 0.1 | (4.4) | -0.44 | -0.10 | -0.55 to | 0.35 | 0.659 | 0.02 |

CAU = Care as usual; EQ-5D-3L = EuroQol-5D-3L; EQ-VAS = EuroQoL-5D visual analogue scale; ES = Effect size *d,* thresholds <0.2 trivial, ≥ 0.2- 0.5 small, ≥0.5-0.8 medium, ≥ 0.8 large; GFI = Groningen Frailty Indicator; GWI SF Score = Groningen Well-being Indicator Satisfaction Score; IADL = Instrumental Activities of Daily Living; INIT = Taking initiatives subscale; INTERMED-E-SA = INTERMED for the Elderly Self-Assessment; INVEST = Investment behaviour subscale; MULT = Multi-functionality of resources subscale; PADL = Physical Activities of Daily Living; PIH-OA = Partners in Health scale for older adults; POSITIVE = Positive frame of mind subscale; QoL = Quality of life; SE = Self-efficacy beliefs subscale; SMAS-30 = Self-Management Ability Scale version 2; VAR = Variety in resources subscale.

* + Higher score means improvement; - higher score means deterioration.

† Values are corrected for age and sex; bold values indicate p<0.05.

**S2 Table. Legend**

| **Bold text and orange filling** | Significant (p<0.05) or clinically relevant (ES ≥0.20) deterioration |
| --- | --- |
| **Bold text and green filling** | Significant (p<0.05) or clinically relevant (ES ≥0.20) improvement |
