# Supplementary material for: Effects of a population-based, person-centred and integrated care service on health, wellbeing and self-management of community-living older adults: A randomised controlled trial on Embrace
Source: PLoS One. 2018 Jan 19;13(1):e0190751. doi: 10.1371/journal.pone.0190751 (PMC5774687; doi:10.1371/journal.pone.0190751)
Supplement: S3 Table — (DOCX) [file pone.0190751.s006.docx]

**S3 Table. Patient-reported outcomes at 12-month follow-up in the Embrace study: detailed results of the intention-to-treat multilevel analyses using data from participants with the risk profile Complex care needs (n=365).**

|  |  |  | **Embrace** | | | | **CAU** | | | | **Difference in change between Embrace and CAU** | | | | | |
| --- | --- | --- | --- | --- | --- | --- | --- | --- | --- | --- | --- | --- | --- | --- | --- | --- |
|  |  |  | (n=187) | | | | (n=178) | | | | (n=365) | | | | | |
|  |  |  | T0 | | Change | | T0 | | Change | |  |  |  |  |  |  |
|  | Scale scores (range) | Higher score* | Mean | (SD) | Mean | (SD) | Mean | (SD) | Mean | (SD) | t | B | 95% CI | | p-value† | ES |
| **Health** |  |  |  |  |  |  |  |  |  |  |  |  |  |  |  |  |
| EQ-5D-3L | -0.33-1.00 | + | 0.65 | (0.16) | -0.02 | (0.14) | 0.64 | (0.17) | -0.01 | (0.13) | -0.64 | -0.01 | -0.04 to | 0.02 | 0.521 | 0.07 |
| EQ-VAS | 0-100 | + | 56.8 | (16.8) | -0.1 | (13.6) | 53.8 | (19.4) | 1.6 | (16.5) | -0.99 | -1.54 | -4.60 to | 1.52 | 0.323 | 0.10 |
| INTERMED-E-SA | 0-60 | - | 19.6 | (4.6) | -1.9 | (4.9) | 20.6 | (5.2) | -2.6 | (5.0) | 1.44 | 0.75 | -0.27 to | 1.76 | 0.149 | 0.15 |
| GFI | 0-15 | - | 6.8 | (2.4) | 0.1 | (1.8) | 7.1 | (2.4) | 0.0 | (1.8) | 0.59 | 0.11 | -0.26 to | 0.48 | 0.552 | 0.06 |
| Katz-15 | 0-15 | - | 3.89 | (2.84) | 0.58 | (2.06) | 4.17 | (3.12) | 0.33 | (1.57) | 1.27 | 0.24 | -0.13 to | 0.62 | 0.204 | 0.13 |
| PADL | 0-6 | - | 0.91 | (1.16) | 0.32 | (0.98) | 1.13 | (1.33) | 0.14 | (0.78) | 1.90 | 0.17 | -0.01 to | 0.36 | 0.058 | 0.20 |
| IADL | 0-7 | - | 2.55 | (1.72) | 0.27 | (1.24) | 2.58 | (1.83) | 0.16 | (0.98) | 0.91 | 0.11 | -0.12 to | 0.34 | 0.363 | 0.10 |
| **Wellbeing** |  |  |  |  |  |  |  |  |  |  |  |  |  |  |  |  |
| GWI SF Score | 0-1 | + | 0.70 | (0.22) | -0.02 | (0.19) | 0.71 | (0.22) | -0.03 | (0.21) | 0.66 | 0.01 | -0.03 to | 0.05 | 0.512 | 0.07 |
| QoL general | 0-5 | - | 3.43 | (0.80) | 0.17 | (0.65) | 3.47 | (0.79) | 0.14 | (0.66) | 0.54 | 0.04 | -0.10 to | 0.17 | 0.587 | 0.06 |
| QoL vs 1 year ago | 0-5 | - | 3.45 | (0.81) | -0.04 | (0.91) | 3.51 | (0.71) | 0.01 | (0.69) | -0.72 | -0.06 | -0.23 to | 0.10 | 0.471 | 0.08 |
| **Self-management** |  |  |  |  |  |  |  |  |  |  |  |  |  |  |  |  |
| SMAS-30 | 0-100 | + | 47.7 | (14.8) | -2.0 | (8.2) | 47.0 | (14.0) | 0.2 | (9.1) | -2.43 | -2.17 | -3.93 to | -0.42 | **0.015** | **0.26** |
| INIT | 0-100 | + | 46.5 | (19.9) | -2.8 | (12.0) | 45.0 | (18.7) | -2.1 | (11.6) | -0.63 | -0.77 | -3.19 to | 1.64 | 0.530 | 0.07 |
| SE | 0-100 | + | 51.2 | (19.1) | -2.1 | (13.4) | 48.2 | (18.9) | 1.7 | (12.4) | -2.33 | -2.97 | -5.47 to | -0.47 | **0.020** | **0.24** |
| INVEST | 0-100 | + | 29.6 | (20.3) | -1.3 | (12.6) | 28.8 | (18.4) | 0.8 | (14.8) | -2.82 | -3.80 | -6.44 to | -1.15 | **0.005** | **0.30** |
| POSITIV | 0-100 | + | 49.9 | (17.6) | -0.2 | (12.0) | 51.7 | (15.5) | 1.2 | (13.5) | -1.23 | -1.62 | -4.19 to | 0.95 | 0.217 | 0.13 |
| MULT | 0-100 | + | 67.7 | (15.7) | -1.9 | (12.3) | 66.4 | (17.1) | 1.1 | (12.2) | -1.53 | -2.17 | -4.94 to | 0.61 | 0.126 | 0.16 |
| VAR | 0-100 | + | 41.6 | (18.2) | -3.2 | (12.9) | 41.6 | (17.3) | -1.3 | (14.0) | -1.35 | -1.89 | -4.62 to | 0.85 | 0.177 | 0.14 |
| PIH-OA | 8-64 | + | 42.8 | (9.2) | 1.1 | (8.2) | 41.7 | (9.8) | 1.1 | (8.0) | 0.03 | 0.03 | -1.63 to | 1.68 | 0.976 | 0.00 |
| Knowledge | 2-16 | + | 9.9 | (3.4) | 0.8 | (3.0) | 9.8 | (3.7) | 0.3 | (3.4) | 1.59 | 0.53 | -0.12 to | 1.18 | 0.113 | 0.17 |
| Management | 2-16 | + | 12.1 | (3.5) | 0.2 | (3.7) | 11.9 | (3.6) | 0.2 | (2.7) | -0.04 | -0.01 | -0.68 to | 0.65 | 0.969 | 0.00 |
| Coping | 4-32 | + | 20.8 | (5.6) | 0.1 | (4.7) | 20.0 | (5.7) | 0.6 | (5.0) | -0.96 | -0.49 | -1.48 to | 0.51 | 0.336 | 0.10 |

CAU = Care as usual; EQ-5D-3L = EuroQol-5D-3L; EQ-VAS = EuroQoL-5D visual analogue scale; ES = Effect size *d,* thresholds <0.2 trivial, ≥ 0.2- 0.5 small, ≥0.5-0.8 medium, ≥ 0.8 large; GFI = Groningen Frailty Indicator; GWI SF Score = Groningen Well-being Indicator Satisfaction Score; IADL = Instrumental Activities of Daily Living; INIT = Taking initiatives subscale; INTERMED-E-SA = INTERMED for the Elderly Self-Assessment; INVEST = Investment behaviour subscale; MULT = Multi-functionality of resources subscale; PADL = Physical Activities of Daily Living; PIH-OA = Partners in Health scale for older adults; POSITIVE = Positive frame of mind subscale; QoL = Quality of life; SE = Self-efficacy beliefs subscale; SMAS-30 = Self-Management Ability Scale version 2; VAR = Variety in resources subscale.

* + Higher score means improvement; - higher score means deterioration.

† Values are corrected for age and sex; bold values indicate p<0.05.

**S3 Table. Legend**

| **Bold text and orange filling** | Significant (p<0.05) or clinically relevant (ES ≥0.20) deterioration |
| --- | --- |
| **Bold text and green filling** | Significant (p<0.05) or clinically relevant (ES ≥0.20) improvement |
