# Supplementary material for: Effects of a population-based, person-centred and integrated care service on health, wellbeing and self-management of community-living older adults: A randomised controlled trial on Embrace
Source: PLoS One. 2018 Jan 19;13(1):e0190751. doi: 10.1371/journal.pone.0190751 (PMC5774687; doi:10.1371/journal.pone.0190751)
Supplement: S4 Table — (DOCX) [file pone.0190751.s007.docx]

**S4 Table. Patient-reported outcomes at 12-month follow-up in the Embrace study: detailed results of the intention-to-treat multilevel analyses using data from participants with the risk profile Frail (n=237).**

|  |  |  | **Embrace** | | | | **CAU** | | | | **Difference in change between Embrace and CAU** | | | | | |
| --- | --- | --- | --- | --- | --- | --- | --- | --- | --- | --- | --- | --- | --- | --- | --- | --- |
|  |  |  | (n=122) | | | | (n=115) | | | | (n=237) | | | | | |
|  |  |  | T0 | | Change | | T0 | | Change | |  |  |  |  |  |  |
|  | Scale scores (range) | Higher score* | Mean | (SD) | Mean | (SD) | Mean | (SD) | Mean | (SD) | t | B | 95% CI | | p-value† | ES |
| **Health** |  |  |  |  |  |  |  |  |  |  |  |  |  |  |  |  |
| EQ-5D-3L | -0.33-1.00 | + | 0.74 | (0.11) | -0.02 | (0.10) | 0.74 | (0.13) | 0.0 | (0.11) | -1.22 | -0.02 | -0.04 to | 0.01 | 0.223 | 0.16 |
| EQ-VAS | 0-100 | + | 67.2 | (15.7) | -1.7 | (14.3) | 70.0 | (13.7) | -3.0 | (11.4) | 0.86 | 1.45 | -1.84 to | 4.74 | 0.387 | 0.11 |
| INTERMED-E-SA | 0-60 | - | 11.5 | (3.2) | 1.4 | (4.2) | 10.9 | (3.3) | 1.3 | (3.6) | 0.51 | 0.25 | -0.70 to | 1.20 | 0.608 | 0.06 |
| GFI | 0-15 | - | 6.2 | (1.2) | -0.6 | (2.1) | 6.2 | (1.4) | -0.7 | (2.1) | 0.54 | 0.15 | -0.38 to | 0.67 | 0.586 | 0.07 |
| Katz-15 | 0-15 | - | 2.40 | (2.36) | 0.28 | (1.59) | 2.41 | (2.73) | 0.39 | (1.52) | -0.44 | -0.09 | -0.48 to | 0.31 | 0.660 | 0.06 |
| PADL | 0-6 | - | 0.49 | (0.81) | 0.14 | (0.66) | 0.59 | (1.17) | 0.10 | (0.58) | 0.58 | 0.05 | -0.11 to | 0.20 | 0.561 | 0.08 |
| IADL | 0-7 | - | 1.66 | (1.56) | 0.11 | (1.02) | 1.57 | (1.63) | 0.25 | (1.17) | -0.93 | -0.13 | -0.41 to | 0.15 | 0.355 | 0.12 |
| **Wellbeing** |  |  |  |  |  |  |  |  |  |  |  |  |  |  |  |  |
| GWI SF Score | 0-1 | + | 0.83 | (0.17) | -0.04 | (0.18) | 0.84 | (0.16) | -0.02 | (0.18) | -0.71 | -0.02 | -0.06 to | 0.03 | 0.478 | 0.09 |
| QoL general | 0-5 | - | 2.99 | (0.71) | 0.12 | (0.74) | 2.97 | (0.79) | 0.09 | (0.72) | 0.23 | 0.02 | -0.16 to | 0.21 | 0.818 | 0.03 |
| QoL vs 1 year ago | 0-5 | - | 3.02 | (0.63) | 0.11 | (0.80) | 3.03 | (0.59) | 0.17 | (0.76) | -0.80 | -0.08 | -0.28 to | 0.12 | 0.425 | 0.10 |
| **Self-management** |  |  |  |  |  |  |  |  |  |  |  |  |  |  |  |  |
| SMAS-30 | 0-100 | + | 53.6 | (9.1) | -0.4 | (7.5) | 54.8 | (11.5) | -0.7 | (8.4) | 0.38 | 0.39 | -1.64 to | 2.42 | 0.705 | 0.05 |
| INIT | 0-100 | + | 51.8 | (13.5) | -1.7 | (13.3) | 54.1 | (16.0) | -2.3 | (10.9) | 0.44 | 0.70 | -2.41 to | 3.81 | 0.658 | 0.06 |
| SE | 0-100 | + | 56.2 | (12.6) | 0.0 | (11.2) | 58.6 | (15.7) | -1.3 | (12.4) | 0.50 | 0.63 | -1.86 to | 3.12 | 0.619 | 0.07 |
| INVEST | 0-100 | + | 37.1 | (17.2) | -0.3 | (13.3) | 36.8 | (18.8) | 0.5 | (12.4) | 0.82 | 1.26 | -1.74 to | 4.26 | 0.412 | 0.11 |
| POSITIV | 0-100 | + | 58.2 | (12.5) | -0.3 | (11.4) | 58.0 | (15.8) | 0.5 | (13.2) | -0.41 | -0.66 | -3.79 to | 2.47 | 0.680 | 0.05 |
| MULT | 0-100 | + | 72.2 | (10.6) | -1.1 | (9.3) | 74.8 | (11.6) | -1.7 | (10.2) | -0.51 | -0.86 | -4.14 to | 2.43 | 0.609 | 0.07 |
| VAR | 0-100 | + | 46.4 | (14.5) | 1.2 | (12.9) | 46.6 | (16.2) | 0.3 | (14.3) | 0.75 | 1.32 | -2.11 to | 4.76 | 0.450 | 0.10 |
| PIH-OA | 8-64 | + | 44.7 | (9.3) | 1.7 | (9.1) | 48.0 | (8.7) | -0.8 | (7.7) | 2.33 | 2.54 | 0.40 to | 4.69 | **0.020** | **0.31** |
| Knowledge | 2-16 | + | 10.1 | (3.7) | 1.0 | (3.7) | 10.4 | (3.8) | -0.2 | (3.8) | 2.44 | 1.19 | 0.23 to | 2.14 | **0.015** | **0.32** |
| Management | 2-16 | + | 12.0 | (3.3) | 0.2 | (3.5) | 13.0 | (3.3) | -0.2 | (2.9) | 0.85 | 0.35 | -0.46 to | 1.16 | 0.398 | 0.11 |
| Coping | 4-32 | + | 22.7 | (5.2) | 0.6 | (5.2) | 24.6 | (4.6) | -0.4 | (4.5) | 1.56 | 0.99 | -0.26 to | 2.24 | 0.119 | 0.21 |

CAU = Care as usual; EQ-5D-3L = EuroQol-5D-3L; EQ-VAS = EuroQoL-5D visual analogue scale; ES = Effect size *d,* thresholds <0.2 trivial, ≥ 0.2- 0.5 small, ≥0.5-0.8 medium, ≥ 0.8 large; GFI = Groningen Frailty Indicator; GWI SF Score = Groningen Well-being Indicator Satisfaction Score; IADL = Instrumental Activities of Daily Living; INIT = Taking initiatives subscale; INTERMED-E-SA = INTERMED for the Elderly Self-Assessment; INVEST = Investment behaviour subscale; MULT = Multi-functionality of resources subscale; PADL = Physical Activities of Daily Living; PIH-OA = Partners in Health scale for older adults; POSITIVE = Positive frame of mind subscale; QoL = Quality of life; SE = Self-efficacy beliefs subscale; SMAS-30 = Self-Management Ability Scale version 2; VAR = Variety in resources subscale.

* + Higher score means improvement; - higher score means deterioration.

† Values are corrected for age and sex; bold values indicate p<0.05.

**S4 Table. Legend**

| **Bold text and orange filling** | Significant (p<0.05) or clinically relevant (ES ≥0.20) deterioration |
| --- | --- |
| **Bold text and green filling** | Significant (p<0.05) or clinically relevant (ES ≥0.20) improvement |
