# Supplementary material for: Effects of a population-based, person-centred and integrated care service on health, wellbeing and self-management of community-living older adults: A randomised controlled trial on Embrace
Source: PLoS One. 2018 Jan 19;13(1):e0190751. doi: 10.1371/journal.pone.0190751 (PMC5774687; doi:10.1371/journal.pone.0190751)
Supplement: S5 Table — (DOCX) [file pone.0190751.s008.docx]

**S5 Table. Patient-reported outcomes at 12-month follow-up in the Embrace study: detailed results of the intention-to-treat multilevel analyses using data from participants with the risk profile Robust (n=854).**

|  |  |  | **Embrace** | | | | **CAU** | | | | **Difference in change between Embrace and CAU** | | | | | |
| --- | --- | --- | --- | --- | --- | --- | --- | --- | --- | --- | --- | --- | --- | --- | --- | --- |
|  |  |  | (n=438) | | | | (n=416) | | | | (n=854) | | | | | |
|  |  |  | T0 | | Change | | T0 | | Change | |  |  |  |  |  |  |
|  | Scale scores (range) | Higher score* | Mean | (SD) | Mean | (SD) | Mean | (SD) | Mean | (SD) | t | B | 95% CI | | p-value† | ES |
| **Health** |  |  |  |  |  |  |  |  |  |  |  |  |  |  |  |  |
| EQ-5D-3L | -0.33-1.00 | + | 0.86 | (0.10) | 0.01 | (0.10) | 0.86 | (0.10) | 0.01 | (0.10) | 0.48 | 0.00 | -0.01 to | 0.02 | 0.630 | 0.03 |
| EQ-VAS | 0-100 | + | 77.7 | (14.3) | -0.4 | (12. 8) | 76.5 | (14.6) | -0.9 | (10.3) | 0.66 | 0.52 | -1.03 to | 2.07 | 0.511 | 0.05 |
| INTERMED-E-SA | 0-60 | - | 7.5 | (3.7) | 0.3 | (3. 7) | 7.6 | (3.9) | 0.5 | (3.4) | -0.61 | -0.15 | -0.63 to | 0.33 | 0.540 | 0.04 |
| GFI | 0-15 | - | 2.0 | (1.3) | 0.4 | (1.5) | 2.0 | (1.3) | 0.5 | (1.5) | -0.82 | -0.08 | -0.28 to | 0.11 | 0.411 | 0.06 |
| Katz-15 | 0-15 | - | 0.69 | (1.31) | 0.26 | (1.17) | 0.86 | (1.58) | 0.08 | (1.41) | 2.11 | 0.19 | 0.01 to | 0.36 | **0.035** | 0.14 |
| PADL | 0-6 | - | 0.19 | (0.47) | 0.07 | (0.47) | 0.18 | (0.55) | 0.01 | (0.60) | 1.70 | 0.06 | -0.01 to | 0.13 | 0.089 | 0.12 |
| IADL | 0-7 | - | 0.43 | (0.91) | 0.18 | (0.78) | 0.57 | (1.03) | 0.08 | (0.85) | 1.86 | 0.10 | -0.01 to | 0.21 | 0.063 | 0.13 |
| **Wellbeing** |  |  |  |  |  |  |  |  |  |  |  |  |  |  |  |  |
| GWI SF Score | 0-1 | + | 0.94 | (0.12) | -0.02 | (0.13) | 0.93 | (0.11) | -0.02 | (0.13) | 0.13 | 0.00 | -0.02 to | 0.02 | 0.900 | 0.01 |
| QoL general | 0-5 | - | 2.43 | (0.85) | 0.03 | (0.78) | 2.44 | (0.84) | 0.08 | (0.77) | -1.06 | -0.06 | -0.16 to | 0.05 | 0.289 | 0.07 |
| QoL vs 1 year ago | 0-5 | - | 2.93 | (0.55) | 0.13 | (0.64) | 3.00 | (0.50) | 0.02 | (0.65) | 2.37 | 0.11 | 0.02 to | 0.19 | **0.018** | 0.16 |
| **Self-management** |  |  |  |  |  |  |  |  |  |  |  |  |  |  |  |  |
| SMAS-30 | 0-100 | + | 61.4 | (11.3) | -0.9 | (7.5) | 61.0 | (11.6) | -1.2 | (7.8) | 0.43 | 0.23 | -0.79 to | 1.24 | 0.664 | 0.03 |
| INIT | 0-100 | + | 59.8 | (15.3) | -2.2 | (11.8) | 59.8 | (14.6) | -2.8 | (12.2) | 0.70 | 0.57 | -1.03 to | 2.18 | 0.485 | 0.05 |
| SE | 0-100 | + | 65.0 | (15.8) | -0.4 | (11.8) | 65.4 | (15.6) | -2.0 | (12.3) | 0.55 | 0.39 | -1.02 to | 1.80 | 0.585 | 0.04 |
| INVEST | 0-100 | + | 44.8 | (18.9) | -1.2 | (14.1) | 43.5 | (18.7) | -0.4 | (13.5) | 1.83 | 1.50 | -0.11 to | 3.11 | 0.068 | 0.13 |
| POSITIV | 0-100 | + | 67.4 | (12.7) | -0.1 | (11.6) | 66.9 | (14.5) | -0.3 | (11.6) | 0.21 | 0.16 | -1.38 to | 1.71 | 0.835 | 0.01 |
| MULT | 0-100 | + | 77.6 | (11.4) | -0.2 | (10.7) | 78.0 | (12.3) | -0.6 | (10.5) | -0.87 | -0.82 | -2.67 to | 1.02 | 0.383 | 0.06 |
| VAR | 0-100 | + | 53.7 | (15.7) | -1.2 | (13.8) | 52.8 | (16.2) | -0.8 | (14.6) | -0.45 | -0.44 | -2.35 to | 1.47 | 0.649 | 0.03 |
| PIH-OA | 8-64 | + | 49.6 | (8.6) | 0.4 | (8.0) | 49.1 | (8.9) | 0.4 | (7.8) | 0.08 | 0.04 | -1.01 to | 1.10 | 0.936 | 0.01 |
| Knowledge | 2-16 | + | 10.2 | (4.0) | 0.7 | (3.5) | 10.4 | (3.7) | 0.4 | (3.7) | 1.16 | 0.29 | -0.20 to | 0.77 | 0.245 | 0.08 |
| Management | 2-16 | + | 12.8 | (3.4) | -0.1 | (3.6) | 12.5 | (3.6) | -0.1 | (3. 7) | 0.04 | 0.01 | -0.47 to | 0.50 | 0.965 | 0.00 |
| Coping | 4-32 | + | 26.6 | (4.4) | -0.2 | (4.0) | 26.2 | (4.5) | 0.0 | (4.0) | -0.93 | -0.25 | -0.78 to | 0.28 | 0.355 | 0.06 |

CAU = Care as usual; EQ-5D-3L = EuroQol-5D-3L; EQ-VAS = EuroQoL-5D visual analogue scale; ES = Effect size *d,* thresholds <0.2 trivial, ≥ 0.2- 0.5 small, ≥0.5-0.8 medium, ≥ 0.8 large; GFI = Groningen Frailty Indicator; GWI SF Score = Groningen Well-being Indicator Satisfaction Score; IADL = Instrumental Activities of Daily Living; INIT = Taking initiatives subscale; INTERMED-E-SA = INTERMED for the Elderly Self-Assessment; INVEST = Investment behaviour subscale; MULT = Multi-functionality of resources subscale; PADL = Physical Activities of Daily Living; PIH-OA = Partners in Health scale for older adults; POSITIVE = Positive frame of mind subscale; QoL = Quality of life; SE = Self-efficacy beliefs subscale; SMAS-30 = Self-Management Ability Scale version 2; VAR = Variety in resources subscale.

* + Higher score means improvement; - higher score means deterioration.

† Values are corrected for age and sex; bold values indicate p<0.05.

**S5 Table. Legend**

| **Bold text and orange filling** | Significant (p<0.05) or clinically relevant (ES ≥0.20) deterioration |
| --- | --- |
| **Bold text and green filling** | Significant (p<0.05) or clinically relevant (ES ≥0.20) improvement |
