# Supplementary material for: Effects of a population-based, person-centred and integrated care service on health, wellbeing and self-management of community-living older adults: A randomised controlled trial on Embrace
Source: PLoS One. 2018 Jan 19;13(1):e0190751. doi: 10.1371/journal.pone.0190751 (PMC5774687; doi:10.1371/journal.pone.0190751)
Supplement: S6 Table — (DOCX) [file pone.0190751.s009.docx]

**S6 Table. Patient-reported outcomes at 12-month follow-up in the Embrace study: overview of the results of the complete case multilevel analyses for the whole sample and per risk profile.**

|  |  |  | **Whole sample** | | | | **Complex care needs** | | | | **Frail** | | | | **Robust** | | | |
| --- | --- | --- | --- | --- | --- | --- | --- | --- | --- | --- | --- | --- | --- | --- | --- | --- | --- | --- |
|  |  |  | (n=1456) | | | | (n=365) | | | | (n=237) | | | | (n=854) | | | |
|  |  |  | Embrace | CAU |  |  | Embrace | CAU |  |  | Embrace | CAU |  |  | Embrace | CAU |  |  |
|  | Scale scores (range) | Higher score* | Mean change | Mean change | p-value† | ES | Mean change | Mean change | p-value† | ES | Mean change | Mean change | p-value† | ES | Mean change | Mean change | p-value† | ES |
| **Health** |  |  |  |  |  |  |  |  |  |  |  |  |  |  |  |  |  |  |
| EQ-5D-3L | -0.33-1.00 | + | 0.02 | 0.07 | 0.202 | 0.08 | -0.02 | -0.01 | 0.501 | 0.08 | -0.02 | 0.00 | 0.192 | **0.20** | 0.04 | 0.11 | 0.282 | 0.08 |
| EQ-VAS | 0-100 | + | -0.4 | -0.6 | 0.922 | 0.01 | -0.5 | 2.0 | 0.322 | 0.13 | -1.6 | -2.9 | 0.437 | 0.12 | -0.1 | -0.9 | 0.435 | 0.06 |
| INTERMED-E-SA | 0-60 | - | 0.0 | -0.1 | 0.674 | 0.03 | -1.7 | -2.6 | 0.192 | 0.16 | 1.4 | 1.2 | 0.644 | 0.06 | 0.3 | 0.5 | 0.556 | 0.04 |
| GFI | 0-15 | - | 0.2 | 0.2 | 0.981 | 0.00 | 0.2 | 0.0 | 0.509 | 0.08 | -0.6 | -0.7 | 0.589 | 0.08 | 0.4 | 0.5 | 0.414 | 0.06 |
| Katz-15 | 0-15 | - | 0.38 | 0.16 | **0.021** | 0.15 | 0.70 | 0.36 | 0.230 | 0.17 | 0.38 | 0.40 | 0.912 | 0.02 | 0.28 | 0.03 | **0.015** | 0.19 |
| PADL | 0-6 | - | 0.18 | 0.06 | **0.009** | 0.16 | 0.48 | 0.17 | **0.020** | **0.31** | 0.17 | 0.15 | 0.645 | 0.08 | 0.07 | 0.01 | 0.172 | 0.11 |
| IADL | 0-7 | - | 0.23 | 0.13 | 0.104 | 0.10 | 0.41 | 0.23 | 0.297 | 0.14 | 0.16 | 0.29 | 0.526 | 0.10 | 0.19 | 0.06 | 0.044 | 0.16 |
| **Wellbeing** |  |  |  |  |  |  |  |  |  |  |  |  |  |  |  |  |  |  |
| GWI SF Score | 0-1 | + | -0.02 | -0.02 | 0.883 | 0.01 | -0.02 | -0.02 | 0.759 | 0.04 | -0.04 | -0.01 | 0.349 | 0.15 | -0.02 | -0.03 | 0.508 | 0.05 |
| QoL general | 0-5 | - | 0.07 | 0.10 | 0.595 | 0.02 | 0.19 | 0.14 | 0.631 | 0.06 | 0.12 | 0.08 | 0.883 | 0.02 | 0.02 | 0.09 | 0.263 | 0.09 |
| QoL vs 1 year ago | 0-5 | - | 0.09 | 0.04 | 0.318 | 0.06 | -0.05 | 0.03 | 0.469 | 0.09 | 0.09 | 0.17 | 0.404 | 0.12 | 0.14 | 0.01 | **0.018** | 0.18 |
| **Self-management** |  |  |  |  |  |  |  |  |  |  |  |  |  |  |  |  |  |  |
| SMAS-30 | 0-100 | + | -1.2 | -0.8 | 0.524 | 0.04 | -2.6 | 0.1 | **0.034** | **0.28** | -0.3 | -0.4 | 0.830 | 0.03 | -0.9 | -1.3 | 0.545 | 0.05 |
| INIT | 0-100 | + | -2.2 | -2.6 | 0.615 | 0.03 | -3.1 | -2.2 | 0.607 | 0.07 | -1.5 | -2.1 | 0.661 | 0.07 | -2.0 | -2.8 | 0.422 | 0.06 |
| SE | 0-100 | + | -0.9 | -0.4 | 0.471 | 0.04 | -2.4 | 1.5 | **0.033** | **0.27** | -1.0 | -1.7 | 0.685 | 0.06 | -0.3 | -0.7 | 0.619 | 0.04 |
| INVEST | 0-100 | + | -0.8 | -1.0 | 0.774 | 0.02 | -3.0 | 2.2 | **0.008** | **0.34** | 0.3 | -1.1 | 0.530 | 0.09 | -0.2 | -2.1 | 0.062 | 0.14 |
| POSITIV | 0-100 | + | -0.3 | 0.2 | 0.573 | 0.03 | -1.1 | 1.6 | 0.130 | 0.19 | -0.4 | 0.6 | 0.729 | 0.05 | 0.0 | -0.4 | 0.654 | 0.03 |
| MULT | 0-100 | + | -1.3 | 0.0 | 0.136 | 0.09 | -1.6 | 0.9 | 0.156 | 0.18 | -0.4 | 0.6 | 0.627 | 0.07 | -1.5 | -0.4 | 0.350 | 0.07 |
| VAR | 0-100 | + | -1.4 | -0.7 | 0.483 | 0.04 | -3.8 | -1.2 | 0.181 | 0.17 | 1.6 | 0.3 | 0.408 | 0.12 | -1.3 | -0.8 | 0.661 | 0.03 |
| PIH-OA | 8-64 | + | 0.7 | 0.3 | 0.393 | 0.05 | 1.3 | 1.1 | 0.899 | 0.02 | 1.8 | -1.1 | 0.051 | **0.31** | 0.3 | 0.3 | 0.966 | 0.00 |
| Knowledge | 2-16 | + | 0.8 | 0.2 | **0.011** | 0.15 | 0.9 | 0.1 | 0.107 | 0.21 | 1.1 | -0.3 | **0.018** | **0.36** | 0.7 | 0.4 | 0.255 | 0.09 |
| Management | 2-16 | + | 0.0 | 0.0 | 0.830 | 0.01 | 0.2 | 0.3 | 0.893 | 0.02 | 0.3 | -0.3 | 0.377 | 0.13 | -0.1 | 0.0 | 0.866 | 0.01 |
| Coping | 4-32 | + | 0.0 | 0.1 | 0.607 | 0.03 | 0.1 | 0.6 | 0.499 | 0.09 | 0.7 | -0.6 | 0.096 | **0.26** | -0.3 | 0.1 | 0.203 | 0.10 |

CAU = Care as usual; EQ-5D-3L = EuroQol-5D-3L; EQ-VAS = EuroQoL-5D visual analogue scale; ES = Effect size *d,* thresholds <0.2 trivial, ≥ 0.2- 0.5 small, ≥0.5-0.8 medium, ≥ 0.8 large; GFI = Groningen Frailty Indicator; GWI SF Score = Groningen Well-being Indicator Satisfaction Score; IADL = Instrumental Activities of Daily Living; INIT = Taking initiatives subscale; INTERMED-E-SA = INTERMED for the Elderly Self-Assessment; INVEST = Investment behaviour subscale; MULT = Multi-functionality of resources subscale; PADL = Physical Activities of Daily Living; PIH-OA = Partners in Health scale for older adults; POSITIVE = Positive frame of mind subscale; QoL = Quality of life; SE = Self-efficacy beliefs subscale; SMAS-30 = Self-Management Ability Scale version 2; VAR = Variety in resources subscale.

* + Higher score means improvement; - higher score means deterioration.

† Values are corrected for age and sex; bold values indicate p<0.05.

**S4 Table. Legend**

| **Bold text and orange filling** | Significant (p<0.05) or clinically relevant (ES ≥0.20) deterioration |
| --- | --- |
| **Bold text and green filling** | Significant (p<0.05) or clinically relevant (ES ≥0.20) improvement |
