# Supplementary material for: Effects of a population-based, person-centred and integrated care service on health, wellbeing and self-management of community-living older adults: A randomised controlled trial on Embrace
Source: PLoS One. 2018 Jan 19;13(1):e0190751. doi: 10.1371/journal.pone.0190751 (PMC5774687; doi:10.1371/journal.pone.0190751)
Supplement: S7 Table — (DOCX) [file pone.0190751.s010.docx]

**S7 Table. Patient-reported outcomes at 12-month follow-up in the Embrace study: detailed results of the complete case multilevel analyses using data from the whole sample (n=1456).**

|  |  |  | **Embrace** | | | | | | **CAU** | | | | | | **Difference in change between CAU and Embrace** | | | | | | |
| --- | --- | --- | --- | --- | --- | --- | --- | --- | --- | --- | --- | --- | --- | --- | --- | --- | --- | --- | --- | --- | --- |
|  |  |  | T0 | | | Change | | | T0 | | | Change | | |  |  |  |  |  |  |  |
|  | Scale scores (range) | Higher score* | n | Mean | (SD) | n | Mean | (SD) | n | Mean | (SD) | n | Mean | (SD) | n | t | B | 95% CI | | p-value† | ES |
| **Health** |  |  |  |  |  |  |  |  |  |  |  |  |  |  |  |  |  |  |  |  |  |
| EQ-5D-3L | -0.33-1.00 | + | 740 | 0.79 | (0.15) | 567 | 0.02 | (0.50) | 704 | 0.79 | (0.16) | 557 | 0.07 | (0.78) | 1124 | -1.28 | -0.05 | -0.13 to | 0.03 | 0.202 | 0.08 |
| EQ-VAS | 0-100 | + | 727 | 70.7 | (17.5) | 553 | -0.4 | (14.8) | 697 | 69.8 | (18.3) | 551 | -0.6 | (13.7) | 1104 | 0.10 | 0.08 | -1.60 to | 1.76 | 0.922 | 0.01 |
| INTERMED-E-SA | 0-60 | - | 747 | 11.2 | (6.4) | 569 | 0.0 | (4.8) | 709 | 11.4 | (6.9) | 561 | -0.1 | (4.6) | 1130 | 0.42 | 0.12 | -0.43 to | 0.67 | 0.674 | 0.03 |
| GFI | 0-15 | - | 747 | 3.9 | (2.8) | 569 | 0.2 | (2.0) | 709 | 4.0 | (2.9) | 561 | 0.2 | (1.9) | 1130 | 0.02 | 0.00 | -0.22 to | 0.23 | 0.981 | 0.00 |
| Katz-15 | 0-15 | - | 696 | 1.67 | (2.37) | 501 | 0.38 | (1.57) | 660 | 1.82 | (2.55) | 504 | 0.16 | (1.51) | 1005 | 2.31 | 0.22 | 0.03 to | 0.41 | **0.021** | 0.15 |
| PADL | 0-6 | - | 723 | 0.40 | (0.80) | 547 | 0.18 | (0.74) | 687 | 0.46 | (0.96) | 541 | 0.06 | (0.71) | 1088 | 2.60 | 0.11 | 0.03 to | 0.20 | **0.009** | 0.16 |
| IADL | 0-7 | - | 709 | 1.11 | (1.55) | 525 | 0.23 | (1.03) | 673 | 1.21 | (1.60) | 530 | 0.13 | (1.00) | 1055 | 1.63 | 0.10 | -0.02 to | 0.22 | 0.104 | 0.10 |
| **Wellbeing** |  |  |  |  |  |  |  |  |  |  |  |  |  |  |  |  |  |  |  |  |  |
| GWI SF Score | 0-1 | + | 666 | 0.86 | (0.19) | 502 | -0.02 | (0.17) | 626 | 0.86 | (0.18) | 488 | -0.02 | (0.18) | 990 | 0.15 | 0.00 | -0.02 to | 0.02 | 0.883 | 0.01 |
| QoL general | 0-5 | - | 744 | 2.77 | (0.92) | 569 | 0.07 | (0.85) | 708 | 2.78 | (0.93) | 557 | 0.10 | (0.82) | 1126 | -0.53 | -0.03 | -0.12 to | 0.07 | 0.595 | 0.02 |
| QoL vs 1 year ago | 0-5 | - | 744 | 3.08 | (0.67) | 569 | 0.09 | (0.85) | 708 | 3.13 | (0.61) | 560 | 0.04 | (0.76) | 1129 | 1.00 | 0.05 | -0.05 to | 0.14 | 0.318 | 0.06 |
| **Self-management** |  |  |  |  |  |  |  |  |  |  |  |  |  |  |  |  |  |  |  |  |  |
| SMAS-30 | 0-100 | + | 714 | 56.8 | (13.3) | 539 | -1.2 | (8.7) | 681 | 56.7 | (13.5) | 529 | -0.8 | (9.3) | 1068 | -0.64 | -0.35 | -1.43 to | 0.73 | 0.524 | 0.04 |
| INIT | 0-100 | + | 740 | 55.2 | (17.3) | 565 | -2.2 | (13.7) | 700 | 55.1 | (17.1) | 553 | -2.6 | (13.2) | 1118 | 0.50 | 0.41 | -1.18 to | 1.99 | 0.615 | 0.03 |
| SE | 0-100 | + | 740 | 74.2 | (13.1) | 562 | -0.9 | (12.4) | 704 | 74.7 | (14.3) | 558 | -0.4 | (12.3) | 1120 | -0.72 | -0.53 | -1.97 to | 0.91 | 0.471 | 0.04 |
| INVEST | 0-100 | + | 742 | 60.1 | (17.3) | 565 | -0.8 | (13.7) | 706 | 60.1 | (17.9) | 559 | -1.0 | (13.9) | 1124 | 0.29 | 0.24 | -1.38 to | 1.85 | 0.774 | 0.02 |
| POSITIV | 0-100 | + | 739 | 61.5 | (15.7) | 562 | -0.3 | (13.1) | 703 | 61.7 | (16.3) | 554 | 0.2 | (13.9) | 1116 | -0.56 | -0.45 | -2.03 to | 1.13 | 0.573 | 0.03 |
| MULT | 0-100 | + | 734 | 39.7 | (20.0) | 559 | -1.3 | (15.2) | 704 | 38.8 | (19.6) | 554 | 0.0 | (15.3) | 1113 | -1.49 | -1.36 | -3.15 to | 0.43 | 0.136 | 0.09 |
| VAR | 0-100 | + | 723 | 49.7 | (16.9) | 550 | -1.4 | (15.2) | 692 | 49.2 | (17.1) | 542 | -0.7 | (16.2) | 1092 | -0.70 | -0.67 | -2.53 to | 1.20 | 0.483 | 0.04 |
| PIH-OA | 8-64 | + | 711 | 47.2 | (9.3) | 524 | 0.7 | (9.3) | 667 | 47.2 | (9.4) | 512 | 0.3 | (8.8) | 1036 | 0.85 | 0.48 | -0.62 to | 1.58 | 0.393 | 0.05 |
| Knowledge | 2-16 | + | 729 | 10.1 | (3.8) | 552 | 0.8 | (3.9) | 696 | 10.3 | (3.7) | 541 | 0.2 | (4.0) | 1093 | 2.55 | 0.61 | 0.14 to | 1.08 | **0.011** | 0.15 |
| Management | 2-16 | + | 729 | 12.5 | (3.4) | 547 | 0.0 | (4.1) | 692 | 12.5 | (3.5) | 546 | 0.0 | (3.7) | 1093 | 0.21 | 0.05 | -0.41 to | 0.51 | 0.830 | 0.01 |
| Coping | 4-32 | + | 732 | 24.5 | (5.5) | 550 | 0.0 | (5.0) | 683 | 24.4 | (5.4) | 534 | 0.1 | (4.8) | 1084 | -0.51 | -0.15 | -0.74 to | 0.43 | 0.607 | 0.03 |

CAU = Care as usual; EQ-5D-3L = EuroQol-5D-3L; EQ-VAS = EuroQoL-5D visual analogue scale; ES = Effect size *d,* thresholds <0.2 trivial, ≥ 0.2- 0.5 small, ≥0.5-0.8 medium, ≥ 0.8 large; GFI = Groningen Frailty Indicator; GWI SF Score = Groningen Well-being Indicator Satisfaction Score; IADL = Instrumental Activities of Daily Living; INIT = Taking initiatives subscale; INTERMED-E-SA = INTERMED for the Elderly Self-Assessment; INVEST = Investment behaviour subscale; MULT = Multi-functionality of resources subscale; PADL = Physical Activities of Daily Living; PIH-OA = Partners in Health scale for older adults; POSITIVE = Positive frame of mind subscale; QoL = Quality of life; SE = Self-efficacy beliefs subscale; SMAS-30 = Self-Management Ability Scale version 2; VAR = Variety in resources subscale.

* + Higher score means improvement; - higher score means deterioration.

† Values are corrected for age and sex; bold values indicate p<0.05.

**S7 Table. Legend**

| **Bold text and orange filling** | Significant (p<0.05) or clinically relevant (ES ≥0.20) deterioration |
| --- | --- |
| **Bold text and green filling** | Significant (p<0.05) or clinically relevant (ES ≥0.20) improvement |
