# Supplementary material for: Effects of a population-based, person-centred and integrated care service on health, wellbeing and self-management of community-living older adults: A randomised controlled trial on Embrace
Source: PLoS One. 2018 Jan 19;13(1):e0190751. doi: 10.1371/journal.pone.0190751 (PMC5774687; doi:10.1371/journal.pone.0190751)
Supplement: S8 Table — (DOCX) [file pone.0190751.s011.docx]

**S8 Table. Patient-reported outcomes at 12-month follow-up in the Embrace study: detailed results of the complete case multilevel analyses using data from participants with the risk profile Complex care needs (n=365).**

|  |  |  | **Embrace** | | | | | | **CAU** | | | | | | **Difference in change between CAU and Embrace** | | | | | | |
| --- | --- | --- | --- | --- | --- | --- | --- | --- | --- | --- | --- | --- | --- | --- | --- | --- | --- | --- | --- | --- | --- |
|  |  |  | T0 | | | Change | | | T0 | | | Change | | |  |  |  |  |  |  |  |
|  | Scale scores (range) | Higher score* | n | Mean | (SD) | n | Mean | (SD) | n | Mean | (SD) | n | Mean | (SD) | n | t | B | 95% CI | | p-value† | ES |
| **Health** |  |  |  |  |  |  |  |  |  |  |  |  |  |  |  |  |  |  |  |  |  |
| EQ-5D-3L | -0.33-1.00 | + | 186 | 0.65 | (0.16) | 129 | -0.02 | (0.17) | 178 | 0.64 | (0.17) | 123 | -0.01 | (0.16) | 252 | -0.67 | -0.01 | -0.05 to | 0.03 | 0.501 | 0.08 |
| EQ-VAS | 0-100 | + | 182 | 56.7 | (16.7) | 124 | -0.5 | (16.1) | 172 | 53.8 | (19.4) | 120 | 2.0 | (19.9) | 244 | -0.99 | -2.26 | -6.75 to | 2.22 | 0.322 | 0.13 |
| INTERMED-E-SA | 0-60 | - | 187 | 19.6 | (4.6) | 129 | -1.7 | (5.9) | 178 | 20.6 | (5.2) | 123 | -2.6 | (6.0) | 252 | 1.31 | 0.97 | -0.49 to | 2.43 | 0.192 | 0.16 |
| GFI | 0-15 | - | 187 | 6.8 | (2.4) | 129 | 0.2 | (2.2) | 178 | 7.1 | (2.4) | 123 | 0.0 | (2.2) | 252 | 0.66 | 0.18 | -0.36 to | 0.72 | 0.509 | 0.08 |
| Katz-15 | 0-15 | - | 161 | 3.87 | (2.86) | 97 | 0.70 | (2.23) | 159 | 4.04 | (3.06) | 103 | 0.36 | (1.89) | 200 | 1.20 | 0.35 | -0.22 to | 0.92 | 0.230 | 0.17 |
| PADL | 0-6 | - | 175 | 0.90 | (1.15) | 119 | 0.48 | (1.11) | 170 | 1.10 | (1.32) | 117 | 0.17 | (0.93) | 236 | 2.35 | 0.31 | 0.05 to | 0.57 | **0.020** | **0.31** |
| IADL | 0-7 | - | 169 | 2.53 | (1.72) | 111 | 0.41 | (1.45) | 164 | 2.57 | (1.83) | 115 | 0.23 | (1.17) | 226 | 1.04 | 0.18 | -0.16 to | 0.52 | 0.297 | 0.14 |
| **Wellbeing** |  |  |  |  |  |  |  |  |  |  |  |  |  |  |  |  |  |  |  |  |  |
| GWI SF Score | 0-1 | + | 166 | 0.69 | (0.21) | 113 | -0.02 | (0.22) | 161 | 0.70 | (0.22) | 109 | -0.02 | (0.26) | 222 | 0.31 | 0.01 | -0.05 to | 0.07 | 0.759 | 0.04 |
| QoL general | 0-5 | - | 187 | 3.43 | (0.80) | 129 | 0.19 | (0.79) | 178 | 3.47 | (0.79) | 122 | 0.14 | (0.80) | 251 | 0.48 | 0.05 | -0.15 to | 0.24 | 0.631 | 0.06 |
| QoL vs 1 year ago | 0-5 | - | 187 | 3.45 | (0.81) | 129 | -0.05 | (1.10) | 178 | 3.51 | (0.71) | 123 | 0.03 | (0.83) | 252 | -0.72 | -0.09 | -0.33 to | 0.15 | 0.469 | 0.09 |
| **Self-management** |  |  |  |  |  |  |  |  |  |  |  |  |  |  |  |  |  |  |  |  |  |
| SMAS-30 | 0-100 | + | 177 | 47.8 | (15.0) | 122 | -2.6 | (9.9) | 170 | 47.4 | (14.0) | 114 | 0.1 | (11.0) | 236 | -2.13 | -2.86 | -5.50 to | -0.22 | **0.034** | **0.28** |
| INIT | 0-100 | + | 184 | 46.6 | (20.0) | 127 | -3.1 | (14.5) | 177 | 44.9 | (18.8) | 122 | -2.2 | (13.8) | 249 | -0.51 | -0.92 | -4.43 to | 2.59 | 0.607 | 0.07 |
| SE | 0-100 | + | 184 | 67.7 | (15.6) | 125 | -2.4 | (14.6) | 176 | 66.5 | (17.1) | 122 | 1.5 | (14.7) | 247 | -2.15 | -3.96 | -7.59 to | -0.33 | **0.033** | **0.27** |
| INVEST | 0-100 | + | 185 | 51.2 | (19.1) | 127 | -3.0 | (16.0) | 176 | 48.4 | (18.9) | 122 | 2.2 | (14.7) | 249 | -2.68 | -5.21 | -9.03 to | -1.38 | **0.008** | **0.34** |
| POSITIV | 0-100 | + | 185 | 50.1 | (17.3) | 127 | -1.1 | (13.8) | 177 | 51.7 | (15.6) | 121 | 1.6 | (16.3) | 248 | -1.52 | -2.83 | -6.51 to | 0.84 | 0.130 | 0.19 |
| MULT | 0-100 | + | 183 | 29.4 | (20.2) | 126 | -1.6 | (15.0) | 176 | 28.9 | (18.4) | 122 | 0.9 | (17.9) | 248 | -1.42 | -2.92 | -6.96 to | 1.12 | 0.156 | 0.18 |
| VAR | 0-100 | + | 180 | 41.7 | (18.2) | 125 | -3.8 | (15.5) | 171 | 42.1 | (17.2) | 116 | -1.2 | (16.7) | 241 | -1.34 | -2.74 | -6.78 to | 1.29 | 0.181 | 0.17 |
| PIH-OA | 8-64 | + | 177 | 42.8 | (9.2) | 116 | 1.3 | (9.8) | 161 | 42.0 | (9.5) | 107 | 1.1 | (9.5) | 223 | .128 | 0.16 | -2.35 to | 2.68 | 0.899 | 0.02 |
| Knowledge | 2-16 | + | 182 | 9.9 | (3.3) | 124 | 0.9 | (3.4) | 173 | 9.8 | (3.7) | 118 | 0.1 | (4.0) | 242 | 1.62 | 0.77 | -0.17 to | 1.72 | 0.107 | **0.21** |
| Management | 2-16 | + | 182 | 12.1 | (3.5) | 124 | 0.2 | (4.4) | 173 | 11.9 | (3.5) | 118 | 0.3 | (3.2) | 242 | -0.13 | -0.07 | -1.05 to | 0.92 | 0.893 | 0.02 |
| Coping | 4-32 | + | 182 | 20.7 | (5.6) | 121 | 0.1 | (5.6) | 167 | 20.0 | (5.7) | 113 | 0.6 | (6.1) | 234 | -0.68 | -0.51 | -2.00 to | 0.98 | 0.499 | 0.09 |

CAU = Care as usual; EQ-5D-3L = EuroQol-5D-3L; EQ-VAS = EuroQoL-5D visual analogue scale; ES = Effect size *d,* thresholds <0.2 trivial, ≥ 0.2- 0.5 small, ≥0.5-0.8 medium, ≥ 0.8 large; GFI = Groningen Frailty Indicator; GWI SF Score = Groningen Well-being Indicator Satisfaction Score; IADL = Instrumental Activities of Daily Living; INIT = Taking initiatives subscale; INTERMED-E-SA = INTERMED for the Elderly Self-Assessment; INVEST = Investment behaviour subscale; MULT = Multi-functionality of resources subscale; PADL = Physical Activities of Daily Living; PIH-OA = Partners in Health scale for older adults; POSITIVE = Positive frame of mind subscale; QoL = Quality of life; SE = Self-efficacy beliefs subscale; SMAS-30 = Self-Management Ability Scale version 2; VAR = Variety in resources subscale.

* + Higher score means improvement; - higher score means deterioration.

† Values are corrected for age and sex; bold values indicate p<0.05.

**S8 Table. Legend**

| **Bold text and orange filling** | Significant (p<0.05) or clinically relevant (ES ≥0.20) deterioration |
| --- | --- |
| **Bold text and green filling** | Significant (p<0.05) or clinically relevant (ES ≥0.20) improvement |
