# Supplementary material for: Effects of a population-based, person-centred and integrated care service on health, wellbeing and self-management of community-living older adults: A randomised controlled trial on Embrace
Source: PLoS One. 2018 Jan 19;13(1):e0190751. doi: 10.1371/journal.pone.0190751 (PMC5774687; doi:10.1371/journal.pone.0190751)
Supplement: S9 Table — (DOCX) [file pone.0190751.s012.docx]

**S9 Table. Patient-reported outcomes at 12-month follow-up in the Embrace study: detailed results of the complete case multilevel analyses using data from participants with the risk profile Frail (n=237).**

|  |  |  | **Embrace** | | | | | | **CAU** | | | | | | **Difference in change between CAU and Embrace** | | | | | | |
| --- | --- | --- | --- | --- | --- | --- | --- | --- | --- | --- | --- | --- | --- | --- | --- | --- | --- | --- | --- | --- | --- |
|  |  |  | T0 | | | Change | | | T0 | | | Change | | |  |  |  |  |  |  |  |
|  | Scale scores (range) | Higher score* | n | Mean | (SD) | n | Mean | (SD) | n | Mean | (SD) | n | Mean | (SD) | n | t | B | 95% CI | | p-value† | ES |
| **Health** |  |  |  |  |  |  |  |  |  |  |  |  |  |  |  |  |  |  |  |  |  |
| EQ-5D-3L | -0.33-1.00 | + | 121 | 0.74 | (0.11) | 94 | -0.02 | (0.11) | 113 | 0.74 | (0.13) | 88 | 0.00 | (0.12) | 182 | -1.31 | -0.02 | -0.06 to | 0.01 | 0.192 | **0.20** |
| EQ-VAS | 0-100 | + | 118 | 67.2 | (15.6) | 92 | -1.6 | (16.2) | 113 | 70.0 | (13.5) | 88 | -2.9 | (12.4) | 180 | 0.78 | 1.68 | -2.58 to | 5.95 | 0.437 | 0.12 |
| INTERMED-E-SA | 0-60 | - | 122 | 11.5 | (3.2) | 95 | 1.4 | (4.8) | 115 | 10.9 | (3.3) | 90 | 1.2 | (4.1) | 185 | 0.46 | 0.29 | -0.93 to | 1.50 | 0.644 | 0.06 |
| GFI | 0-15 | - | 122 | 6.2 | (1.2) | 95 | -0.6 | (2.4) | 115 | 6.2 | (1.4) | 90 | -0.7 | (2.4) | 185 | 0.54 | 0.19 | -0.49 to | 0.87 | 0.589 | 0.08 |
| Katz-15 | 0-15 | - | 112 | 2.36 | (2.40) | 82 | 0.38 | (1.73) | 109 | 2.24 | (2.57) | 80 | 0.40 | (1.57) | 162 | 0.11 | 0.03 | -0.49 to | 0.55 | 0.912 | 0.02 |
| PADL | 0-6 | - | 119 | 0.49 | (0.80) | 93 | 0.17 | (0.72) | 112 | 0.54 | (1.07) | 86 | 0.15 | (0.58) | 179 | 0.46 | 0.05 | -0.15 to | 0.24 | 0.645 | 0.08 |
| IADL | 0-7 | - | 114 | 1.61 | (1.58) | 85 | 0.16 | (1.07) | 110 | 1.53 | (1.62) | 84 | 0.29 | (1.27) | 169 | -0.64 | -0.12 | -0.47 to | 0.24 | 0.526 | 0.10 |
| **Wellbeing** |  |  |  |  |  |  |  |  |  |  |  |  |  |  |  |  |  |  |  |  |  |
| GWI SF Score | 0-1 | + | 108 | 0.83 | (0.17) | 82 | -0.04 | (0.21) | 100 | 0.85 | (0.14) | 78 | -0.01 | (0.19) | 160 | -0.94 | -0.03 | -0.09 to | 0.03 | 0.349 | 0.15 |
| QoL general | 0-5 | - | 122 | 2.99 | (0.71) | 95 | 0.12 | (0.84) | 114 | 2.96 | (0.79) | 89 | 0.08 | (0.81) | 184 | 0.15 | 0.02 | -0.22 to | 0.26 | 0.883 | 0.02 |
| QoL vs 1 year ago | 0-5 | - | 122 | 3.02 | (0.63) | 95 | 0.09 | (0.91) | 114 | 3.03 | (0.59) | 89 | 0.17 | (0.86) | 184 | -0.84 | -0.11 | -0.36 to | 0.15 | 0.404 | 0.12 |
| **Self-management** |  |  |  |  |  |  |  |  |  |  |  |  |  |  |  |  |  |  |  |  |  |
| SMAS-30 | 0-100 | + | 118 | 53.8 | (8.9) | 94 | -0.3 | (8.5) | 110 | 55.1 | (11.6) | 85 | -0.4 | (9.6) | 179 | 0.22 | 0.29 | -2.38 to | 2.96 | 0.830 | 0.03 |
| INIT | 0-100 | + | 121 | 51.7 | (13.4) | 95 | -1.5 | (15.3) | 114 | 53.9 | (16.0) | 90 | -2.1 | (12.4) | 185 | 0.44 | 0.91 | -3.17 to | 4.98 | 0.661 | 0.07 |
| SE | 0-100 | + | 121 | 72.1 | (10.4) | 95 | -1.0 | (10.5) | 114 | 74.8 | (11.6) | 90 | -1.7 | (11.5) | 185 | 0.41 | 0.66 | -2.54 to | 3.87 | 0.685 | 0.06 |
| INVEST | 0-100 | + | 121 | 56.1 | (12.5) | 95 | 0.3 | (12.7) | 114 | 58.6 | (15.7) | 89 | -1.1 | (14.0) | 184 | 0.63 | 1.24 | -2.65 to | 5.13 | 0.530 | 0.09 |
| POSITIV | 0-100 | + | 121 | 58.1 | (12.4) | 95 | -0.4 | (12.9) | 114 | 58.3 | (15.5) | 89 | 0.6 | (14.9) | 184 | -0.35 | -0.72 | -4.78 to | 3.35 | 0.729 | 0.05 |
| MULT | 0-100 | + | 119 | 37.2 | (17.0) | 94 | -0.4 | (15.0) | 113 | 36.9 | (18.9) | 88 | 0.6 | (13.9) | 182 | -0.49 | -1.05 | -5.31 to | 3.21 | 0.627 | 0.07 |
| VAR | 0-100 | + | 119 | 46.4 | (14.5) | 94 | 1.6 | (14.6) | 113 | 46.5 | (16.2) | 89 | 0.3 | (16.2) | 183 | 0.83 | 1.87 | -2.59 to | 6.33 | 0.408 | 0.12 |
| PIH-OA | 8-64 | + | 115 | 44.8 | (9.3) | 83 | 1.8 | (10.5) | 109 | 48.0 | (8.3) | 83 | -1.1 | (8.8) | 166 | 1.96 | 2.95 | -0.02 to | 5.91 | 0.051 | **0.31** |
| Knowledge | 2-16 | + | 119 | 10.1 | (3.7) | 92 | 1.1 | (4.1) | 113 | 10.4 | (3.8) | 89 | -0.3 | (4.3) | 181 | 2.39 | 1.50 | 0.26 to | 2.73 | **0.018** | **0.36** |
| Management | 2-16 | + | 117 | 12.0 | (3.2) | 90 | 0.3 | (3.9) | 113 | 13.1 | (3.1) | 89 | -0.3 | (3.3) | 179 | 0.89 | 0.46 | -0.56 to | 1.47 | 0.377 | 0.13 |
| Coping | 4-32 | + | 120 | 22.7 | (5.2) | 90 | 0.7 | (5.9) | 111 | 24.6 | (4.5) | 84 | -0.6 | (5.1) | 174 | 1.67 | 1.41 | -0.25 to | 3.07 | 0.096 | **0.26** |

CAU = Care as usual; EQ-5D-3L = EuroQol-5D-3L; EQ-VAS = EuroQoL-5D visual analogue scale; ES = Effect size *d,* thresholds <0.2 trivial, ≥ 0.2- 0.5 small, ≥0.5-0.8 medium, ≥ 0.8 large; GFI = Groningen Frailty Indicator; GWI SF Score = Groningen Well-being Indicator Satisfaction Score; IADL = Instrumental Activities of Daily Living; INIT = Taking initiatives subscale; INTERMED-E-SA = INTERMED for the Elderly Self-Assessment; INVEST = Investment behaviour subscale; MULT = Multi-functionality of resources subscale; PADL = Physical Activities of Daily Living; PIH-OA = Partners in Health scale for older adults; POSITIVE = Positive frame of mind subscale; QoL = Quality of life; SE = Self-efficacy beliefs subscale; SMAS-30 = Self-Management Ability Scale version 2; VAR = Variety in resources subscale.

* + Higher score means improvement; - higher score means deterioration.

† Values are corrected for age and sex; bold values indicate p<0.05.

**S9 Table. Legend**

| **Bold text and orange filling** | Significant (p<0.05) or clinically relevant (ES ≥0.20) deterioration |
| --- | --- |
| **Bold text and green filling** | Significant (p<0.05) or clinically relevant (ES ≥0.20) improvement |
