# Supplementary material for: Effects of a population-based, person-centred and integrated care service on health, wellbeing and self-management of community-living older adults: A randomised controlled trial on Embrace
Source: PLoS One. 2018 Jan 19;13(1):e0190751. doi: 10.1371/journal.pone.0190751 (PMC5774687; doi:10.1371/journal.pone.0190751)
Supplement: S10 Table — (DOCX) [file pone.0190751.s013.docx]

**S10 Table. Patient-reported outcomes at 12-month follow-up in the Embrace study: detailed results of the complete case multilevel analyses using data from participants with the risk profile Robust (n=854).**

|  |  |  | **Embrace** | | | | | | **CAU** | | | | | | **Difference in change between CAU and Embrace** | | | | | | |
| --- | --- | --- | --- | --- | --- | --- | --- | --- | --- | --- | --- | --- | --- | --- | --- | --- | --- | --- | --- | --- | --- |
|  |  |  | T0 | | | Change | | | T0 | | | Change | | |  |  |  |  |  |  |  |
|  | Scale scores (range) | Higher score* | n | Mean | (SD) | n | Mean | (SD) | n | Mean | (SD) | n | Mean | (SD) | n | t | B | 95% CI | | P-value† | ES |
| **Health** |  |  |  |  |  |  |  |  |  |  |  |  |  |  |  |  |  |  |  |  |  |
| EQ-5D-3L | -0.33-1.00 | + | 433 | 0.86 | (0.10) | 344 | 0.04 | (0.63) | 413 | 0.86 | (0.10) | 346 | 0.11 | (0.98) | 690 | -1.08 | -0.07 | -0.19 to | 0.06 | 0.282 | 0.08 |
| EQ-VAS | 0-100 | + | 427 | 77.7 | (14.1) | 337 | -0.1 | (13.9) | 412 | 76.5 | (14.5) | 343 | -0.9 | (11.1) | 680 | 0.78 | 0.75 | -1.13 to | 2.62 | 0.435 | 0.06 |
| INTERMED-E-SA | 0-60 | - | 438 | 7.5 | (3.7) | 345 | 0.3 | (4.22) | 416 | 7.6 | (3.9) | 348 | 0.5 | (3.7) | 693 | -0.59 | -0.18 | -0.77 to | 0.41 | 0.556 | 0.04 |
| GFI | 0-15 | - | 438 | 2.0 | (1.3) | 345 | 0.4 | (1.7) | 416 | 2.0 | (1.3) | 348 | 0.5 | (1.6) | 693 | -0.82 | -0.10 | -0.34 to | 0.14 | 0.414 | 0.06 |
| Katz-15 | 0-15 | - | 423 | 0.65 | (1.27) | 322 | 0.28 | (1.24) | 392 | 0.80 | (1.51) | 321 | 0.03 | (1.34) | 643 | 2.43 | 0.25 | 0.05 to | 0.45 | **0.015** | 0.19 |
| PADL | 0-6 | - | 429 | 0.18 | (0.46) | 335 | 0.07 | (0.52) | 405 | 0.17 | (0.53) | 338 | 0.01 | (0.65) | 673 | 1.37 | 0.06 | -0.03 to | 0.15 | 0.172 | 0.11 |
| IADL | 0-7 | - | 426 | 0.42 | (0.89) | 329 | 0.19 | (0.82) | 399 | 0.56 | (1.02) | 331 | 0.06 | (0.85) | 660 | 2.01 | 0.13 | 0.00 to | 0.26 | **0.044** | 0.16 |
| **Wellbeing** |  |  |  |  |  |  |  |  |  |  |  |  |  |  |  |  |  |  |  |  |  |
| GWI SF Score | 0-1 | + | 392 | 0.94 | (0.12) | 307 | -0.02 | (0.14) | 365 | 0.94 | (0.10) | 301 | -0.03 | (0.13) | 608 | 0.66 | 0.01 | -0.01 to | 0.03 | 0.508 | 0.05 |
| QoL general | 0-5 | - | 435 | 2.43 | (0.84) | 345 | 0.02 | (0.87) | 416 | 2.44 | (0.84) | 346 | 0.09 | (0.84) | 691 | -1.12 | -0.07 | -0.20 to | 0.05 | 0.263 | 0.09 |
| QoL vs 1 year ago | 0-5 | - | 435 | 2.93 | (0.55) | 345 | 0.14 | (0.72) | 416 | 3.00 | (0.50) | 348 | 0.01 | (0.71) | 693 | 2.37 | 0.13 | 0.02 to | 0.24 | **0.018** | 0.18 |
| **Self-management** |  |  |  |  |  |  |  |  |  |  |  |  |  |  |  |  |  |  |  |  |  |
| SMAS-30 | 0-100 | + | 419 | 61.5 | (11.2) | 323 | -0.9 | (8.3) | 401 | 61.1 | (11.6) | 330 | -1.3 | (8.5) | 653 | 0.61 | 0.39 | -0.88 to | 1.67 | 0.545 | 0.05 |
| INIT | 0-100 | + | 435 | 59.9 | (15.2) | 343 | -2.0 | (13.0) | 409 | 59.9 | (14.4) | 341 | -2.8 | (13.2) | 684 | 0.80 | 0.80 | -1.16 to | 2.77 | 0.422 | 0.06 |
| SE | 0-100 | + | 435 | 77.6 | (11.4) | 342 | -0.3 | (12.0) | 414 | 78.1 | (12.1) | 346 | -0.7 | (11.5) | 688 | 0.50 | 0.44 | -1.30 to | 2.18 | 0.619 | 0.04 |
| INVEST | 0-100 | + | 436 | 65.0 | (15.8) | 343 | -0.2 | (13.0) | 416 | 65.4 | (15.6) | 348 | -2.1 | (13.4) | 691 | 1.87 | 1.87 | -0.10 to | 3.83 | 0.062 | 0.14 |
| POSITIV | 0-100 | + | 433 | 67.4 | (12.6) | 340 | 0.0 | (12.9) | 412 | 66.9 | (14.5) | 344 | -0.4 | (12.6) | 684 | 0.45 | 0.43 | -1.46 to | 2.33 | 0.654 | 0.03 |
| MULT | 0-100 | + | 432 | 44.8 | (18.9) | 339 | -1.5 | (15.4) | 415 | 43.5 | (18.7) | 344 | -0.4 | (14.7) | 683 | -0.94 | -1.07 | -3.32 to | 1.18 | 0.350 | 0.07 |
| VAR | 0-100 | + | 424 | 54.0 | (15.5) | 331 | -1.3 | (15.1) | 408 | 52.9 | (16.2) | 337 | -0.8 | (16.1) | 668 | -0.44 | -0.53 | -2.89 to | 1.84 | 0.661 | 0.03 |
| PIH-OA | 8-64 | + | 419 | 49.7 | (8.5) | 325 | 0.3 | (8.7) | 397 | 49.1 | (8.8) | 322 | 0.3 | (8.6) | 647 | -0.04 | -0.03 | -1.36 to | 1.30 | 0.966 | 0.00 |
| Knowledge | 2-16 | + | 428 | 10.2 | (4.0) | 336 | 0.7 | (4.0) | 410 | 10.4 | (3.7) | 334 | 0.4 | (4.0) | 670 | 1.14 | 0.35 | -0.25 to | 0.95 | 0.255 | 0.09 |
| Management | 2-16 | + | 430 | 12.8 | (3.4) | 333 | -0.1 | (4.0) | 406 | 12.5 | (3.5) | 339 | 0.0 | (4.0) | 672 | -0.17 | -0.05 | -0.66 to | 0.55 | 0.866 | 0.01 |
| Coping | 4-32 | + | 430 | 26.6 | (4.4) | 339 | -0.3 | (4.4) | 405 | 26.2 | (4.4) | 337 | 0.1 | (4.3) | 676 | -1.274 | -0.42 | -1.08 to | 0.23 | 0.203 | 0.10 |

CAU = Care as usual; EQ-5D-3L = EuroQol-5D-3L; EQ-VAS = EuroQoL-5D visual analogue scale; ES = Effect size *d,* thresholds <0.2 trivial, ≥ 0.2- 0.5 small, ≥0.5-0.8 medium, ≥ 0.8 large; GFI = Groningen Frailty Indicator; GWI SF Score = Groningen Well-being Indicator Satisfaction Score; IADL = Instrumental Activities of Daily Living; INIT = Taking initiatives subscale; INTERMED-E-SA = INTERMED for the Elderly Self-Assessment; INVEST = Investment behaviour subscale; MULT = Multi-functionality of resources subscale; PADL = Physical Activities of Daily Living; PIH-OA = Partners in Health scale for older adults; POSITIVE = Positive frame of mind subscale; QoL = Quality of life; SE = Self-efficacy beliefs subscale; SMAS-30 = Self-Management Ability Scale version 2; VAR = Variety in resources subscale.

* + Higher score means improvement; - higher score means deterioration.

† Values are corrected for age and sex; bold values indicate p<0.05.

**S10 Table. Legend**

| **Bold text and orange filling** | Significant (p<0.05) or clinically relevant (ES ≥0.20) deterioration |
| --- | --- |
| **Bold text and green filling** | Significant (p<0.05) or clinically relevant (ES ≥0.20) improvement |
